# Cell Competition Modifies Adult Stem Cell and Tissue Population Dynamics in a JAK-STAT-Dependent Manner

**DOI:** 10.1016/j.devcel.2015.06.010

**Published:** 2015-08-10

**Authors:** Golnar Kolahgar, Saskia J.E. Suijkerbuijk, Iwo Kucinski, Enzo Z. Poirier, Sarah Mansour, Benjamin D. Simons, Eugenia Piddini

**Affiliations:** 1 The Wellcome Trust/Cancer Research UK Gurdon Institute, University of Cambridge, Tennis Court Road, Cambridge CB2 1QN, UK

## Abstract

Throughout their lifetime, cells may suffer insults that reduce their fitness and disrupt their function, and it is unclear how these potentially harmful cells are managed in adult tissues. We address this question using the adult *Drosophila* posterior midgut as a model of homeostatic tissue and ribosomal *Minute* mutations to reduce fitness in groups of cells. We take a quantitative approach combining lineage tracing and biophysical modeling and address how cell competition affects stem cell and tissue population dynamics. We show that healthy cells induce clonal extinction in weak tissues, targeting both stem and differentiated cells for elimination. We also find that competition induces stem cell proliferation and self-renewal in healthy tissue, promoting selective advantage and tissue colonization. Finally, we show that winner cell proliferation is fueled by the JAK-STAT ligand Unpaired-3, produced by *Minute*^−/+^ cells in response to chronic JNK stress signaling.

## Highlights

• In the adult fly gut, wild-type cells outcompete subfit *Minute*^−/+^ cells
• Both stem and differentiated *Minute*^−/+^ cells are eliminated by cell competition
• Cell competition promotes proliferation and self-renewal of normal stem cells
• The growth of healthy cells is boosted by JAK-STAT signaling

## Graphical Abstract

**Figure:**
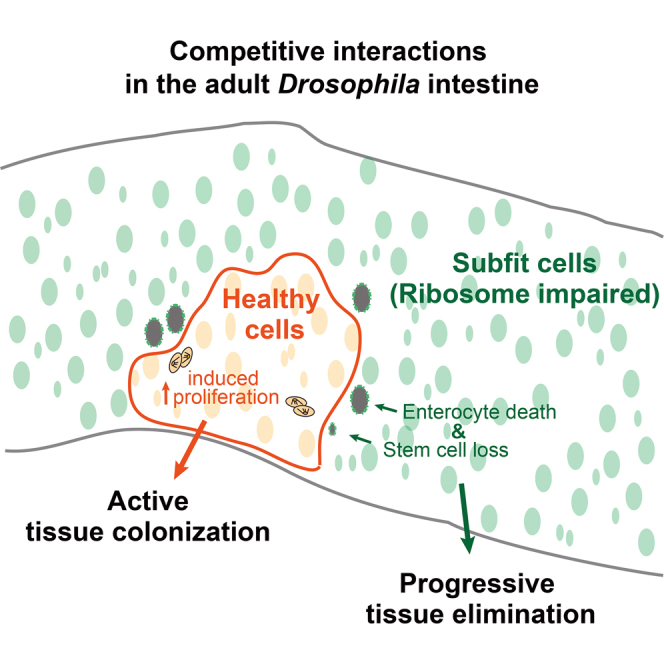

## Teaser

When cells within a tissue suffer insults that reduce their fitness, it is unclear how they are then managed. Kolahgar et al. provide evidence for active elimination of subfit cells in the adult fly gut through cell competition and show that normal cells increase tissue colonization by JAK-STAT signaling activation.

## Introduction

In adult animals, homeostatic tissues are composed of stem cells and differentiated cells that carry out specific tissue functions. The lifetime of these cells is, with few exceptions, orders of magnitude shorter than the lifetime of the individual in which they live. Thus, at any given time, adult organisms contain a proportion of cells that, either simply due to age or because of accidental damage or mutation, may be functioning suboptimally and may therefore contribute less effectively to healthy tissue function. It is normally assumed that these cells are turned over naturally when they spontaneously die. But is this really the case? How do adult tissues respond when suboptimal cells are present, and how does tissue colonization vary in the presence of heterogeneous cell communities?

It is well established that in developing tissues, cells compare their fitness with their neighbors, and less fit (“loser”) cells are eliminated through a phenomenon known as cell competition (Morata and Ripoll, 1975; de Beco et al., 2012; Vivarelli et al., 2012; Vincent et al., 2013). This likely acts as a quality control mechanism that eliminates less fit cells before they can contribute to the adult organism. Recent evidence shows that cell competition can also occur in adult tissues. For example, liver repopulation experiments show that embryonic liver cells take over adult liver tissue through a mechanism akin to cell competition (Oertel et al., 2006; Menthena et al., 2011). Similarly, cell competition has been observed between wild-type cells and cells overexpressing *myc* in the mouse heart (Villa del Campo et al., 2014). In addition, this phenomenon has been observed in some adult niche compartments (Jin et al., 2008; Issigonis et al., 2009; Rhiner et al., 2009; Bondar and Medzhitov, 2010; Marusyk et al., 2010), and a recent report suggests that it may also be taking place in adult fly tissues (Merino et al., 2015). However, how cell competition affects adult tissue dynamics and stem cell behavior has been little explored so far. In this study, we took advantage of the simplicity and genetic tractability of a well-defined model of adult homeostatic tissue, the *Drosophila* adult posterior midgut, to study the effect of cell competition on stem and differentiated cells and its consequences on tissue-level population dynamics.

The adult *Drosophila* posterior midgut in recent years has proven to be a powerful system to study adult stem cell behavior, tissue homeostasis, aging, and regeneration (Micchelli and Perrimon, 2006; Ohlstein and Spradling, 2006, 2007; Jiang and Edgar, 2012). This increasingly well characterized organ has high cellular turnover and is maintained in a way that is remarkably similar to the mammalian intestine: enterocytes (ECs) and enteroendocrine cells (EEs), which form the wall of the intestinal tube, turn over rapidly and are maintained by a supply of newly differentiated cells produced from more basally located intestinal stem cells (ISCs) (Micchelli and Perrimon, 2006; Ohlstein and Spradling, 2006, 2007; Jiang and Edgar, 2012).

As a means to reduce cellular fitness, we used mutations in ribosomal genes (known as *Minute* in *Drosophila*; Marygold et al., 2007), because they are potent inducers of cell competition in developing tissues. Indeed, it is well established that in growing imaginal discs, cells that are heterozygous mutant for *Minute* (*M*^−/+^) are effectively eliminated by wild-type cells (Morata and Ripoll, 1975; de Beco et al., 2012; Vivarelli et al., 2012; Vincent et al., 2013). *M*^−/+^ mutants also have the added advantage that they are among the few loser mutations that lead to viable adults, facilitating our study.

In this work, we identify the cellular parameters that are affected by cell competition in adult tissues. We find that cell competition affects both stem cells and differentiated cells and impacts on several aspects of cell behavior, namely cell survival, proliferation, and stem cell self-renewal. We also show that the clonal expansion of fitter cells is fueled by chronic activation of Jun N-terminal kinase (JNK) and Janus kinase (JAK)-signal transducer and activator of transcription (STAT) signaling pathways in unfit *M*^−/+^ intestinal cells.

## Results

### Healthy Cells Induce Delamination and Apoptosis of Subfit Differentiated Enterocytes

**Figure 1:**
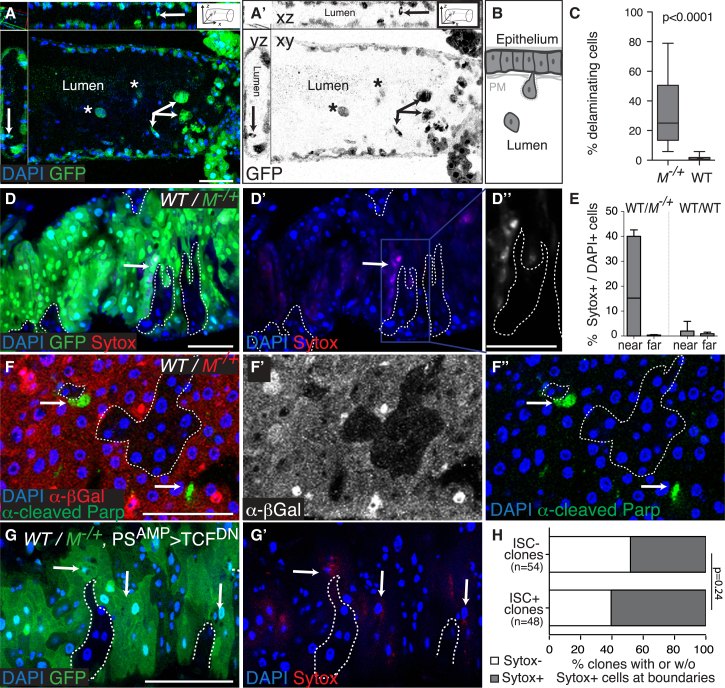
Wild-Type Cells Induce Accelerated Turnover of *M*^−/+^ Intestinal Cells (A and A′) Posterior midgut cross-sections along the xy, xz, and yz axes showing the epithelial walls and intestinal lumen. *M*^−/+^ clones (2×GFP, arrows) were generated in a pseudo-WT background by *hs*-Flp recombination (WT; 1×GFP, asterisks) (genotype: *Df(1)R194, w/hsflp; FRT40, ubiGFP/P[RpL36+w+], FRT40*). Delaminated cells seen in the lumen were assigned to the WT or *M*^−/+^ population based on GFP intensity, shown in grayscale (A′). (B) Cartoon depicting epithelial cells delaminating from the epithelium into the gut lumen. PM, peritrophic matrix. (C) Box plots displaying the percentage of delaminating cells throughout the 3D volume of the gut lumen for WT or *M*^−/+^ cells in guts as in (A) (n = 21 guts; p: Mann-Whitney test). (D–D″) *M*^−/+^ posterior midgut harboring *hs*-Flp-induced WT clones (GFP-negative) stained with the cell-death label Sytox (red in D and D′ [arrows]; gray in D″) (*hsflp/+; FRT82B, ubiGFP, RpS3/FRT82B*). (E) Box plots displaying the percentage of *M*^−/+^ Sytox^+^ cells among the cells near (less than two rows of cells) or far (more than two rows of cells) from WT clones (n = 11 guts; genotype as in D) or the percentage of Sytox^+^ cells in control WT cells near or far from WT clones (n = 12 guts; genotype: *hsflp/+; +/CyO; FRT82B, ubiGFP/FRT82B*) (p = 0.03 for *WT/M*^−/+^ and p = 0.52 for *WT/WT*, Mann-Whitney test). (F–F″) Cleaved PARP detection (green; arrows) reveals frequent apoptosis in *M*^−/+^ cells bordering WT cells (β-Gal-negative) (*hsflp/+; FRT40, M(2), arm-lacZ/FRT40; actGal4, UAS mCD8 Parp Venus/+*). (G and H) *M*^−/+^ posterior midgut harboring *hs*-Flp-induced WT clones (G and G′). Clones were left to grow for 4 days ACI and then TCF^DN^ was expressed continually for 5–6 days across progenitor cells (*PSwitch*^*amp*^*>TCF*^*DN*^, +RU486) to induce ISC differentiation (*hsflp/+; PSwitch*^*AMP*^*/UAS TCF*^*DN*^*; FRT82B, ubiGFP, RpS3/FRT82B*). Clones were scored for the presence or absence of ISCs (scoring small DAPI^+^ cells as a proxy for ISCs) and further analyzed for the presence of Sytox^+^*M*^−/+^ cells at clone boundaries (arrows in G and G′) (n = 52 ISC^−^ clones; n = 48 ISC^+^ clones). Differences in the distribution of clones between these two categories were not significantly different (p = 0.24, Fisher’s exact test) (H). Scale bars represent 50 μm. Genotypes are indicated in parentheses throughout the figure legends. Clones (defined in the z volume) are marked with dotted lines throughout the figures. See also Figure S1.

Induction of death in weaker cells is a major hallmark of cell competition. To look for evidence of cell competition in the posterior midgut, we therefore asked whether wild-type intestinal cells induce accelerated turnover of neighboring *M*^−/+^ cells. Depending on the experiment, here and throughout this study, we induced either the formation of wild-type (*M*^+/+^) clones in a *M*^−/+^ gut or, vice versa, we generated *M*^−/+^ clones in a (pseudo) wild-type gut. Clones were induced in recently eclosed adults by heat shock-induced, Flp-mediated mitotic recombination. In the posterior midgut, cells that are turned over are shed into the intestinal lumen following epithelial delamination. Consistent with our hypothesis, we found that in mosaic guts containing wild-type and *M*^−/+^ cells, delaminated cells (normalized to the abundance of each cell population) were more likely to be *M*^−/+^ than wild-type (Figures 1A–1C). This indicated that in competing guts, *M*^−/+^ cells have an accelerated turnover compared to wild-type cells. However, although their relatively shorter lifetime could be a consequence of their interaction with wild-type cells, it could also result from the *M*^−/+^ mutation per se. To test the latter possibility, we compared relative turnover rates of wholly wild-type and wholly *M*^−/+^ posterior midguts using a previously published genetic tool that allows pulse labeling all cells in a gut and then chasing to monitor how long they persist (Jiang et al., 2009). This revealed that, in fact, cell turnover in *M*^−/+^ guts is slower than in wild-type guts (Figures S1A–S1D). This rules out the possibility that the accelerated turnover observed in competing guts is an intrinsic property of *M*^−/+^ cells and suggests that it is induced by cell competition. Next, we compared directly the relative cell-death frequency of *M*^−/+^ cells close to wild-type cells and of *M*^−/+^ cells far away (i.e., greater than two cell diameters away; see Experimental Procedures) from wild-type cells within the same guts using the cell-death marker Sytox (Figures 1D and 1E; Figures S1E–S1F″). Importantly, the cell-death frequency of *M*^−/+^ cells was specifically increased in the proximity of wild-type cells (Figures 1D and 1E), indicating that this was a result of cell-cell interaction. This finding was further confirmed by analysis of PARP cleavage (Figures 1F–1F″), a readout of caspase activation (Williams et al., 2006) (Figures S1E–S1F″), which also indicates that (at least some) cells die of apoptosis.

We next asked which cell population was responsible for the elimination of weaker cells. The adult *Drosophila* midgut contains both actively dividing cells (i.e., ISCs) and postmitotic cells at different stages of differentiation (enteroblasts [EBs], EEs, and ECs) (Micchelli and Perrimon, 2006; Ohlstein and Spradling, 2006). Because cell competition has been observed mostly among actively dividing cells (see, however, Merino et al., 2013 and Tamori and Deng, 2013 for exceptions), we wondered whether ISCs were required for the elimination of weaker cells. We therefore devised a strategy for the efficient generation of clones of wild-type cells devoid of stem cells, exploiting the fact that Wnt signaling is required for ISC self-renewal in this tissue (Lin et al., 2008; Lee et al., 2009). We first generated wild-type stem cells in *M*^−/+^ guts by mitotic recombination and allowed them to proliferate for 4 days. We then withdrew Wnt signaling by conditionally expressing the Wnt signaling inhibitor T cell factor (TCF) dominant-negative version for 5–6 days in the stem and progenitor cell pool with the *PSwitch*^*AMP*^ Gal4 driver (an RU-486 [mifepristone]-inducible Gal-4 line that is expressed in both stem cells and EBs; Mathur et al., 2010) (Figures 1G and 1G′). Even though *PSwitch*^*AMP*^-driven GFP is not detected in all ISCs (Figure S1G), this resulted in a drastic reduction in the number of Delta-positive (Dl^+^) ISCs across the tissue (Figures S1G and S1H; note that because Dl is the most widely accepted ISC marker in the posterior midgut, we used it across this study to label ISCs). Importantly, we found that the fraction of wild-type clones surrounded by Sytox^+^ cells was not affected by the removal of ISCs (Figures 1G and 1H). Thus, differentiated cells are sufficient to trigger the elimination of weaker *M*^−/+^ cells. Altogether, these results indicate that *M*^−/+^ cells are eliminated by fitter wild-type cells in the adult *Drosophila* posterior midgut.

### Cell Competition Causes Clonal Extinction and Stem Cell Loss in Subfit Cells

**Figure 2:**
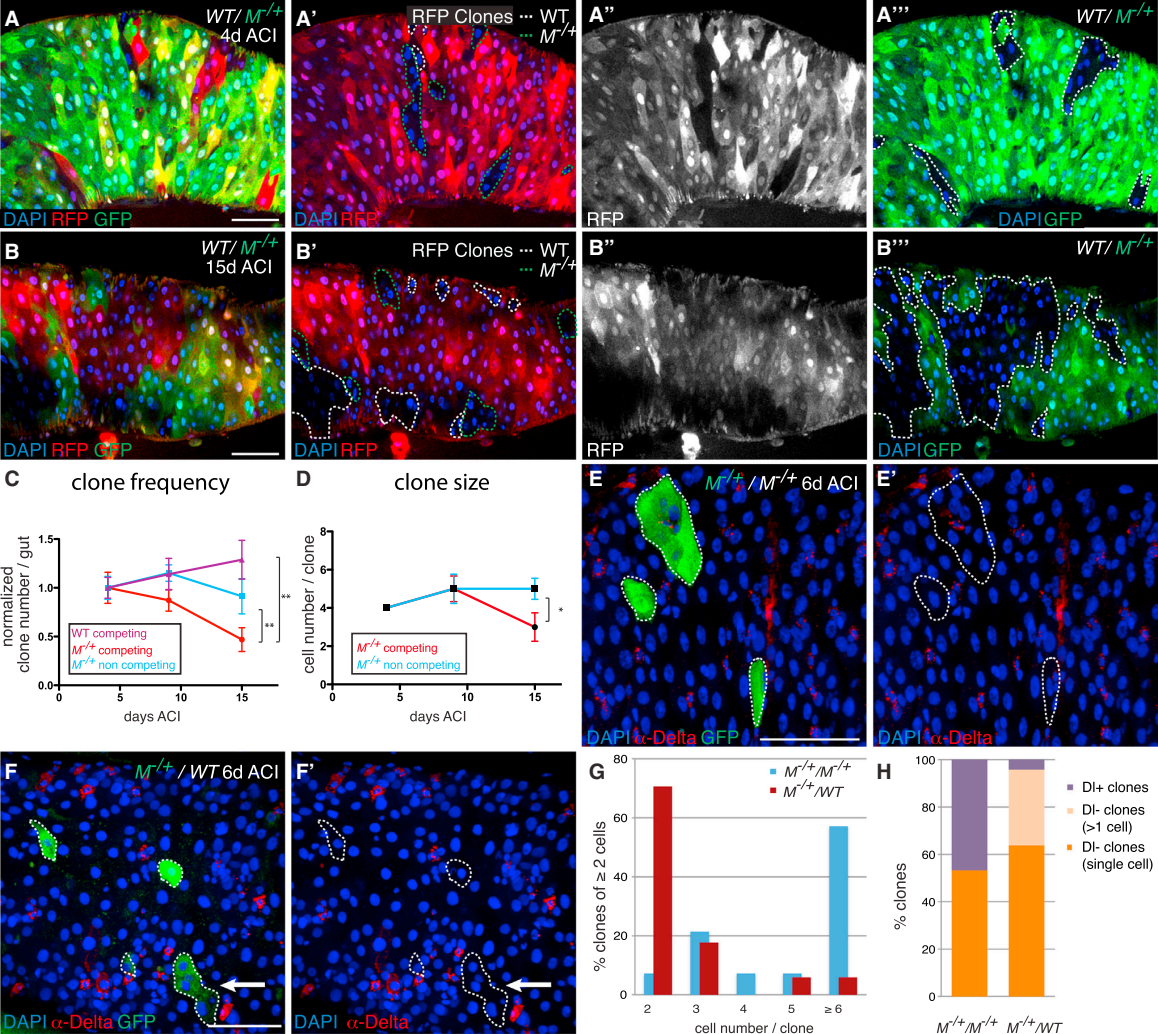
Wild-Type Cells Induce Clonal Extinction and Stem Cell Loss in *M*^−/+^ Tissues (A–D) Independently labeled *M*^−/+^ and WT clones were induced in *M*^−/+^ guts and their fate was analyzed at 4 (A–A″′, C, and D), 9 (C and D), and 15 (B–B″′, C, and D) days ACI. WT clones are GFP^−^, whereas *M*^−/+^ cells are GFP^+^. A simultaneous but independent recombination event marks clones by 0×RFP (red fluorescent protein) in an otherwise 1×RFP or 2×RFP tissue, allowing lineage tracing in *M*^−/+^ (and in WT) tissue (*hsflp; FRT40, ubiRFP/FRT40; FRT82B, ubiGFP, RpS3/FRT82B*). (C) Diagram showing the evolution of clone number (expressed as a fraction of the average clone number at 4 days) for WT clones competing in *M*^−/+^ tissue (n = 78, 78, and 101 clones at 4, 9, and 15 days ACI, respectively), competing *M*^−/+^ clones (0×RFP) from the same guts (genotype as in A) (n = 96, 73, and 45 clones at 4, 9, and 15 days ACI, respectively), and neutral *M*^−/+^ clones (0×RFP) in wholly *M*^−/+^ guts (*hsflp; FRT40, ubiRFP/FRT40; FRT82B, ubiGFP, RpS3/TM2*) (n = 142, 41, and 65 clones at 4, 9, and 15 days ACI, respectively). (D) Evolution of median clone size (genotypes and datasets are as in C). (C and D) ^∗^p < 0.05, ^∗∗^p < 0.02, Mann-Whitney test. Error bars represent SEM. (E–F′) Six-day-old GFP^+^*M*^−/+^ clones (green) in control *M*^−/+^ guts (E and E′) (*Df(1)R194, w/hsflp, actGal4, UAS CD8GFP; FRT40, tubGal80/FRT40*) or in pseudo-WT guts (F and F′) (*Df(1)R194, w/hsflp, actGal4, UAS CD8GFP; FRT40, tubGal80, P[RpL36+w+]/FRT40*) stained for Dl (red). The arrows indicate a multicellular clone devoid of Dl^+^ cells. (G) Size distribution of clones as in (E) (blue bars; n = 14 clones of two cells or more) and (F) (red bars; n = 17 clones of two cells or more). (H) Bar graphs showing the distribution of clones based on their Dl^+^ cell content for *M*^−/+^ clones in *M*^−/+^ guts (n = 30 clones) and for *M*^−/+^ clones in WT guts (n = 47 clones). Note the reduction in Dl^+^ clones for *M*^−/+^ clones in a WT background and the appearance of a large fraction of multicellular clones devoid of Dl^+^ cells. Scale bars represent 50 μm.

A second hallmark of cell competition is that it results in fitter cells taking over the tissue at the expense of less fit cells (Morata and Ripoll, 1975). Therefore, we asked whether wild-type and *M*^−/+^ cells would reciprocally affect their colonization and clone survival probabilities in this tissue. To address this, we generated *M*^−/+^ guts in which we labeled a subset of *M*^−/+^ ISCs (and their progeny) while at the same time inducing labeled wild-type ISCs (Figures 2A and 2B). We then compared clone survival frequencies between the two genotypes at 9 and 15 days after clone induction (ACI; expressed as a fraction of the average number of clones observed 4 days ACI). We also compared the survival frequency of competing *M*^−/+^ clones to that of neutral *M*^−/+^ clones (in wholly *M*^−/+^ guts). As shown in Figure 2C, the survival frequency of *M*^−/+^ clones was markedly lower than that of wild-type clones in the same guts, showing clonal disadvantage. Importantly, it was also lower than that of neutral *M*^−/+^ clones, indicating that the presence of wild-type clones negatively impacts on the survival probability of competing *M*^−/+^ cells. Consistently, this was also accompanied by a trend toward *M*^−/+^ clone attrition under competing conditions (Figure 2D).

Notably, the great majority of dying *M*^−/+^ cells in competing guts were ECs, as indicated by their large polyploid nuclei (Figures 1D–1F). This suggested that stem cells might not be affected by cell competition. However, the increased clonal extinction observed in Figure 2C indicated otherwise. To address this directly, we tracked the behavior of single competing *M*^−/+^ ISCs either in control wholly *M*^−/+^ guts (Figures 2E and 2E′) or in (pseudo) wild-type guts (Figures 2F and 2F′) and followed their fate 6 days ACI. Consistent with the results from our clonal competition experiments (Figure 2D), in this setup too, *M*^−/+^ ISCs grew into substantially bigger clones when they were in a *M*^−/+^ environment than when surrounded by wild-type cells (Figures 2E–2G). Importantly, their Dl^+^ stem cell content was also dramatically reduced in the presence of wild-type cells (Figure 2H). At 6 days ACI, whereas 46.6% of control *M*^−/+^ clones contained Dl^+^ ISCs (approaching the maximum of 50% that can be obtained by mitotic recombination in this tissue), only 4.2% of competing *M*^−/+^ clones contained Dl^+^ ISCs (Figure 2H, purple bars). Importantly, a new prominent class (31.9%) of clones was apparent, containing clones that were multicellular but had no Dl^+^ ISCs (Figures 2F and 2F′, arrows; Figure 2H, cream bar). Because the only cells that proliferate in this tissue are ISCs, this indicates that these clones must have contained ISCs and subsequently lost them. We conclude that cell competition also targets stem cells in this tissue.

### Interaction with Subfit Cells Stimulates the Expansion of Healthy Tissue via Accelerated Stem Cell Proliferation and Increased Symmetric Self-Renewal

**Figure 3:**
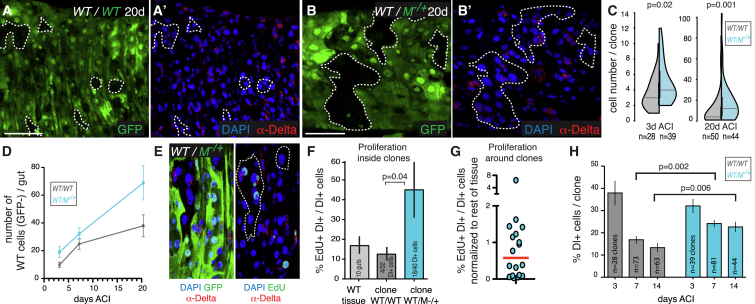
Wild-Type Cells in a *M*^−/+^ Background Increase Both Proliferation and Symmetric Self-Renewal Rates (A–B′) WT clones (marked by the absence of GFP) were generated in recently eclosed flies either in control WT (A and A′) (*hsflp/+; +/CyO; FRT82B, ubiGFP/FRT82B*; denoted as *WT/WT*) or *M*^−/+^ (B and B′) (*hsflp/+; FRT82B, ubiGFP, RpS3/FRT82B*; denoted as *WT/M*^−/+^) background, and flies were aged 3 or 20 days prior to dissection. (C) WT clone-size distributions for 3- and 20-day-old clones with genotypes as indicated. Note that the 3-day distributions do not include single-EB/EC clones (i.e., Dl^−^ single-cell clones), to remove the large number of single EBs/ECs generated by the mitotic recombination event. p: Mann-Whitney test. (D) Graph showing the average number of negatively labeled WT cells per gut (average clone number^∗^average clone size; n > 13 guts for each condition) at 3, 7, and 20 days ACI. (E and F) EdU incorporation and Dl staining in WT (not shown) or *M*^−/+^ (E) guts harboring WT clones to monitor relative proliferation rates in Dl^+^ ISCs. The bar chart in (F) shows the average proportion of Dl^+^ cells that have incorporated EdU for the indicated genotypes 7 days ACI (n > 9 guts per condition). (G) Proportion of Dl^+^ cells that have incorporated EdU in *M*^−/+^ cells surrounding WT clones, normalized to the EdU incorporation rate for *M*^−/+^ Dl^+^ cells away from clones (within the same guts). Each dot represents one gut (n = 18 guts). (H) Bar charts showing how the proportion of Dl^+^/DAPI^+^ cells changes with time in control or competing WT clones at 3, 7, and 14 days ACI. For this comparison, we only considered clones containing at least two cells and Dl^+^ single-cell clones (to filter out the large number of ECs introduced by mitotic recombination; n = number of clones; p: Mann-Whitney test). Scale bars represent 50 μm. Error bars represent SEM, except for (D), where the error was calculated as described in Supplemental Experimental Procedures. See also Figure S2.

We next considered whether, in turn, normal cells could be affected by the presence of suboptimal cells and what impact this might have on their tissue-colonization potential. Although the clonal competition assay (Figure 2C) shows that wild-type cells have a clonal advantage over *M*^−/+^ cells, this might result solely from their intrinsically faster proliferation rate, a phenomenon known as biased competition (Snippert et al., 2014). Indeed, wild-type ISCs divide significantly faster than *M*^−/+^ ISCs in this tissue (Figures S2A–S2C), and cell-autonomous differences in proliferation rate have been proposed to account entirely for the clonal expansion of wild-type clones during Minute competition in wing imaginal discs (Martín et al., 2009). To address this, we compared the behavior of control wild-type clones surrounded by wild-type cells to that of wild-type clones surrounded by *M*^−/+^ cells by lineage tracing (Figures 3A–3C) at different time points. Because in both setups the genotype of wild-type cells was identical, any change we observed between the two conditions would have to be a consequence of the interaction with *M*^−/+^ cells. Interestingly, wild-type stem cells grew into bigger clones when surrounded by *M*^−/+^ cells (Figure 3C). This was observed at early (3-day) and especially at late (20-day) time points ACI. Importantly, increased clone expansion was not a general feature of cells in *M*^−/+^ guts, because control *M*^−/+^ ISCs formed smaller clones in *M*^−/+^ guts (Figures S2D–S2F), consistent with their reduced proliferation rate (Figures S2A–S2C). Furthermore, whereas control clones grew in a manner consistent with homeostatic behavior (i.e., the average number of labeled progeny tended to plateau after initial growth, consistent with proliferation balanced by loss; de Navascués et al., 2012), competing wild-type clones expanded nonhomeostatically (Figure 3D).

The observed increase in clone size (Figure 3C) and departure from homeostasis (Figure 3D) both suggest that wild-type stem cells modify their behavior in response to cell competition. Indeed, clonal expansion could result from accelerated stem cell proliferation, increased stem cell self-renewal, or both. Interestingly, we found that relative ISC proliferation rates, measured by 5-ethynyl-2′-deoxyuridine (EdU) incorporation in Dl^+^ cells (Figures 3E and 3F), were higher for competing wild-type clones than for control wild-type clones, which divided at a rate similar to the wild-type tissue average (Figure 3F). To obtain an additional independent measure of cell-division rates, we measured clone size and stem cell composition of 3-day-old wild-type clones in control and competing conditions and estimated the minimum number of divisions that would be required to generate each clone. In line with our EdU data, the resulting estimated mean division time for wild-type cells in competing conditions was 46% faster than that of wild-type cells in control clones (16.7 hr versus 24.4 hr; p = 0.01, Mann-Whitney test). Interestingly, the proliferation increase induced by cell competition was selectively confined to wild-type ISCs, as *M*^−/+^ ISCs abutting wild-type clones did not display increased EdU incorporation (Figure 3G). This indicates that the proliferation increase observed during cell competition is different from a general proliferative response induced by localized tissue loss.

We next asked whether cell competition also modifies ISC self-renewal. Crucially, in this setup, self-renewal cannot be measured directly. Indeed, although every additional ISC within a clone represents a symmetric division event, the absolute number of symmetric divisions (i.e., of ISCs) per clone is not only influenced by the frequency of symmetric self-renewal but also by the proliferation rate, which we saw to be increased: the faster the proliferation rate, the higher the number of symmetric divisions per unit time. Importantly, however, quantifying how the ratio of Dl^+^ ISCs/total cells drops over time can inform on relative ISC self-renewal frequencies. Indeed, starting from a one-cell ISC clone, the more ISCs generated *per number of divisions* (i.e., the higher the self-renewal frequency), the more slowly the ratio will drop; conversely, the faster the proliferation rate, the faster the ratio will drop (Figure S2G). Initial values 3 days ACI were similar for wild-type cells in control and competing conditions (Figure 3H; p = 0.35 [Mann-Whitney test] between the datasets corresponding to 3 days ACI). However, remarkably, despite the faster proliferation rate, values dropped more slowly over time in competing clones (Figure 3H), indicating that cell competition increases stem cell self-renewal frequency in fitter cells. Thus, in this tissue, normal stem cells respond to the presence of weak cells by increasing both their proliferation rates and their self-renewal capacity.

### Increase in Proliferation Balanced by Biased Tissue Loss Faithfully Models the Stem Cell Dynamics of Competing Cell Populations

Having collected detailed quantitative information on the cellular parameters affected during competition, we sought to extrapolate how cell competition affects stem cell dynamics using biophysical modeling. Recent studies of the *Drosophila* posterior midgut (de Navascués et al., 2012) show that, in common with many cycling vertebrate tissues (Simons and Clevers, 2011), intestinal stem cells follow a pattern of population asymmetric self-renewal, in which stem cell loss through differentiation is perfectly compensated by the division of neighboring ISCs. A hallmark of this behavior is that the distribution of clone sizes converges onto a scaling behavior in which the chance of finding a clone larger than a multiple of the average remains constant over time (Klein and Simons, 2011; de Navascués et al., 2012). Moreover, in the epithelial arrangement of the midgut, cumulative clone-size distributions are predicted to be exponential, whereas the average size of the surviving clones grows approximately linearly with time.

**Figure 4:**
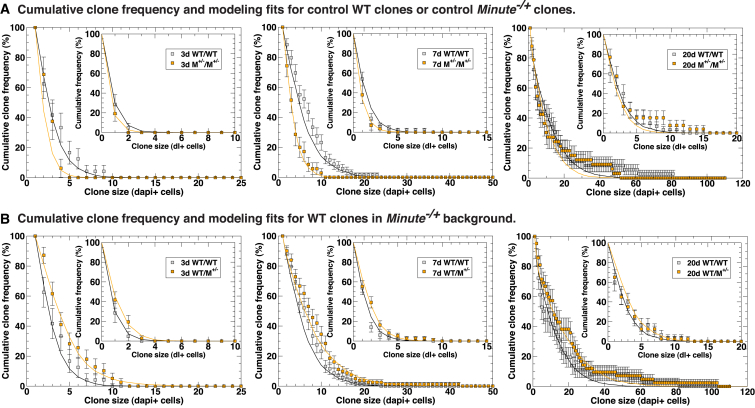
Biophysical Modeling of Stem Cell Dynamics during Cell Competition (A) Cumulative clone-size distributions and modeling fits for neutral WT (in WT) (*hsflp/+; +/CyO; FRT82B, ubiGFP/FRT82B*) and neutral *M*^−/+^ (in *M*^−/+^) clones (*hsflp/+; FRT40, ubiRFP/FRT40; FRT82B, ubiGFP, RpS3/+*). The cumulative clone frequency describes the percentage of clones that have a size larger than the given value. The panels show distributions at 3 (left), 7 (middle), and 20 (right) days ACI. The corresponding cumulative clone-size distribution for Dl^+^ cells is shown in the insets. (B) Cumulative clone-size distributions and modeling fits for competing WT clones in the *M*^−/+^ (*hsflp/+; FRT82B, ubiGFP, RpS3/FRT82B*) background and for neutral WT clones in the WT background (same as in A) as reference at 3 (left), 7 (middle), and 20 (right) days ACI. Throughout, points indicate data and continuous lines indicate the corresponding modeling fits. Error bars denote SEM, adjusted appropriately for percentages and normalized against the total clone number at each time point. To eliminate single-EB clones that are generated as part of the mitotic recombination event, single-cell clones were excluded from total clone-size distributions.

We first addressed the clonal dynamics of the control wild-type and control *M*^−/+^ epithelium (i.e., wild-type clones in a wild-type background and *M*^−/+^ clones in a *M*^−/+^ background) as a basis before turning to consider the dynamics of competing cells. Details of the modeling procedure, which mirror the methods introduced in de Navascués et al. (2012), are detailed in Experimental Procedures. Briefly, to model the dynamics of stem cells and their differentiated progeny, we considered a simple lattice model in which ISCs form a single equipotent population distributed uniformly within the epithelium. Alongside ISCs, each lattice site is associated with a fixed number of differentiating cells. To model turnover, we adopted an approach based on stochastic simulation in which a mature differentiated cell is chosen at random and removed. Following its loss, with a given probability either the ISC on the same site undergoes asymmetric cell division, giving rise to a replacement EC, or the ISC commits to EB/EC cell fate and is itself replaced by the symmetrical duplication of an ISC at a neighboring site. Alongside the overall cell-division rate (equivalently, under homeostasis, the loss rate of differentiated cells), the relative probability of symmetric versus asymmetric cell division defines in full the dynamics of the system. Using the datasets of control wild-type clones in Figure 3 and of *M*^−/+^ control clones in Figure S2, we found that the clonal fate data showed the predicted convergence to a cumulative clone-size distribution of approximately exponential form (Figure 4A). To make a quantitative fit of the model dynamics to the data, we considered the distribution of clone sizes as measured by their Dl^+^ ISC content (Figure 4A, insets). We found that the model provides a good fit to the measured cumulative clone-size distributions at all three time points in both the wild-type and the *M*^−/+^ control (Figure 4A, insets) and predicts with good approximation the total cell-number distribution at all three time points (Figure 4A, main graphs). Thus, both control wild-type (as expected) and neutral *M*^−/+^ ISCs from our experiments undergo population asymmetric self-renewal. Interestingly, from a fit to the 3- and 7-day time points, we observed that the average ISC division rate for *M*^−/+^ cells is about a factor of 2 smaller than for wild-type cells, consistent with our mitotic index data (Figures S2A–S2C).

With this platform, we then turned to consider the behavior of competing wild-type ISCs in a *M*^−/+^ background. Raw clone-size distributions revealed a qualitatively similar pattern to the wild-type controls, indicative of population asymmetry (Figure 4B). Importantly, however, consistent with the analysis from Figures 3C, 3F, and 3H, the analysis of average clone size showed a small but significant increase over wild-type controls at all three time points (Figure 4B), indicating that wild-type clones display a proliferative advantage when they are in the presence of neighboring *M*^−/+^ cells, leading to an accelerated clonal expansion of surviving clones.

We next used our model to fit the dynamics of wild-type clones in the *M*^−/+^ background, looking for a minimal adaptation of the parameters, which would capture the observed behavior. In particular, based on our observation that cell competition increases proliferation (Figure 3F) and symmetric self-renewal (Figure 3H), we introduced a moderate (25%) increase in the number of divisions. Given that our data showed a localized increase in cell loss in surrounding *M*^−/+^ cells (Figure 1), we introduced a local increase in the loss rate of neighboring *M*^−/+^ cells to exactly compensate for the increase in divisions in wild-type cells, thus maintaining the system at homeostasis. This corresponds to an ∼11-fold localized increase in loss rate compared to control *M*^−/+^ cells (see Supplemental Experimental Procedures). Importantly, the two combined adjustments allowed us to obtain a good agreement of the model with the experimental data, both when considering Dl^+^ cells only (Figure 4B, insets) and when considering the total clone size (Figure 4B, main graphs). Thus, we conclude that competing wild-type ISCs also display population asymmetry and that a modest increase in proliferation, balanced by localized biased tissue loss in surrounding *M*^−/+^ cells, can account for the clonal expansion of competing wild-type clones. This confirms that proliferation increase and biased tissue loss are the two most prominent changes that cell competition induces on adult tissue dynamics.

### Chronic JNK Activation in *M*^−/+^ Cells Promotes the Clonal Expansion of Healthy Tissue

**Figure 5:**
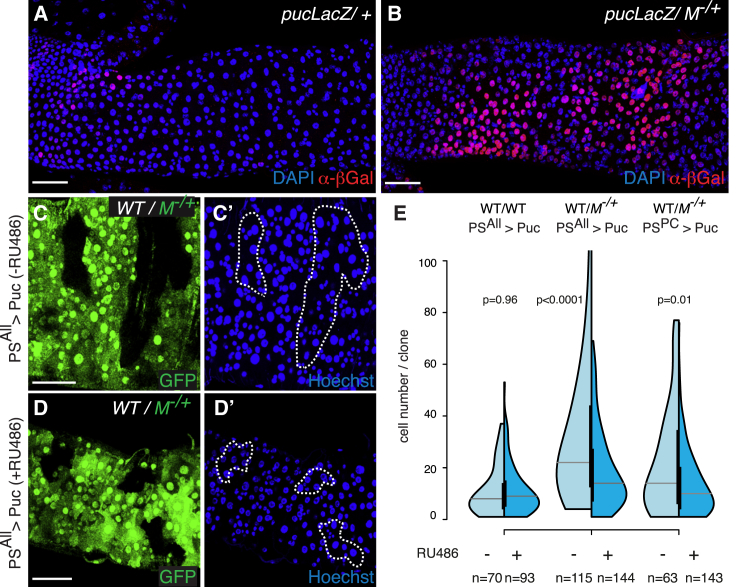
JNK Activation Promotes the Clonal Expansion of Wild-Type Cells (A and B) *puc*-LacZ expression (α-β-Gal staining) in 3-day-old WT (*FRT82B, puc*^*A251*^*/TM6B*) (A) and *M*^−/+^ (B) guts (*FRT82B, puc*^*A251*^*/FRT82B, RpS3^∗^*). (C and D) Representative images of WT clones in *M*^−/+^ guts with (D and D′) or without (C and C′) continued expression of Puc in all progenitor cells and ECs from the time of clone induction (−RU486 and +RU486, C and D, respectively; *hsflp/+; PSwitch*^*ALL*^*/UAS Puc; FRT82B, ubiGFP, RpS3/FRT82B*). (E) Analysis of clone-size distributions for WT clones either in WT guts (left) or in *M*^−/+^ guts (middle and right graphs), with (dark blue) or without (light blue) continued expression of Puc (as in C and D). Genotypes: left: *hsflp/+; PSwitch*^*ALL*^*/UAS Puc; FRT82B, ubiGFP/FRT82B*; middle: *hsflp/+; PSwitch*^*ALL*^*/UAS Puc; FRT82B, ubiGFP, RpS3/FRT82B*; right: *hsflp/+; PSwitch*^*PC*^*/UAS Puc; FRT82B, ubiGFP, RpS3/FRT82B*. p values (Mann-Whitney test) are indicated above each experiment. Scale bars represent 50 μm. See also Figure S3.

Our data show that when cells of different fitness coexist in the adult fly gut, weak cells undergo frequent cell death whereas fit cells boost their tissue-colonization ability. Cell death is known to stimulate proliferation in nearby cells in several tissues, including the fly gut, through a phenomenon known as compensatory proliferation (Fan and Bergmann, 2008; Amcheslavsky et al., 2009). In addition, inhibiting cell death has been shown to block the overproliferation of fit cells during cell competition (de la Cova et al., 2004, 2014; Li and Baker, 2007). We therefore wondered whether protecting *M*^−/+^ cells from cell competition-induced apoptosis could mitigate the overgrowth of wild-type clones. Interestingly, we found that expressing the apoptosis inhibitor Diap1, which can effectively inhibit cell death in this tissue (Figures S3A–S3D), did not reduce the overgrowth of wild-type cells, whether it was expressed in ECs (Figure S3E) or expressed in both ECs and progenitor cells (Figures S3F and S3G). This suggested that signals other than apoptosis-induced proliferation might be involved. It has been reported that several mutants whose cells are outcompeted by wild-type cells, including *M*^−/+^, display chronic activation of the JNK signaling pathway in wing imaginal discs (Tamori and Deng, 2011). We therefore asked whether in the midgut, *M*^−/+^ cells also display increased JNK signaling. Indeed, we observed higher expression of *puc*-LacZ, a transcriptional reporter of JNK activation, in *M*^−/+^ midguts compared to control guts (Figures 5A and 5B), indicating that the pathway was activated.

When the JNK pathway becomes activated in the adult midgut, such as in response to infection, inflammation, and aging, it leads to autonomous and nonautonomous proliferation (Biteau et al., 2008; Buchon et al., 2009a). We therefore asked whether JNK activation contributes to the clonal expansion of competing wild-type cells, and expressed the JNK inhibitor Puckered (Puc) throughout competing midguts (in both progenitor cells and ECs; Figures 5C and 5D). Interestingly, although JNK inhibition had no effect on the growth of control wild-type clones (Figure 5E, left), it significantly reduced the expansion of competing wild-type clones (Figure 5E, center). This was not an indirect consequence of suppressing loser cell death, as we could still observe dying *M*^−/+^ ECs neighboring wild-type clones (Figure S3H). A similar result was obtained by overexpressing Puc only in the differentiated ECs (Figure 5E, right), where JNK activation is mostly observed (Figure 5B), suggesting that JNK may fuel stem cell proliferation nonautonomously. Analogous results were obtained by expressing the dominant-negative version of JNK (JNK^DN^; data not shown). Taken together, these data suggest that in this tissue, increased cell death is not necessary to promote the clonal expansion of fitter cells. Instead, the overgrowth of healthy cells during competition depends on JNK signaling activation.

### *M*^−/+^ Cells Stimulate Wild-Type Tissue Growth via JNK-Dependent Production of Unpaired-3

**Figure 6:**
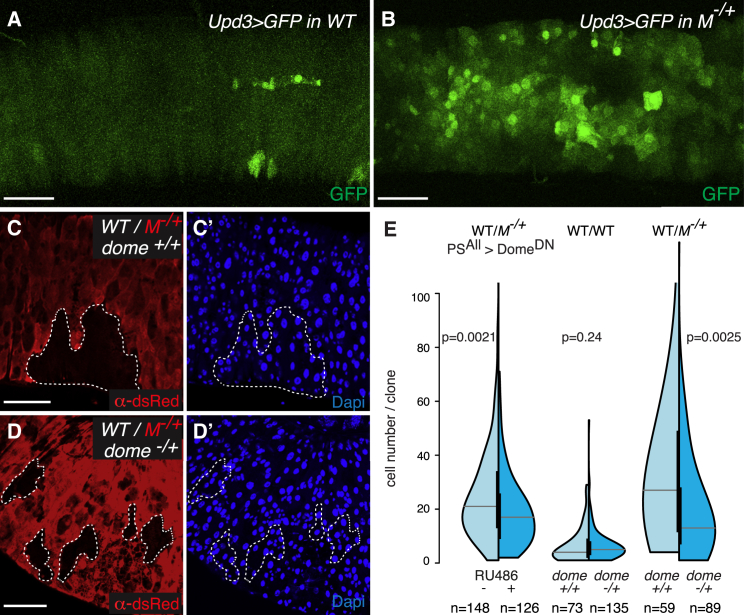
JAK-STAT Activation Fuels the Clonal Expansion of Wild-Type Cells (A and B) Expression of GFP driven by *upd3-*Gal4 in 5-day-old WT (A) (*Upd3Gal4, UASGFP/+; FRT82, tub-CD2-DsRed/+*) and *M*^−/+^ (B) guts (*Upd3Gal4, UASGFP/+; FRT82, tub-CD2-DsRed, RpS3/+*). (C and D) Representative images of WT clones either in *M*^−/+^ guts (C and C′; *hsflp/+;; FRT82B, tub-CD2-DsRed, RpS3/FRT82B*) or in *M*^−/+^ guts with reduced JAK-STAT activity by removal of one functional copy of dome (*dome*^−/+^) (D and D′; *hsflp/dome*^*G0218*^*;; FRT82B, tub-CD2-DsRed, RpS3/FRT82B*). (E) Analysis of clone-size distributions for WT clones either in WT guts or in *M*^−/+^ guts, with (dark blue) or without (light blue) reduction of JAK-STAT pathway activity by expression of Dome^DN^ (PS^All^, ±RU486) or by removal of one functional copy of dome (*dome*^−/+^). Genotypes: left: *hsflp/+; PSwitch*^*ALL*^*/UAS Dome*^*DN*^*; FRT82B, ubiGFP, RpS3/FRT82B*; middle: [WT/WT dome^+/+^] *hsflp/+;; FRT82B, tub-CD2-DsRed/FRT82B;* [WT/WT dome^−/+^] *hsflp/dome*^*G0218*^*;; FRT82B, tub-CD2-DsRed/FRT82B*; right: [WT/M^−/+^ dome^+/+^] *hsflp/+;; FRT82B, tub-CD2-DsRed, RpS3/FRT82B*; [WT/M^−/+^ dome^−/+^] *hsflp/dome*^*G0218*^*;; FRT82B, tub-CD2-DsRed, RpS3/FRT82B*. p values (Mann-Whitney test) are indicated above each experiment. Scale bars represent 50 μm. See also Figure S4.

We next addressed how JNK signaling induces the proliferation of fitter cells. A potential candidate was the secreted JAK-STAT cytokine Unpaired-3 (Upd-3), because it has been shown that JNK signaling can activate Upd-3 expression, which mediates proliferation, tissue repair, and in some cases tumor growth (Pastor-Pareja et al., 2008; Beebe et al., 2010; Buchon et al., 2009b; Wu et al., 2010; Osman et al., 2012). Indeed, we found that *M*^−/+^ guts display robust *upd-3* activation (detected using *upd3*-Gal4 and UAS-GFP; Agaisse et al., 2003; Figures 6A and 6B). Consistently, JAK-STAT activity (reported by 10×Stat-GFP; Bach et al., 2007) was higher in *M*^−/+^ guts compared to control (Figures S4A and S4B). In addition, we found that inhibiting JNK signaling in ECs (with JNK^DN^) was sufficient to restore JAK-STAT activity in *M*^−/+^ guts back to wild-type levels (Figures S4A–S4C), indicating that JAK-STAT activation is downstream of JNK signaling. To assess whether Upd-3 is the proliferative signal boosting wild-type tissue overgrowth during cell competition, we tested whether reducing JAK-STAT signaling was able to contain the clonal expansion of wild-type cells in *M*^−/+^ guts. Notably, expression of the dominant-negative Upd-3 receptor Domeless (Dome^DN^) across the posterior midgut resulted in significant size reduction of competing wild-type clones (Figure 6E, left). In addition, reducing the *dome* gene dosage was able to contain substantially the overgrowth of wild-type clones in competing conditions (Figures 6C and 6D; Figure 6E, right), whereas it had no effect on clone size in control wild-type guts (Figure 6E, center). Altogether, we conclude that Upd-3, produced by *M*^−/+^ cells downstream of chronic JNK signaling, fuels the proliferative expansion of wild-type clones during cell competition in this tissue.

## Discussion

**Figure 7:**
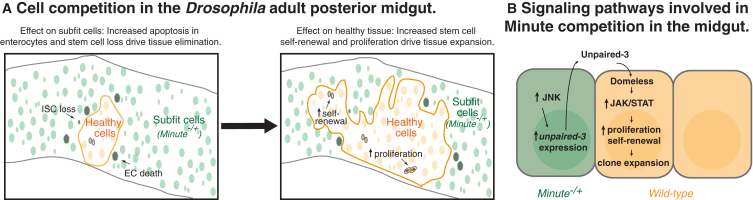
Adult Tissue Dynamics and Signaling Pathways during Minute Competition (A) Active cell competition between healthy and subfit cells in the *Drosophila* homeostatic midgut causes loss of subfit tissue. *M*^−/+^ differentiated cells are eliminated via apoptosis and *M*^−/+^ ISCs are lost, possibly by cell death or induction of differentiation (left). Conversely, the presence of unhealthy tissue promotes expansion of healthy stem cells and their progeny. ISCs increase their proliferation rate and their symmetric self-renewal, which fuels clonal expansion (right). (B) *M*^−/+^ cells drive the clonal expansion of fit cells through an inflammatory-like response. Chronic JNK signaling activation in *M*^−/+^ cells activates constitutive expression of the JAK-STAT ligand Unpaired-3. Secreted Unpaired-3, via binding to its receptor Dome, activates JAK-STAT signaling, stimulating the proliferative expansion of wild-type clones during cell competition.

Recent studies have shown that cell competition can also take place in adult tissues (Oertel et al., 2006; Villa del Campo et al., 2014). Our work has taken this notion forward and delineated quantitatively how adult stem cells and tissue population dynamics are affected by cell competition (Figure 7A). In the subfit population, differentiated cells are killed by apoptosis followed by cell delamination; stem cells are also eliminated, possibly via induction of differentiation, as we have not detected dying stem cells. In parallel, as we show, the healthy tissue expands due to an increase in stem cell proliferation and self-renewal. Indeed, biophysical modeling shows that changes in these parameters of a magnitude comparable to what we observe experimentally is sufficient to recapitulate the stem cell dynamics of wild-type tissue undergoing Minute cell competition. Interestingly, accelerated proliferation of fitter stem cells has been seen in mouse embryonic stem cells using in vitro models of cell competition (Clavería et al., 2013; Sancho et al., 2013). However, in those studies, increased stem cell self-renewal has not been observed, probably because stemness in vitro is artificially maintained by exogenous factors in the culture medium.

### Tissue Dynamics and Active Cell Competition

In many adult homeostatic tissues, stem cells stochastically differentiate or self-renew, and this leads to clonal extinction balanced by clonal expansion (Klein et al., 2007; Lopez-Garcia et al., 2010; Snippert et al., 2010; de Navascués et al., 2012). This is known as neutral drift competition, because through this process, stem cell compartments stochastically tend toward monoclonality (Klein et al., 2007; Lopez-Garcia et al., 2010; Snippert et al., 2010; de Navascués et al., 2012). It has also been shown that stem cell competition can be nonneutral (i.e., biased) when stem cells acquire a cell-autonomous advantage (Wang et al., 2009; Snippert et al., 2014). In these cases, the bias derives from *intrinsic* differences (e.g., faster proliferation) and does not rely on cell interactions. Here we show instead that in adult homeostatically maintained tissues, competitive cell interactions can act as *extrinsic* cues that actively modify stem cell behavior, and that this confers on winners an advantage (e.g., as we observe, increased proliferation rate and self-renewal) and on losers a disadvantage (e.g., as we observe, induced cell death), influencing tissue colonization. It is important to note that clones of wild-type cells that have lost proliferative capability because they are devoid of ISCs are equally able to induce death in neighboring *M*^−/+^ cells. This rules out the possibility that physical displacement due to a faster clonal expansion is the cause of cell competition in this case. This process instead, like the recent reports of cell competition in the mouse heart (Villa del Campo et al., 2014) and fly nervous system (Merino et al., 2015), likely corresponds to the adult equivalent of the cellular competition observed in developing tissues (Morata and Ripoll, 1975; de Beco et al., 2012; Vivarelli et al., 2012; Vincent et al., 2013).

### The Inflammatory Response Is Integral to Minute Cell Competition in the Midgut

Our work shows that *M*^−/+^ midguts suffer from a chronic inflammatory response, which through JNK signaling activation and the ensuing production of the JAK-STAT ligand Upd-3 promotes wild-type tissue overgrowth (Figure 7B). Thus, in this tissue, the overproliferation of winner cells stems from the increased availability of proliferative signals in the *M*^−/+^ environment. Our results suggest that wild-type cells respond more efficiently than *M*^−/+^ cells to this proliferation stimulus, and that this difference results in their preferential overgrowth, contributing to cell competition. It has long been suggested that cell competition may result from the limiting availability of growth factors, which would compromise the viability of loser cells (Raff, 1992; Moreno et al., 2002). Here we find instead that excess production of a growth factor (Upd-3) can boost cell competition by promoting preferential proliferation of fitter cells. Given that JNK and JAK-STAT are frequently activated in response to stress or deleterious mutations (e.g., Igaki et al., 2006; Ohsawa et al., 2012), it would be interesting to test whether this is a general mechanism used by loser cells to promote the overgrowth of fitter neighbors. Notably, differences in JAK-STAT signaling are sufficient to trigger cell competition (Rodrigues et al., 2012) and, consistent with this, reducing JAK-STAT signaling in wild-type cells compromises their ability to eliminate *scribble*^−/−^ losers (Schroeder et al., 2013). Thus, increased JAK-STAT signaling may in addition provide wild-type cells with a heightened fitness state and help promote the elimination of *M*^−/+^ losers.

### Adult Cell Competition and the Phenomenon of Mosaic Revertants

Ribosomal mutations are linked with many adult disorders, not just in *Drosophila* (Casad et al., 2011) but more importantly in humans, where they are associated with a number of severe pathologies, collectively known as ribosomopathies (Narla and Ebert, 2010). Given that 79 proteins make up the eukaryotic ribosome (and several more are involved in ribosomal production) and that many *Minute* mutations are dominant, the sporadic insurgence of *M*^−/+^ cells in adult tissues is likely to be one of the most common spontaneous generations of somatic mutant cells in our bodies. The elimination of these cells via cell competition is likely to play an unappreciated role in maintaining healthy adult tissues (Titen and Golic, 2008; McNamee and Brodsky, 2009; Baker, 2011).

A striking feature emerging from our results is that, in response to cell competition, normal cells can efficiently repopulate adult tissues, thus effectively replacing potentially diseased cells. This bears striking resemblance to the phenomenon of mosaic revertants, observed in a number of human skin and blood diseases (Lai-Cheong et al., 2011; Jonkman and Pasmooij, 2012). Spontaneous sporadic reversion of genetically inherited, disease-bearing mutations leads to the generation of revertant cells, which effectively repopulate tissues, at times ameliorating the condition (Lai-Cheong et al., 2011; Jonkman and Pasmooij, 2012). In some instances, the revertants’ expansion is so efficient that selective advantage has been proposed (Choate et al., 2010). Intriguingly, ichthyosis with confetti, a skin disease characterized by confetti-like appearance of revertant skin spots, is associated with a mutation in Keratin 10 (Choate et al., 2010), which, due to its nucleolar mislocalization, could affect ribosome production similar to *M*^−/+^ mutants. Thus, based on our findings, it is tentative to speculate that selective advantage in mosaic revertants could in some cases be driven by cell competition.

## Experimental Procedures

### Cell Counting

All quantifications were done manually throughout the volume of 3D reconstructions of z stacks. Clone sizes were calculated as the number of 4′,6-diamidino-2-phenylindole (DAPI)-positive cells per clone. To determine the proportion of delaminating cells in the guts (Figures 1A–1C), we analyzed confocal stacks spanning the whole gut. Cells that were detached from the epithelium (i.e., either delaminating or found in the lumen) were identified and assigned to the 2×GFP (*M*^−/+^ cells) or 1×GFP or 0×GFP (pseudo-WT cells) population according to fluorescence intensity. The proportion of *M*^−/+^ cells delaminating was determined by establishing the ratio 2×GFP delaminating/total 2×GFP cells (Figure 1C, *M*^−/+^). The proportion of (pseudo) WT cells delaminating was determined by establishing the ratio of combined 0×GFP and 1×GFP (DAPI-positive) delaminating cells/total 0×GFP and 1×GFP cells (Figure 1C, WT).

To count cells “around” clones (Figure 1E, near), we counted all cells surrounding a clone within two cell diameters in the 3D volume (this, depending on clone size, corresponds to between 10% and 30% of the GFP^+^ population, both in control and competing guts). To characterize cells not adjacent to clones (Figure 1E, far), we counted all cells minus those within and around clones. Figure 3G shows the ratios: proportion of EdU^+^ Dl^+^ cells “near” clones/proportion of EdU^+^ Dl^+^ cells “far” from clones.

### Sytox Staining

To detect dying cells, we used Sytox orange (5 mM stock in DMSO; Life Technologies; S11368) diluted 1/10,000 in 5% sucrose/water. Flies were transferred to an empty vial containing a piece of Whatman paper soaked with Sytox solution 4–5 hr prior to dissection. Because Sytox staining is not fixable, we imaged the guts within 1 day of staining.

### EdU Stainings and Quantifications

To analyze the proportion of stem cells in a replicative state, mosaic female flies were analyzed 7 days ACI. Guts were dissected out in Schneider’s medium (Sigma) and incubated for 30 min in a solution of 10 μM EdU/Schneider’s medium. After rinsing, guts were fixed and processed for immunostaining as described in Supplemental Experimental Procedures. Guts were then processed for Click-IT EdU detection according to the manufacturer’s instructions (Invitrogen; C10338). Guts were washed and mounted in Vectashield on a glass slide. The average fraction of Dl^+^ stem cells labeled with EdU following a 30-min incubation was 0.15 ± 0.06 (SD) for WT/WT guts and 0.19 ± 0.08 (SD) for WT/*M*^−/+^ guts. We processed the two genotypes in parallel and dissected 80 guts per condition in a single experiment, as stringent parameters were used to retain guts for analysis: only guts containing at least 10% EdU^+^ stem cells were retained for further analysis to minimize variability due to EdU incorporation. For the quantification of EdU^+^ stem cells *within* clones of WT cells, to avoid statistical artifacts, we further filtered out any gut in which the total number of WT ISCs per gut (summing all clones) was lower than the minimum number of cells required to encounter statistically at least one EdU^+^ stem cell (based on the proportion of EdU^+^ stem cells in that gut).

### Statistical Tests

Statistical analyses were done using Prism (GraphPad; version 5.0). p values were determined using the nonparametric Mann-Whitney test, except for Figure 1H (Fisher’s exact test). The split violin graphs (generated in R; http://www.r-project.org) represent two datasets side by side where for each sample the full distribution of values is represented as a smooth histogram. This allows for direct comparison of the range and shape of such distributions. For each dataset, the horizontal gray bar represents the median and the vertical black box indicates the 25^th^ and 75^th^ percentiles. Error bars: in Figure S2C, the averages ± SD are represented; in Figures 2C, 2D, 3F, and 3H, the averages ± SEM are represented (for Figures 2C and 2D, these values are expressed as a fraction of the values at 4 days ACI); in Figure 3D, the error (e) associated with the product (P) of the average clone size^∗^average clone frequency is calculated according to the following formula: e/P = √[(SEM of average clone size/average clone size)^2^ + (SD of average clone frequency/average clone frequency)^2^].

### Biophysical Modeling

The quantitative analysis of the clonal fate data relies upon the development of a biophysical modeling scheme, previously formulated for the study of homeostatic turnover of the *Drosophila* wild-type posterior midgut (de Navascués et al., 2012). Briefly, to model the dynamics of stem cells and their differentiated progeny, we considered a simple lattice model in which ISCs form a single equipotent population that is distributed uniformly within the epithelium. Alongside ISCs, each lattice site was associated with a fixed number of differentiating cells. To model turnover, we adopted an approach based on stochastic simulation in which a mature differentiated cell is chosen at random and removed. Following its loss, with a given probability, either the ISC on the same site undergoes asymmetric cell division, giving rise to a replacement EB, or the ISC commits to EB cell fate and is itself replaced by the symmetrical duplication of an ISC at a neighboring site. As previously shown (de Navascués et al., 2012; Klein and Simons, 2011), in such a two-dimensional model system, the clone-size distribution takes an exponential form. More precisely, the cumulative clone-size distribution, defined as the probability, *P*_*n*_(*t*), of finding a clone with a size of more than *n* cells, takes the form *P*_*n*_(*t*) = Exp[−*n*/*n*(*t*)]. Further details on how this modeling scheme was applied to competing tissues is detailed in Supplemental Experimental Procedures.

## Supplemental Information

Document S1. Supplemental Experimental Procedures and Figures S1–S4

Document S2. Article plus Supplemental Information
