# Supplementary material for: Cell Competition Modifies Adult Stem Cell and Tissue Population Dynamics in a JAK-STAT-Dependent Manner
Source: Dev Cell. 2015 Aug 10;34(3):297–309. doi: 10.1016/j.devcel.2015.06.010 (PMC4537514; doi:10.1016/j.devcel.2015.06.010)
Supplement: Document S1. Supplemental Experimental Procedures and Figures S1–S4 [file mmc1.pdf]

Developmental Cell
Supplemental Information

Cell Competition Modifies Adult Stem Cell and Tissue Population Dynamics in a JAK-STAT-Dependent Manner

Golnar Kolahgar, Saskia J.E. Suijkerbuijk, Iwo Kucinski, Enzo Z. Poirier, Sarah Mansour, Benjamin D. Simons, and Eugenia Piddini

Supplemental Figures and Legends

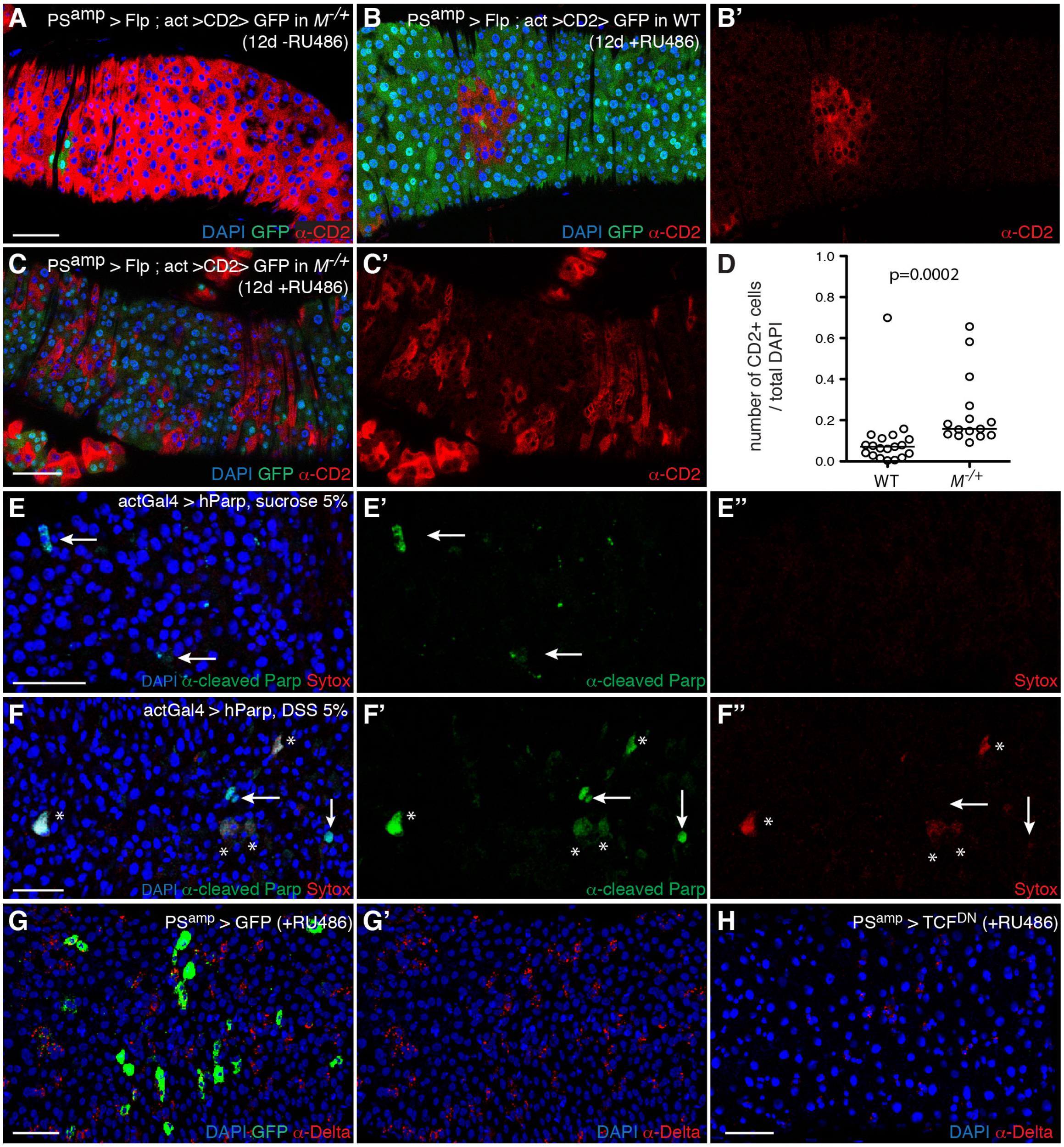

Figure S1 (related to Figure 1). Tissue turnover, cell death detection and induction of stem cell differentiation in the posterior midgut.

(A-D) Pulse-chase experiments show that posterior midgut turnover is slower in $M^{-/+}$ guts (*PS^{AMP}/UASFlp; act>CD2>Gal4, UASGFP/FRT82, RpS3*)* compared to wild-type guts (*PS^{AMP}/UASFlp; act>CD2>Gal4, UASGFP*/+). All posterior midgut cells are born CD2^{+} (red). Flp-induced excision of the CD2 cassette causes both CD2 loss and activation of Gal4, which

drives GFP expression (Jiang et al., 2009). Sustained UAS-Flp expression in progenitor cells by *PSwitch*^{AMP} and continual RU486 feeding turns all ISCs and their progeny GFP^{+} allowing to score how long old CD2^{+} cells perdure. (A) Control gut showing that in the absence of RU486, leakiness is minimal with most cells labelled by CD2 in 12-day old guts. (B, B') Representative WT gut and (C, C') $M^{-/+}$ gut after 12-day RU486 feeding; CD2 staining shows the extent of 12-day old cells still present. (D) Quantification of the proportion of 'old' CD2^{+} cells normalised to the total number of DAPI^{+} cells per gut. (n>15 guts for each genotype, p: Mann Whitney test). (E-F") Sytox incorporation and cleaved Parp staining detect cell death and apoptosis in the posterior midgut (*actGal4/UAS mCD8 Parp Venus*). (E-F") guts expressing *UAS mCD8 Parp Venus* under ubiquitous *actGal4* promoter show sporadic apoptosis by PARP cleavage (green) (E, E', arrows) and rare or no Sytox^{+} cells (red) (E, E") in sucrose fed controls, and frequent cleaved-PARP^{+} and/or Sytox^{+} cells in DSS fed guts (F-F"). Many Sytox^{+} cells are also PARP^{+} (F-F" asterisks). The occurrence of PARP^{+}/Sytox^{-} cells (F-F" arrows) suggests that PARP cleavage is an earlier and/or more sensitive readout than Sytox. (G-H) Expression of TCF^{DN} with *PSwitch*^{AMP} effectively induces ISC differentiation. (G, G') *PSwitch*^{AMP} driving the expression of GFP. GFP is detected in most but not all Dl^{+} cells after 2-day RU486 feeding (*PSwitch*^{AMP}*/UAS CD8 GFP*). (H) Induction of TCF^{DN} expression by 2-day RU486 supplementation drastically reduces the number of Dl^{+} ISCs (*PSwitch*^{AMP}*/UAS TCF*^{DN}), compared to control guts (G, G').

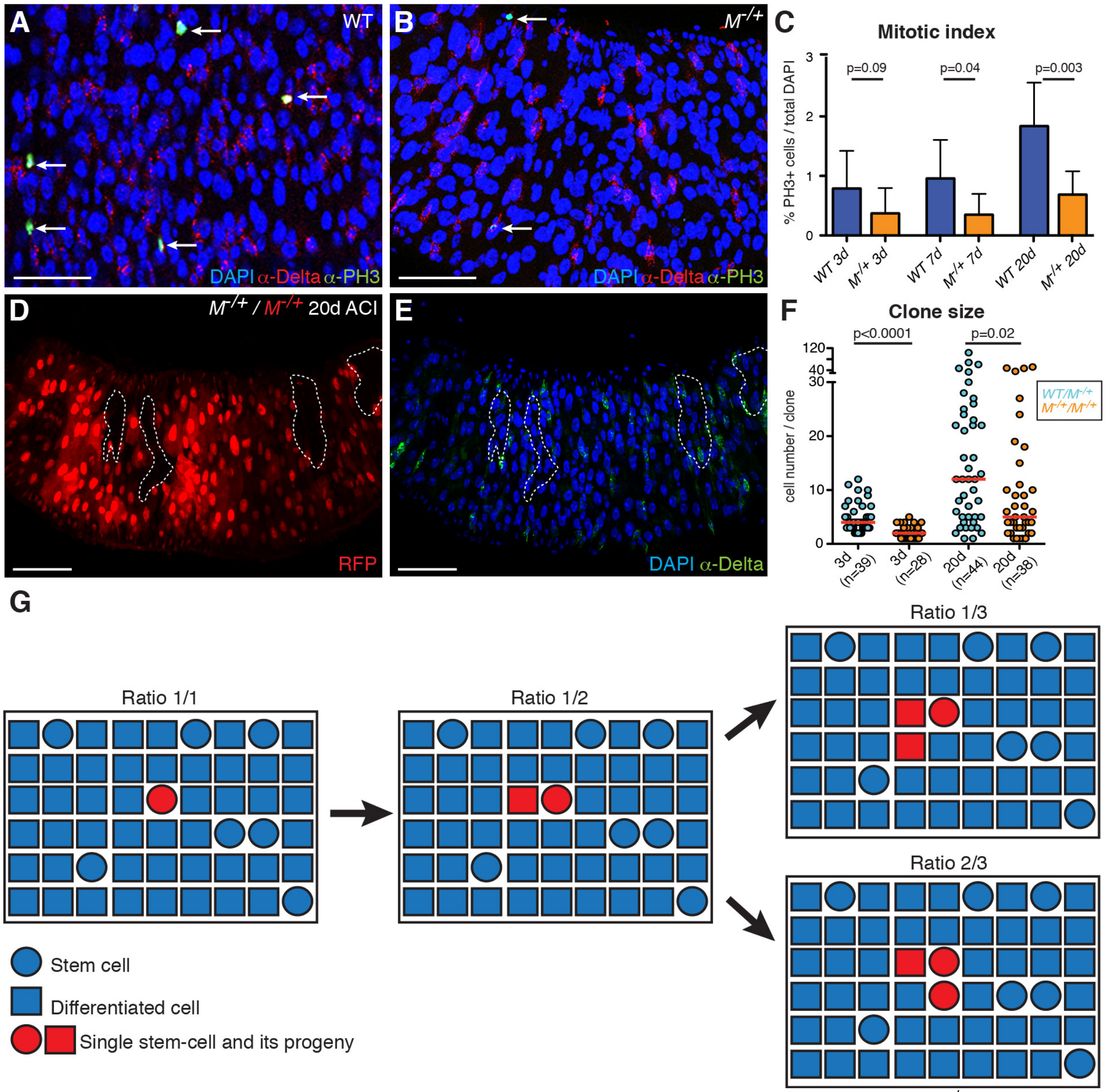

Figure S2 (related to Figure 3). Clonal growth dynamics for control *Minute*$^{-/+}$ tissue and scheme explaining how changes in clonal Delta/DAPI ratios inform on stem cell renewal rates.

(A-C) $M^{-/+}$ midguts have a reduced mitotic index. (A-B) Adult posterior midguts from 20 day old WT (A) (*yw*) or $M^{-/+}$ (B) (*FRT82B, RpS3, ubiGFP*/*TM6B*) flies, stained with the mitotic marker α-Phospho-Histone3 (α-PH3) (green, arrows) and the ISC marker α-Delta (red). (C) Quantification of the mitotic index (total PH3^{+} cells/total DAPI^{+} cells) for 3, 7, and 20 day old guts of genotype as in A and B (n>9 guts per condition; shown as average +/- SD). $M^{-/+}$ midguts display a significantly lower proliferative index compared to WT at all time points. p: Mann-Whitney test.

(D-F) Dynamics of clonal growth for *M*$^{-/+}$ clones in a *M*$^{-/+}$ background. (D-E) *M*$^{-/+}$ posterior midgut carrying control *M*$^{-/+}$ clones (marked by the absence of RFP (D) or by dotted lines (E) (*hsflp/+; FRT40 ubiRFP/FRT40; FRT82B, ubiGFP, RpS3*/+). Delta^{+} ISCs are in green (E). (F) Distribution of clone sizes shows that WT/*M*$^{-/+}$ (*hsflp/+;; FRT82B, ubiGFP, RpS3/FRT82B)* are significantly bigger that *M*$^{-/+}$/*M*$^{-/+}$ clones (genotype as in D-E) both at early and at late time points ACI (The WT/*M*$^{-/+}$ dataset is the same as in Fig. 3C). p: Mann-Whitney test (G) Schematics exemplifying how the ratio Dl^{+}/DAPI^{+} cells of an evolving single stem cell clone can inform on the frequency of its symmetric divisions.

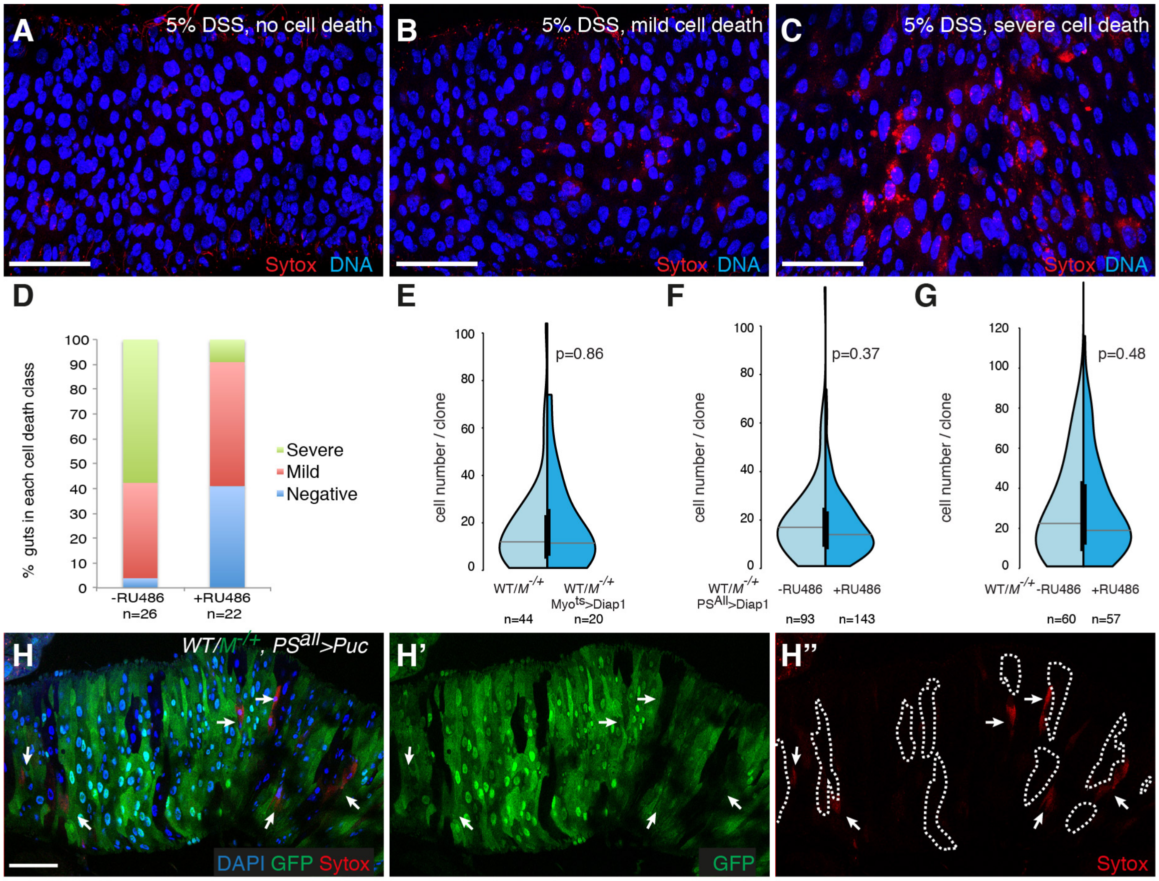

Figure S3 (related to Figure 5). Effect of Diap1 on clonal growth and effect of Puc on competition-induced apoptosis

(A-D) Diap1 expression reduces cell death in the posterior midgut. 10-12 day old flies of the genotype *hsflp/+; PSwitch^{ALL}/UAS Diap1; FRT82, ubiGFP/TM6, DfYFP,* with (+RU486) or without (-RU486) DIAP1 expression were fed 5% DSS for three days prior to Sytox incorporation. Guts were assigned to one of the three groups with negative (A), mild (B) or severe (C) cell death based on the extent of Sytox incorporation (n>22 guts for each condition). (D) shows the proportion of flies in each class for gut samples with (+RU486) or without (- RU486) DIAP1 expression. (E-G) Diap1 expression does not prevent the clonal expansion of WT cells in competing WT/$M^{-/+}$ guts. (E) Distribution of clone sizes for 20-day old WT clones in $M^{-/+}$ guts of the genotypes *hsflp/+;; FRT82B, ubiGFP, RpS3/FRT82B* (control) or *hsflp/+; MyoIAGal4, tubGal80ts/UAS Diap1; FRT82B, ubiGFP, RpS3/FRT82B* (with sustained Diap 1 expression by incubation at permissive temperature from the moment of clone induction); (F) WT clone size

distributions in guts of the genotype *hsflp/+; PSwitch^{ALL}/UAS Diap1; FRT82B, ubiGFP, RpS3/FRT82B* with or without DIAP1 expression in ECs and progenitor cells by RU486 supplementation. (G) Control experiments showing that clone size distribution is unaffected by RU486 supplementation alone (*hsflp/+;; FRT82B, ubiGFP, RpS3/FRT82B*). p: Mann Whitney test for E-G. (H-H") Puc expression in competing WT/$M^{-/+}$ guts does not abolish death of loser cells. Clones of WT cells were induced by *hs*-Flp recombination in $M^{-/+}$ guts, and Puc was continually expressed from the time of clone induction (PSwitch^{all}>Puc, +RU486, 13d ACI). Sytox positive cells (red) are still observed in $M^{-/+}$ neighboring WT cells (arrows). WT clones are marked by the absence of GFP (green) (*hsflp/+; PSwitch ^{ALL}/UAS Puc; FRT82B, ubiGFP, RpS3/FRT82B*).

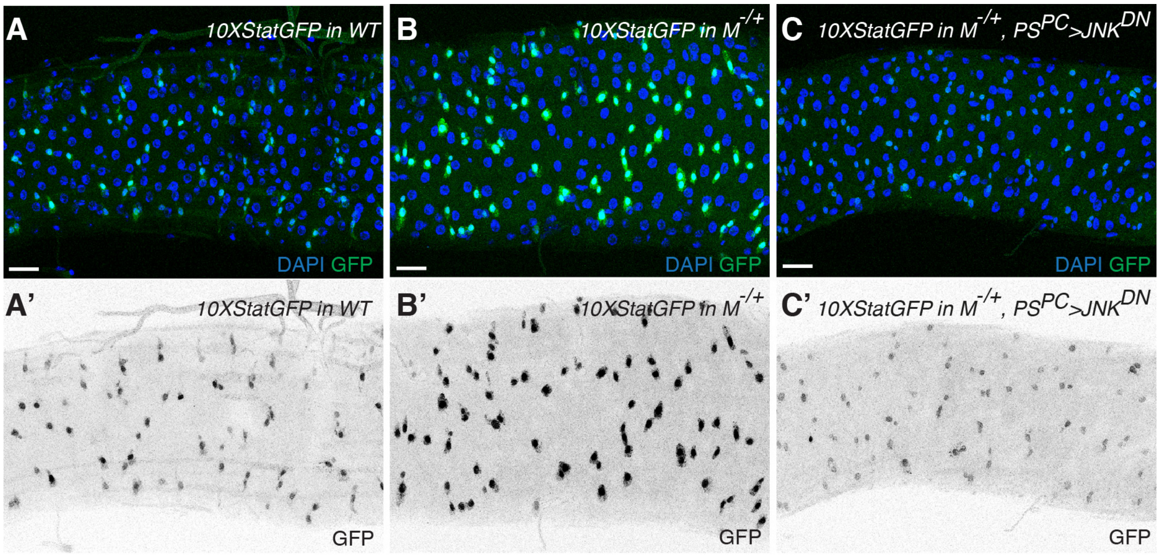

Figure S4 (related to Figure 6) JAK/STAT activation in $M^{-/+}$ cells is JNK-dependent.

(A-C) The 10XStat-GFP reporter is expressed at higher levels in $M^{-/+}$ guts (*10xStat-GFP/PSwitch^{PC}; FRT82, tubDsRed, RpS3*/+) (B-B') than in wild-type (*10xStat-GFP/PSwitch^{PC}*) guts (A-A'). Continued JNK^{DN} expression across ECs (by PSwitch^{PC} and RU486 supplementation) restores 10XStat-GFP expression to wild-type levels (*UAS Bsk^{DN}*/+; *10xStat-GFP/PSwitch^{PC}; FRT82, tubDsRed, RpS3*/+) (C-C', compare to A and B). Samples were equally aged (5 day old) and were processed and imaged in parallel. A'-C' display intensities in inverted gray levels.

Supplemental Experimental Procedures

Drosophila stocks:

yw

*Df(1)R194, w/ FM7; P[RpL36+w+]FRT40/ CyO (*N. Baker*;* the *Df(1)R194w* deficiency encompasses the RpL36 locus)

hsflp; FRT40, ubiGFP

hsflp; FRT82B/ TM6B

enGal4UASflp/ CyO; FRT82B, ubiGFP

FRT82B, ubiGFP, RpS3/TM6B (Bloomington)

FRT82B, tubDsRed

FRT82B, tubDsRed, RpS3/TM6B (recombinant generated for this study)

yw, hsflp; FRT40, M(2), arm-lacZ/ CyO

actGal4, UAS mCD8 Parp Venus (recombinant generated for this study from *UAS mCD8 Venus Parp* (D. Williams) and *actGal4/TM6B* (Bloomington)

MyoIAGal4, tubGal80^{ts} (recombinant generated for this study)

PSwitch^{AMP} (line 5961, Gal4 conditionally expressed in progenitor cells) (Mathur et al., 2010))

PSwitch^{PC} (line 5966, Gal4 conditionally expressed in enteroblasts and enterocytes cells) (Mathur et al., 2010)

PSwitch^{ALL} (this study, recombinant *PSwitch^{AMP}, PSwitch^{PC}*, used to express genes conditionally in progenitor cells and enterocytes*)*

UAS Puc^{14C} (E. Martin-Blanco)

UAS Bsk^{DN}(E. Martin-Blanco)

w; UAS Diap1/Cyo KrGal4, UASGFP; TM2/ TM6, Df YFP (P Meier and JP Vincent)

UAS dTCF^{DN} (JP Vincent)

hsflp, actGal4, UAS mCD8GFP; FRT40, tubGal80 (D. St Johnston)

hsflp, actGal4, UAS mCD8GFP/ FM7; P[RpL36+w+]FRT40 tubGal80/ CyO (recombinant and stock generated for this study)

FRT82B, RpS3/ TM6B* (Bloomington)

FRT82B, puc^{A251} *(lacZ)/ TM3* (E. Martin Blanco)

FRT40, ubiRFP (D. St Johnston)

Upd3Gal4, UAS GFP/ CyO (N. Perrimon)

10XStat-GFP (E. Bach)

w; UAS dome$^{\Delta cyt2}$*/ CyO*^{wglacZ} (J. Castelli Gair Hombria) *(aka* Dome^{DN} in the text)

w dome^{G0218} *FRT19A/ FM7c; eyFlp* (DGRC)

Drosophila genetics and stock maintenance

Flies were grown at 25°C and fed on standard fly food containing yeast. For experiments using the PSwitch system (Osterwalder et al., 2001), flies were raised on Nutrifly-GF (Dutscher Scientific UK Ltd 789234) with 200μM of mifepristone (RU486) (Sigma M8046) in EtOH 80% or an equal volume of EtOH 80% as control. For experiments using the TARGET system (McGuire et al., 2003; Figure S3E), flies were raised at 18°C and switched to 25°C after eclosion and heat shock. For Sytox feeding experiments, flies were kept at room temperature (22°C) for the duration of the feeding prior to dissection. Adults aged for guts preparation were transferred to fresh vials every 1-2 days and kept at 25°C.

Generation of mitotic clones

For clone generation in the gut, single stem-cell derived clones were generated by mitotic recombination, using the Flp/FRT system (Xu and Rubin, 1993). One to two days after eclosion, flies were heat-shocked (HS) in a water bath at 37°C and then reared at 25°C or as otherwise specified for the indicated time. Flies were aged up to a maximum of 20 days ACI, to avoid

ageing effects, which disrupt tissue homeostasis. Indeed we confirmed that at 20 day ACI, no increase in the number of ISCs (a hallmark of ageing) could be observed in $M^{-/+}$ or WT guts (data not shown). Heat-shock times were 10 minutes (for FRT82) or 30 minutes (for FRT40), except for Figure 2 A-D, where flies were heat-shocked for one hour.

Immunostaining

Guts were dissected in PBS and fixed for 20 minutes at room temperature on a shaker in a solution of PBS containing 3.7% Formaldehyde and 0.025% Triton X-100. After several washes in 0.25% Triton X-100 / PBS, guts were permeabilised for 30 minutes 1% Triton X-100 / PBS, then blocked for 30 minutes in a solution of 0.1% BSA, 0.1% Triton X-100 / PBS (blocking buffer). They were then incubated in the appropriate primary antibody diluted in blocking buffer, overnight at 4°C on a shaker**.** After several washes, guts were incubated for 2 hours at room temperature on a shaker with the appropriate secondary antibody. The following primary antibodies were used: mouse anti-Delta (DSHB, C594.9B) 1/1000, chicken anti-GFP (Abcam 13970) 1/1000, rabbit anti-phospho Histone 3 (ser10) (NEB, 9701S) 1/500, rabbit or mouse anti-cleaved human Parp (Abcam 2317, 1/25 and Abcam 110315, 1/500 respectively), rabbit anti-DsRed (Clontech 632496, 1/500), mouse anti-CD2 (Source Bioscience LS-C34521, 1/500), rabbit or chicken anti-β-Galactosidase (Cappel 55976, 1/400 and Abcam 9361, 1/1000, respectively). Secondary antibodies used were coupled to Alexa-488, Alexa 555 or Alexa-Cy5 (Molecular Probes). Nuclei were counterstained with DAPI or Hoechst. Samples were mounted in Vectashield (Vector laboratories) on a glass slide.

Confocal Acquisition and image analysis

Samples were imaged with an Olympus FV1000 Upright Confocal microscope, a Leica SP5 or a Leica SP8 confocal microscopes. All images were taken as z-stacks of 1μm sections in the

posterior midgut region immediately anterior to the hindgut (these corresponds to the regions P4 in (Marianes and Spradling, 2013); or region R5 in (Buchon et al., 2013)). Image processing, analysis and 3D reconstruction were done with Volocity (Perkin Elmer5.4.2) and Adobe Photoshop. For Figure 5A-B, in order to compare β-Galactosidase fluorescence intensity, samples were processed in parallel from eclosion of the flies to fixation, immunostaining and confocal acquisition. Similarly, guts for Figures 6A-B and S4A-C were processed in parallel. Note that the 10XStat-GFP reporter is sometimes highly upregulated in guts (including the visceral muscles) possibly due to sporadic infections. Such guts were not included in the analysis.

DSS feeding

Flies were transferred to an empty vial containing a piece of Whatman paper soaked with a solution of 5% DSS, in 5% sucrose/water, or just 5% sucrose/water. Flies were fed DSS for 2 or 3 days at 25°C, with fresh solution added daily.

Biophysical modeling

The quantitative analysis of the clonal fate data relies upon the development of a biophysical modeling scheme, which was previously formulated for the study of homeostatic turnover of the *Drosophila* wild-type posterior midgut (de Navascues et al., 2012). Briefly, following de Navascues et al., 2012, to model the dynamics of stem cells and their differentiated progeny, we considered a simple lattice model in which ISCs form a single equipotent population that are distributed uniformly within the epithelium. Alongside ISCs, each lattice site was associated with a fixed number of differentiating cells. The precise number of these differentiated cell types is fixed and discussed below. For convenience, we supposed that differentiating cells are organized as a hierarchy in which cells are lost in the order in which they mature. To model turnover we adopted an approach based on stochastic simulation in which a mature differentiated cell is

chosen at random and removed. Following its loss, with a given probability – one of only two parameters of the model, either the ISC on the same site undergoes asymmetric cell division giving rise to a replacement EB, or the ISC commits to EB cell fate and is itself replaced by the symmetrical duplication of an ISC at a neighboring site. As previously shown (de Navascues et al., 2012; Klein and Simons, 2011), in such a two-dimensional model system, the average size of surviving clones, $\langle n(t) \rangle$, is predicted to rise approximately linearly with time, while the clone size distribution takes an exponential form. More precisely, the cumulative clone size distribution, defined as the probability, $P_n(t)$, of finding a clone with a size of more than n cells, takes the form $P_n(t)=\mathrm{Exp}[-n/n(t)]$.

Modeling control wild-type clones and control $M^{-/+}$ clones.

Following induction, clones were scored at 3, 7, and 20 days post-labeling both by the number of cells that were Dl+ and by total cell number. The process of labeling by mitotic recombination in addition to labeling ISCs generates a large number of single labeled EBs, which have to be removed from the dataset for correct data analysis. Thus, we focused our analysis on the ensemble of clones with two cells or more, which must therefore be derived from the labeling of ISCs. In both wild-type and the $M^{-/+}$ control, the cumulative size distribution showed convergence to exponential form, as predicted by the model (Figure 4). In the uniform background of wild-type clones in wild-type tissue or $M^{+/-}$ clones in $M^{+/-}$ tissue, the model depends on just three adjustable parameters, the ISC division rate, the frequency of ISC loss and replacement, and the relative fraction of ISCs to differentiating cells. To *fit* the model to the data, we first placed emphasis on the number of Dl+ cells per clone as a measure of clone size (Figure 4, insets), because this depends on only the first two of the three parameters, and is more immune to statistical noise. In doing so, we also circumvented the need to consider potential adjustment in the relative abundance of ISCs and differentiated cells, which may occur as a result of ageing. Moreover, to allow for potential adjustments in the cell proliferation rate over time, we made a fit of the model at each of the three separate chase times (3, 7, 20 days) to the expected number of

rounds of cell division, i.e. we did not impose a linear relationship between the chase time and the number of rounds of division, as would be expected if the division rate stayed constant. Using a least-squares fitting procedure, we found that the data for both wild-type and the *M*$^{-/+}$ control was consistent with a loss/replacement rate of around once per five divisions with the remaining divisions leading to asymmetrical fate outcome, figures consistent with that found by (de Navascues et al., 2012). In particular, with a constant ISC division rate fixed at 0.7 per day for the wild-type control and 0.35 per day for the *Minute*$^{-/+}$ control (consistent with the experimentally measured relative difference in proliferation rate between the two genotypes; Figure S2C), we found that the model could reproduce the size distribution at both the 3 and 7 day time points (Figure 4A insets). Note that, in the two-dimensional parameter space of ISC division rate and loss/replacement frequency, a variation of around 10% in either gives a theoretical prediction that would be contained within the experimental error bars, defined as the standard error of the mean. Further, note that the inferred division rates are likely to represent a slight underestimate, as they do not include the initial round of division that generated the first labelled cell upon mitotic recombination.

With the ISC division rate and the frequency of ISC loss/replacement fixed by the Dl clone size assay, we then attempted to predict the total clone size distribution. We adjusted the relative number of ISCs as a fraction of the total population to around one in 9±1, This estimate agrees well with the direct measurements of the fraction of Dl+ cells to DAPI stained nuclei, which shows that each ISC is associated with some 8 ± 0.5 differentiating cells in both the wild-type and *M*$^{-/+}$ control (data not shown). With this adjustment we found that the model could provide a good prediction of the experimental data over all three chase times (Figure 4, main graphs). A more careful scrutiny of the clone size distributions shows that, although there is generally good agreement of the model predictions with the experimental data at the 20 day time point, there is also evidence of departure for the largest clone sizes. These apparent discrepancies likely reflect

clone fusion (which we have observed frequently in the two-clone assay (Figure 2A-C) or acute proliferative response following local damage of the epithelium.

Modeling competing wild-type clones in a $M^{-/+}$ background.

To model competing clones we looked for a minimal adaptation of the model that could capture the observed behavior. Specifically, in-line with the experimental data from Figure 3, we introduced a moderate (25%±5%) increase in the number of divisions in wild-type cells and a corresponding increase in the loss rate of $M^{-/+}$ cells on the boundary of the clone, leaving all other aspects of the cell dynamics and fate behavior unchanged. With these parameters, we found that the model could provide a quantitative prediction of the cumulative clone size distribution, reproducing both the frequencies of Dl+ cell clones and the total clone sizes (Figure 4B).

In the $M^{-/+}$ control, the cell division rate (and, therefore, the loss rate) are approximately half that of the wild-type control. Therefore, in competing conditions, the inferred increase of 25% in the loss rate of boundary $M^{-/+}$ cells translates to a ~2.5 fold increase with respect to the non competing $M^{-/+}$ control. We note that this figure represents the average loss rate increase experienced across each $M^{-/+}$ cell contained in the 9-cell units that contact wild-type cells. Given that cell competition is a short-range interaction and that wild-type clones actually contact only a small fraction of cells within each unit, it is highly likely that the burden of cell loss increase is not uniform within the unit but rather born by a small number of cells. If we suppose that, in the "lattice" of units, only an average of two cells/unit is affected by contact induced loss, these cells will experience an increase in loss rate that corresponds to a figure some 2.5*9/2=11-fold higher than control $Minute^{-/+}$ cells.

Supplemental References

Buchon, N., Osman, D., David, F.P., Fang, H.Y., Boquete, J.P., Deplancke, B., and Lemaitre, B. (2013). Morphological and molecular characterization of adult midgut compartmentalization in Drosophila. Cell Rep *3*, 1725-1738.
Marianes, A., and Spradling, A.C. (2013). Physiological and stem cell compartmentalization within the Drosophila midgut. Elife *2*, e00886.
McGuire, S.E., Le, P.T., Osborn, A.J., Matsumoto, K., and Davis, R.L. (2003). Spatiotemporal rescue of memory dysfunction in Drosophila. Science *302*, 1765-1768.
Osterwalder, T., Yoon, K.S., White, B.H., and Keshishian, H. (2001). A conditional tissue-specific transgene expression system using inducible GAL4. Proc Natl Acad Sci U S A *98*, 12596-12601.
Xu, T., and Rubin, G.M. (1993). Analysis of genetic mosaics in developing and adult Drosophila tissues. Development *117*, 1223-1237.
