# Supplementary material for: Cell Competition Modifies Adult Stem Cell and Tissue Population Dynamics in a JAK-STAT-Dependent Manner
Source: Dev Cell. 2015 Aug 10;34(3):297–309. doi: 10.1016/j.devcel.2015.06.010 (PMC4537514; doi:10.1016/j.devcel.2015.06.010)
Supplement: Document S2. Article plus Supplemental Information [file mmc2.pdf]

Developmental Cell

Article

Cell Competition Modifies Adult Stem Cell and Tissue Population Dynamics in a JAK-STAT-Dependent Manner

Graphical Abstract

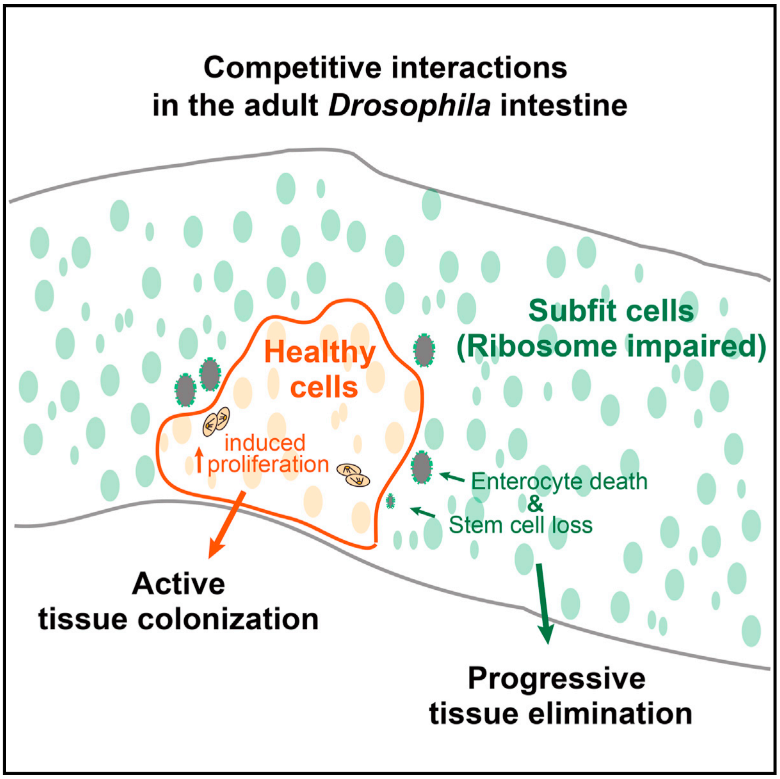

Highlights

- In the adult fly gut, wild-type cells outcompete subfit *Minute*$^{-/+}$ cells
- Both stem and differentiated *Minute*$^{-/+}$ cells are eliminated by cell competition
- Cell competition promotes proliferation and self-renewal of normal stem cells
- The growth of healthy cells is boosted by JAK-STAT signaling

Authors

Golnar Kolahgar, Saskia J.E. Suijkerbuijk, Iwo Kucinski, ..., Sarah Mansour, Benjamin D. Simons, Eugenia Piddini

Correspondence

e.piddini@gurdon.cam.ac.uk

In Brief

When cells within a tissue suffer insults that reduce their fitness, it is unclear how they are then managed. Kolahgar et al. provide evidence for active elimination of subfit cells in the adult fly gut through cell competition and show that normal cells increase tissue colonization by JAK-STAT signaling activation.

Kolahgar et al., 2015, Developmental Cell *34*, 297–309
August 10, 2015 ©2015 The Authors
http://dx.doi.org/10.1016/j.devcel.2015.06.010

Developmental Cell
Article

CellPress

Cell Competition Modifies Adult Stem Cell and Tissue Population Dynamics in a JAK-STAT-Dependent Manner

Golnar Kolahgar,[1] Saskia J.E. Suijkerbuijk,[1] Iwo Kucinski,[1] Enzo Z. Poirier,[1,2] Sarah Mansour,[1,3] Benjamin D. Simons,[1] and Eugenia Piddini[1,*]
[1]The Wellcome Trust/Cancer Research UK Gurdon Institute, University of Cambridge, Tennis Court Road, Cambridge CB2 1QN, UK
[2]Present address: Viral Populations and Pathogenesis, Institut Pasteur, CNRS UMR 3569, 28 Rue du Dr. Roux, 75724 Paris Cedex 15, France
[3]Present address: Max Planck Institute of Molecular Cell Biology and Genetics, Pfotenhauerstrasse 108, 01307 Dresden, Germany
*Correspondence: e.piddini@gurdon.cam.ac.uk
http://dx.doi.org/10.1016/j.devcel.2015.06.010
This is an open access article under the CC BY license (http://creativecommons.org/licenses/by/4.0/).

SUMMARY

Throughout their lifetime, cells may suffer insults that reduce their fitness and disrupt their function, and it is unclear how these potentially harmful cells are managed in adult tissues. We address this question using the adult *Drosophila* posterior midgut as a model of homeostatic tissue and ribosomal *Minute* mutations to reduce fitness in groups of cells. We take a quantitative approach combining lineage tracing and biophysical modeling and address how cell competition affects stem cell and tissue population dynamics. We show that healthy cells induce clonal extinction in weak tissues, targeting both stem and differentiated cells for elimination. We also find that competition induces stem cell proliferation and self-renewal in healthy tissue, promoting selective advantage and tissue colonization. Finally, we show that winner cell proliferation is fueled by the JAK-STAT ligand Unpaired-3, produced by *Minute*$^{-/+}$ cells in response to chronic JNK stress signaling.

INTRODUCTION

In adult animals, homeostatic tissues are composed of stem cells and differentiated cells that carry out specific tissue functions. The lifetime of these cells is, with few exceptions, orders of magnitude shorter than the lifetime of the individual in which they live. Thus, at any given time, adult organisms contain a proportion of cells that, either simply due to age or because of accidental damage or mutation, may be functioning suboptimally and may therefore contribute less effectively to healthy tissue function. It is normally assumed that these cells are turned over naturally when they spontaneously die. But is this really the case? How do adult tissues respond when suboptimal cells are present, and how does tissue colonization vary in the presence of heterogeneous cell communities?

It is well established that in developing tissues, cells compare their fitness with their neighbors, and less fit (“loser”) cells are eliminated through a phenomenon known as cell competition (Morata and Ripoll, 1975; de Beco et al., 2012; Vivarelli et al., 2012; Vincent et al., 2013). This likely acts as a quality control mechanism that eliminates less fit cells before they can contribute to the adult organism. Recent evidence shows that cell competition can also occur in adult tissues. For example, liver repopulation experiments show that embryonic liver cells take over adult liver tissue through a mechanism akin to cell competition (Oertel et al., 2006; Menthena et al., 2011). Similarly, cell competition has been observed between wild-type cells and cells overexpressing *myc* in the mouse heart (Villa del Campo et al., 2014). In addition, this phenomenon has been observed in some adult niche compartments (Jin et al., 2008; Issigonis et al., 2009; Rhiner et al., 2009; Bondar and Medzhitov, 2010; Marusyk et al., 2010), and a recent report suggests that it may also be taking place in adult fly tissues (Merino et al., 2015). However, how cell competition affects adult tissue dynamics and stem cell behavior has been little explored so far. In this study, we took advantage of the simplicity and genetic tractability of a well-defined model of adult homeostatic tissue, the *Drosophila* adult posterior midgut, to study the effect of cell competition on stem and differentiated cells and its consequences on tissue-level population dynamics.

The adult *Drosophila* posterior midgut in recent years has proven to be a powerful system to study adult stem cell behavior, tissue homeostasis, aging, and regeneration (Micchelli and Perrimon, 2006; Ohlstein and Spradling, 2006, 2007; Jiang and Edgar, 2012). This increasingly well characterized organ has high cellular turnover and is maintained in a way that is remarkably similar to the mammalian intestine: enterocytes (ECs) and enteroendocrine cells (EEs), which form the wall of the intestinal tube, turn over rapidly and are maintained by a supply of newly differentiated cells produced from more basally located intestinal stem cells (ISCs) (Micchelli and Perrimon, 2006; Ohlstein and Spradling, 2006, 2007; Jiang and Edgar, 2012).

As a means to reduce cellular fitness, we used mutations in ribosomal genes (known as *Minute* in *Drosophila*; Marygold et al., 2007), because they are potent inducers of cell competition in developing tissues. Indeed, it is well established that in growing imaginal discs, cells that are heterozygous mutant for *Minute* ($M^{-/+}$) are effectively eliminated by wild-type cells (Morata and Ripoll, 1975; de Beco et al., 2012; Vivarelli et al., 2012; Vincent

et al., 2013). $M^{-/+}$ mutants also have the added advantage that they are among the few loser mutations that lead to viable adults, facilitating our study.

In this work, we identify the cellular parameters that are affected by cell competition in adult tissues. We find that cell competition affects both stem cells and differentiated cells and impacts on several aspects of cell behavior, namely cell survival, proliferation, and stem cell self-renewal. We also show that the clonal expansion of fitter cells is fueled by chronic activation of Jun N-terminal kinase (JNK) and Janus kinase (JAK)-signal transducer and activator of transcription (STAT) signaling pathways in unfit $M^{-/+}$ intestinal cells.

RESULTS

Healthy Cells Induce Delamination and Apoptosis of Subfit Differentiated Enterocytes

Induction of death in weaker cells is a major hallmark of cell competition. To look for evidence of cell competition in the posterior midgut, we therefore asked whether wild-type intestinal cells induce accelerated turnover of neighboring $M^{-/+}$ cells. Depending on the experiment, here and throughout this study, we induced either the formation of wild-type ($M^{+/+}$) clones in a $M^{-/+}$ gut or, vice versa, we generated $M^{-/+}$ clones in a (pseudo) wild-type gut. Clones were induced in recently eclosed adults by heat shock-induced, Flp-mediated mitotic recombination. In the posterior midgut, cells that are turned over are shed into the intestinal lumen following epithelial delamination. Consistent with our hypothesis, we found that in mosaic guts containing wild-type and $M^{-/+}$ cells, delaminated cells (normalized to the abundance of each cell population) were more likely to be $M^{-/+}$ than wild-type (Figures 1A–1C). This indicated that in competing guts, $M^{-/+}$ cells have an accelerated turnover compared to wild-type cells. However, although their relatively shorter lifetime could be a consequence of their interaction with wild-type cells, it could also result from the $M^{-/+}$ mutation per se. To test the latter possibility, we compared relative turnover rates of wholly wild-type and wholly $M^{-/+}$ posterior midguts using a previously published genetic tool that allows pulse labeling all cells in a gut and then chasing to monitor how long they persist (Jiang et al., 2009). This revealed that, in fact, cell turnover in $M^{-/+}$ guts is slower than in wild-type guts (Figures S1A–S1D). This rules out the possibility that the accelerated turnover observed in competing guts is an intrinsic property of $M^{-/+}$ cells and suggests that it is induced by cell competition. Next, we compared directly the relative cell-death frequency of $M^{-/+}$ cells close to wild-type cells and of $M^{-/+}$ cells far away (i.e., greater than two cell diameters away; see Experimental Procedures) from wild-type cells within the same guts using the cell-death marker Sytox (Figures 1D and 1E; Figures S1E–S1F″). Importantly, the cell-death frequency of $M^{-/+}$ cells was specifically increased in the proximity of wild-type cells (Figures 1D and 1E), indicating that this was a result of cell-cell interaction. This finding was further confirmed by analysis of PARP cleavage (Figures 1F–1F″), a readout of caspase activation (Williams et al., 2006) (Figures S1E–S1F″), which also indicates that (at least some) cells die of apoptosis.

We next asked which cell population was responsible for the elimination of weaker cells. The adult *Drosophila* midgut contains both actively dividing cells (i.e., ISCs) and postmitotic cells at different stages of differentiation (enteroblasts [EBs], EEs, and ECs) (Micchelli and Perrimon, 2006; Ohlstein and Spradling, 2006). Because cell competition has been observed mostly among actively dividing cells (see, however, Merino et al., 2013 and Tamori and Deng, 2013 for exceptions), we wondered whether ISCs were required for the elimination of weaker cells. We therefore devised a strategy for the efficient generation of clones of wild-type cells devoid of stem cells, exploiting the fact that Wnt signaling is required for ISC self-renewal in this tissue (Lin et al., 2008; Lee et al., 2009). We first generated wild-type stem cells in $M^{-/+}$ guts by mitotic recombination and allowed them to proliferate for 4 days. We then withdrew Wnt signaling by conditionally expressing the Wnt signaling inhibitor T cell factor (TCF) dominant-negative version for 5–6 days in the stem and progenitor cell pool with the *PSwitch*^{AMP} Gal4 driver (an RU-486 [mifepristone]-inducible Gal-4 line that is expressed in both stem cells and EBs; Mathur et al., 2010) (Figures 1G and 1G′). Even though *PSwitch*^{AMP}-driven GFP is not detected in all ISCs (Figure S1G), this resulted in a drastic reduction in the number of Delta-positive (Dl^{+}) ISCs across the tissue (Figures S1G and S1H; note that because Dl is the most widely accepted ISC marker in the posterior midgut, we used it across this study to label ISCs). Importantly, we found that the fraction of wild-type clones surrounded by $Sytox^{+}$ cells was not affected by the removal of ISCs (Figures 1G and 1H). Thus, differentiated cells are sufficient to trigger the elimination of weaker $M^{-/+}$ cells. Altogether, these results indicate that $M^{-/+}$ cells are eliminated by fitter wild-type cells in the adult *Drosophila* posterior midgut.

Cell Competition Causes Clonal Extinction and Stem Cell Loss in Subfit Cells

A second hallmark of cell competition is that it results in fitter cells taking over the tissue at the expense of less fit cells (Morata and Ripoll, 1975). Therefore, we asked whether wild-type and $M^{-/+}$ cells would reciprocally affect their colonization and clone survival probabilities in this tissue. To address this, we generated $M^{-/+}$ guts in which we labeled a subset of $M^{-/+}$ ISCs (and their progeny) while at the same time inducing labeled wild-type ISCs (Figures 2A and 2B). We then compared clone survival frequencies between the two genotypes at 9 and 15 days after clone induction (ACI; expressed as a fraction of the average number of clones observed 4 days ACI). We also compared the survival frequency of competing $M^{-/+}$ clones to that of neutral $M^{-/+}$ clones (in wholly $M^{-/+}$ guts). As shown in Figure 2C, the survival frequency of $M^{-/+}$ clones was markedly lower than that of wild-type clones in the same guts, showing clonal disadvantage. Importantly, it was also lower than that of neutral $M^{-/+}$ clones, indicating that the presence of wild-type clones negatively impacts on the survival probability of competing $M^{-/+}$ cells. Consistently, this was also accompanied by a trend toward $M^{-/+}$ clone attrition under competing conditions (Figure 2D).

Notably, the great majority of dying $M^{-/+}$ cells in competing guts were ECs, as indicated by their large polyploid nuclei (Figures 1D–1F). This suggested that stem cells might not be affected by cell competition. However, the increased clonal extinction observed in Figure 2C indicated otherwise. To address this directly, we tracked the behavior of single competing $M^{-/+}$ ISCs either in control wholly $M^{-/+}$ guts (Figures

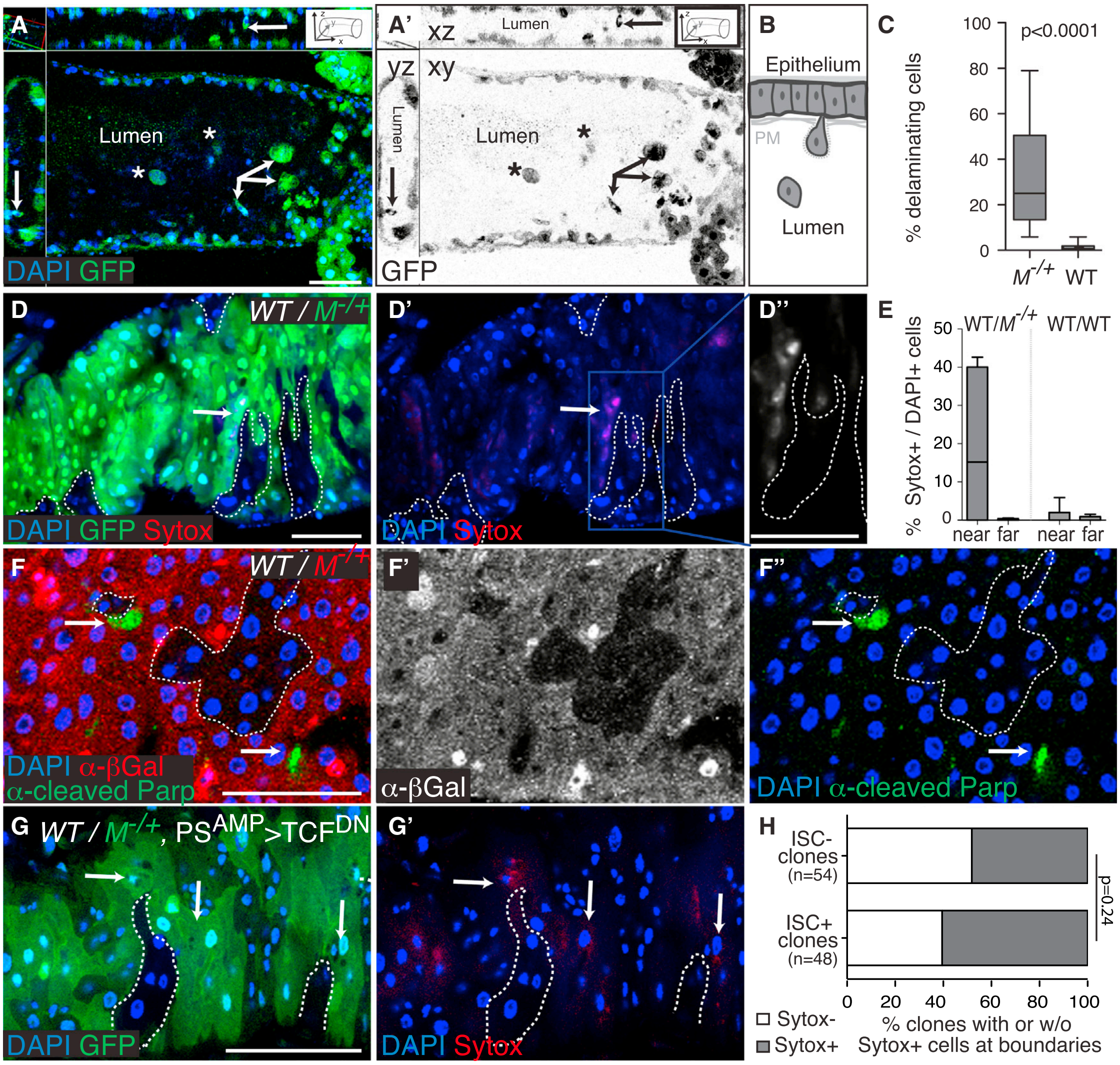

Figure 1. Wild-Type Cells Induce Accelerated Turnover of $M^{-/+}$ Intestinal Cells

(A and A′) Posterior midgut cross-sections along the xy, xz, and yz axes showing the epithelial walls and intestinal lumen. $M^{-/+}$ clones (2×GFP, arrows) were generated in a pseudo-WT background by *hs*-Flp recombination (WT; 1×GFP, asterisks) (genotype: *Df(1)R194, w/hsflp; FRT40, ubiGFP/P[RpL36+w+], FRT40*). Delaminated cells seen in the lumen were assigned to the WT or $M^{-/+}$ population based on GFP intensity, shown in grayscale (A′).

(B) Cartoon depicting epithelial cells delaminating from the epithelium into the gut lumen. PM, peritrophic matrix.

(C) Box plots displaying the percentage of delaminating cells throughout the 3D volume of the gut lumen for WT or $M^{-/+}$ cells in guts as in (A) (n = 21 guts; p: Mann-Whitney test).

(D–D″) $M^{-/+}$ posterior midgut harboring *hs*-Flp-induced WT clones (GFP-negative) stained with the cell-death label Sytox (red in D and D′ [arrows]; gray in D″) (*hsflp/+; FRT82B, ubiGFP, RpS3/FRT82B*).

(E) Box plots displaying the percentage of $M^{-/+}$ Sytox$^+$ cells among the cells near (less than two rows of cells) or far (more than two rows of cells) from WT clones (n = 11 guts; genotype as in D) or the percentage of Sytox$^+$ cells in control WT cells near or far from WT clones (n = 12 guts; genotype: *hsflp/+; +/CyO; FRT82B, ubiGFP/FRT82B*) (p = 0.03 for *WT/*$M^{-/+}$ and p = 0.52 for *WT/WT*, Mann-Whitney test).

(F–F″) Cleaved PARP detection (green; arrows) reveals frequent apoptosis in $M^{-/+}$ cells bordering WT cells (β-Gal-negative) (*hsflp/+; FRT40, M(2), arm-lacZ/FRT40; actGal4, UAS mCD8 Parp Venus/+*).

(G and H) $M^{-/+}$ posterior midgut harboring *hs*-Flp-induced WT clones (G and G′). Clones were left to grow for 4 days ACI and then TCF^{DN} was expressed continually for 5–6 days across progenitor cells (*PSwitch*^{amp}*>TCF*^{DN}, +RU486) to induce ISC differentiation (*hsflp/+; PSwitch*^{AMP}*/UAS TCF*^{DN}*; FRT82B, ubiGFP, RpS3/FRT82B*). Clones were scored for the presence or absence of ISCs (scoring small DAPI$^+$ cells as a proxy for ISCs) and further analyzed for the presence of Sytox$^+$ $M^{-/+}$ cells at clone boundaries (arrows in G and G′) (n = 52 ISC$^-$ clones; n = 48 ISC$^+$ clones). Differences in the distribution of clones between these two categories were not significantly different (p = 0.24, Fisher's exact test) (H).

Scale bars represent 50 μm. Genotypes are indicated in parentheses throughout the figure legends. Clones (defined in the z volume) are marked with dotted lines throughout the figures. See also Figure S1.

CellPress

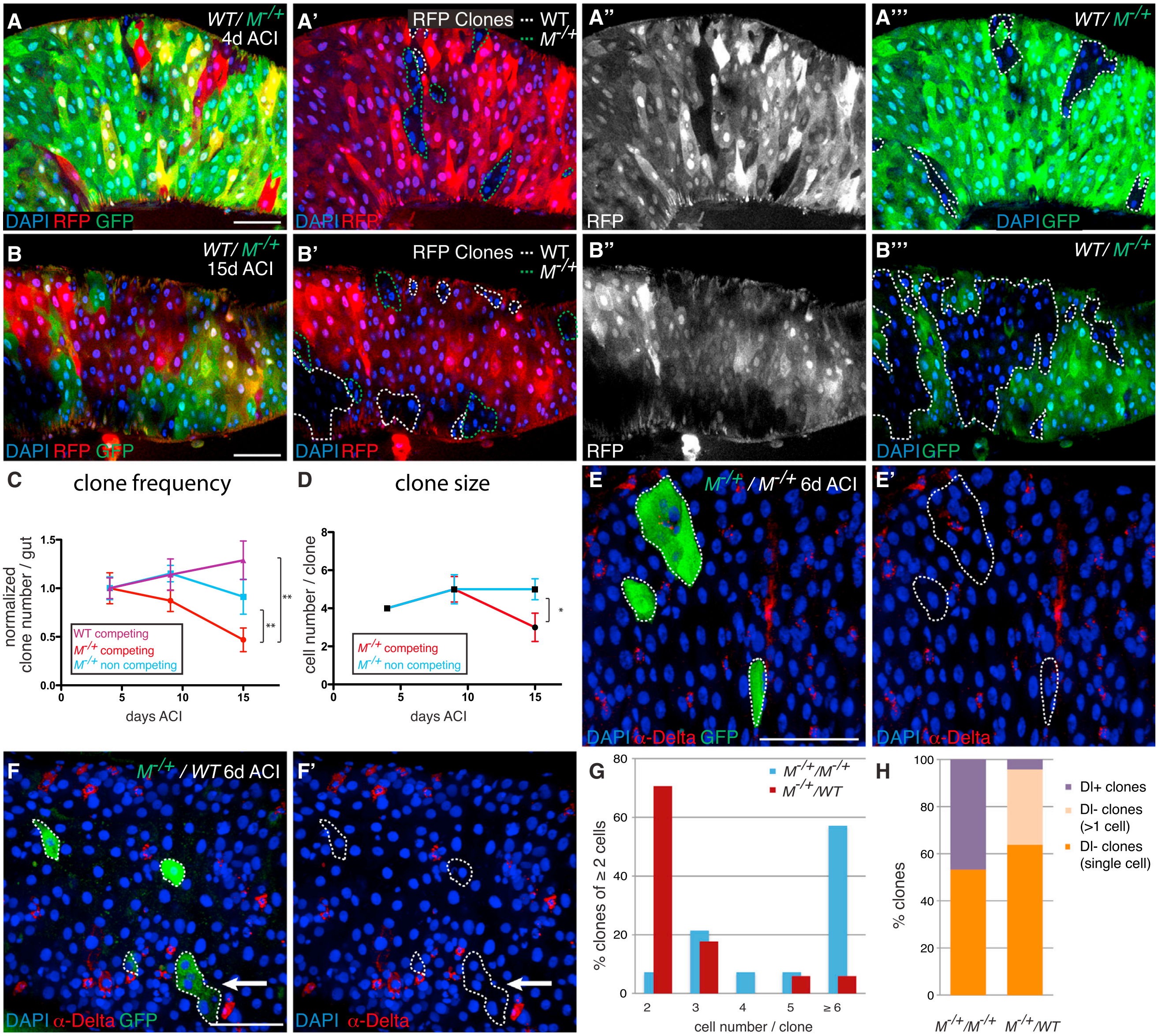

Figure 2. Wild-Type Cells Induce Clonal Extinction and Stem Cell Loss in $M^{-/+}$ Tissues

(A–D) Independently labeled $M^{-/+}$ and WT clones were induced in $M^{-/+}$ guts and their fate was analyzed at 4 (A–A‴, C, and D), 9 (C and D), and 15 (B–B‴, C, and D) days ACI. WT clones are GFP^{-}, whereas $M^{-/+}$ cells are GFP^{+}. A simultaneous but independent recombination event marks clones by 0×RFP (red fluorescent protein) in an otherwise 1×RFP or 2×RFP tissue, allowing lineage tracing in $M^{-/+}$ (and in WT) tissue (*hsflp; FRT40, ubiRFP/FRT40; FRT82B, ubiGFP, RpS3/FRT82B*).

(C) Diagram showing the evolution of clone number (expressed as a fraction of the average clone number at 4 days) for WT clones competing in $M^{-/+}$ tissue (n = 78, 78, and 101 clones at 4, 9, and 15 days ACI, respectively), competing $M^{-/+}$ clones (0×RFP) from the same guts (genotype as in A) (n = 96, 73, and 45 clones at 4, 9, and 15 days ACI, respectively), and neutral $M^{-/+}$ clones (0×RFP) in wholly $M^{-/+}$ guts (*hsflp; FRT40, ubiRFP/FRT40; FRT82B, ubiGFP, RpS3/TM2*) (n = 142, 41, and 65 clones at 4, 9, and 15 days ACI, respectively).

(D) Evolution of median clone size (genotypes and datasets are as in C).

(C and D) *$p < 0.05$, **$p < 0.02$, Mann-Whitney test. Error bars represent SEM.

(E–F′) Six-day-old GFP^{+} $M^{-/+}$ clones (green) in control $M^{-/+}$ guts (E and E′) (*Df(1)R194, w/hsflp, actGal4, UAS CD8GFP; FRT40, tubGal80/FRT40*) or in pseudo-WT guts (F and F′) (*Df(1)R194, w/hsflp, actGal4, UAS CD8GFP; FRT40, tubGal80, P[RpL36+w+]/FRT40*) stained for Dl (red). The arrows indicate a multicellular clone devoid of Dl^{+} cells.

(G) Size distribution of clones as in (E) (blue bars; n = 14 clones of two cells or more) and (F) (red bars; n = 17 clones of two cells or more).

(H) Bar graphs showing the distribution of clones based on their Dl^{+} cell content for $M^{-/+}$ clones in $M^{-/+}$ guts (n = 30 clones) and for $M^{-/+}$ clones in WT guts (n = 47 clones). Note the reduction in Dl^{+} clones for $M^{-/+}$ clones in a WT background and the appearance of a large fraction of multicellular clones devoid of Dl^{+} cells.

Scale bars represent 50 μm.

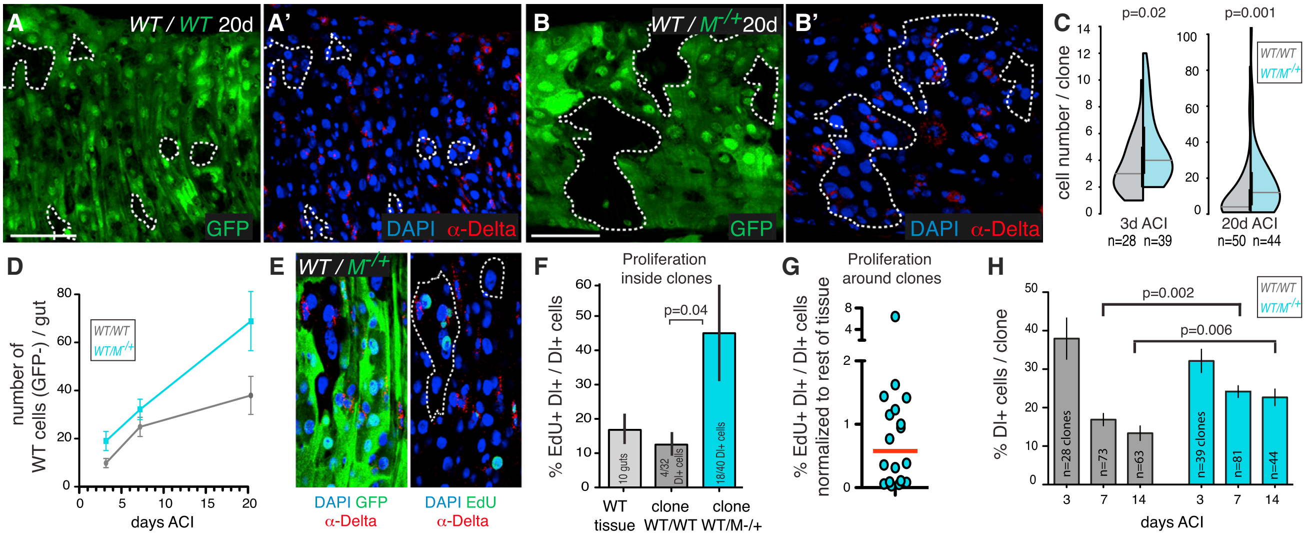

Figure 3. Wild-Type Cells in a $M^{-/+}$ Background Increase Both Proliferation and Symmetric Self-Renewal Rates

(A–B′) WT clones (marked by the absence of GFP) were generated in recently eclosed flies either in control WT (A and A′) (*hsflp/+; +/CyO; FRT82B, ubiGFP/FRT82B*; denoted as *WT/WT*) or $M^{-/+}$ (B and B′) (*hsflp/+; FRT82B, ubiGFP, RpS3/FRT82B*; denoted as *WT/*$M^{-/+}$) background, and flies were aged 3 or 20 days prior to dissection.

(C) WT clone-size distributions for 3- and 20-day-old clones with genotypes as indicated. Note that the 3-day distributions do not include single-EB/EC clones (i.e., Dl^- single-cell clones), to remove the large number of single EBs/ECs generated by the mitotic recombination event. p: Mann-Whitney test.

(D) Graph showing the average number of negatively labeled WT cells per gut (average clone number*average clone size; n > 13 guts for each condition) at 3, 7, and 20 days ACI.

(E and F) EdU incorporation and Dl staining in WT (not shown) or $M^{-/+}$ (E) guts harboring WT clones to monitor relative proliferation rates in Dl^+ ISCs. The bar chart in (F) shows the average proportion of Dl^+ cells that have incorporated EdU for the indicated genotypes 7 days ACI (n > 9 guts per condition).

(G) Proportion of Dl^+ cells that have incorporated EdU in $M^{-/+}$ cells surrounding WT clones, normalized to the EdU incorporation rate for $M^{-/+}$ Dl^+ cells away from clones (within the same guts). Each dot represents one gut (n = 18 guts).

(H) Bar charts showing how the proportion of Dl^+/$DAPI^+$ cells changes with time in control or competing WT clones at 3, 7, and 14 days ACI. For this comparison, we only considered clones containing at least two cells and Dl^+ single-cell clones (to filter out the large number of ECs introduced by mitotic recombination; n = number of clones; p: Mann-Whitney test).

Scale bars represent 50 μm. Error bars represent SEM, except for (D), where the error was calculated as described in Supplemental Experimental Procedures. See also Figure S2.

2E and 2E′) or in (pseudo) wild-type guts (Figures 2F and 2F′) and followed their fate 6 days ACI. Consistent with the results from our clonal competition experiments (Figure 2D), in this setup too, $M^{-/+}$ ISCs grew into substantially bigger clones when they were in a $M^{-/+}$ environment than when surrounded by wild-type cells (Figures 2E–2G). Importantly, their Dl^+ stem cell content was also dramatically reduced in the presence of wild-type cells (Figure 2H). At 6 days ACI, whereas 46.6% of control $M^{-/+}$ clones contained Dl^+ ISCs (approaching the maximum of 50% that can be obtained by mitotic recombination in this tissue), only 4.2% of competing $M^{-/+}$ clones contained Dl^+ ISCs (Figure 2H, purple bars). Importantly, a new prominent class (31.9%) of clones was apparent, containing clones that were multicellular but had no Dl^+ ISCs (Figures 2F and 2F′, arrows; Figure 2H, cream bar). Because the only cells that proliferate in this tissue are ISCs, this indicates that these clones must have contained ISCs and subsequently lost them. We conclude that cell competition also targets stem cells in this tissue.

Interaction with Subfit Cells Stimulates the Expansion of Healthy Tissue via Accelerated Stem Cell Proliferation and Increased Symmetric Self-Renewal

We next considered whether, in turn, normal cells could be affected by the presence of suboptimal cells and what impact this might have on their tissue-colonization potential. Although the clonal competition assay (Figure 2C) shows that wild-type cells have a clonal advantage over $M^{-/+}$ cells, this might result solely from their intrinsically faster proliferation rate, a phenomenon known as biased competition (Snippert et al., 2014). Indeed, wild-type ISCs divide significantly faster than $M^{-/+}$ ISCs in this tissue (Figures S2A–S2C), and cell-autonomous differences in proliferation rate have been proposed to account entirely for the clonal expansion of wild-type clones during Minute competition in wing imaginal discs (Martín et al., 2009). To address this, we compared the behavior of control wild-type clones surrounded by wild-type cells to that of wild-type clones surrounded by $M^{-/+}$ cells by lineage tracing (Figures 3A–3C) at different time points. Because in both setups the genotype of wild-type cells was identical, any change we observed between the two conditions would have to be a consequence of the interaction with $M^{-/+}$ cells. Interestingly, wild-type stem cells grew into bigger clones when surrounded by $M^{-/+}$ cells (Figure 3C). This was observed at early (3-day) and especially at late (20-day) time points ACI. Importantly, increased clone expansion was not a general feature of cells in $M^{-/+}$ guts, because control $M^{-/+}$ ISCs formed smaller clones in $M^{-/+}$ guts (Figures S2D–S2F), consistent with their reduced proliferation rate (Figures S2A–S2C). Furthermore, whereas control clones grew in a

manner consistent with homeostatic behavior (i.e., the average number of labeled progeny tended to plateau after initial growth, consistent with proliferation balanced by loss; de Navascués et al., 2012), competing wild-type clones expanded nonhomeostatically (Figure 3D).

The observed increase in clone size (Figure 3C) and departure from homeostasis (Figure 3D) both suggest that wild-type stem cells modify their behavior in response to cell competition. Indeed, clonal expansion could result from accelerated stem cell proliferation, increased stem cell self-renewal, or both. Interestingly, we found that relative ISC proliferation rates, measured by 5-ethynyl-2′-deoxyuridine (EdU) incorporation in Dl^+ cells (Figures 3E and 3F), were higher for competing wild-type clones than for control wild-type clones, which divided at a rate similar to the wild-type tissue average (Figure 3F). To obtain an additional independent measure of cell-division rates, we measured clone size and stem cell composition of 3-day-old wild-type clones in control and competing conditions and estimated the minimum number of divisions that would be required to generate each clone. In line with our EdU data, the resulting estimated mean division time for wild-type cells in competing conditions was 46% faster than that of wild-type cells in control clones (16.7 hr versus 24.4 hr; $p = 0.01$, Mann-Whitney test). Interestingly, the proliferation increase induced by cell competition was selectively confined to wild-type ISCs, as $M^{-/+}$ ISCs abutting wild-type clones did not display increased EdU incorporation (Figure 3G). This indicates that the proliferation increase observed during cell competition is different from a general proliferative response induced by localized tissue loss.

We next asked whether cell competition also modifies ISC self-renewal. Crucially, in this setup, self-renewal cannot be measured directly. Indeed, although every additional ISC within a clone represents a symmetric division event, the absolute number of symmetric divisions (i.e., of ISCs) per clone is not only influenced by the frequency of symmetric self-renewal but also by the proliferation rate, which we saw to be increased: the faster the proliferation rate, the higher the number of symmetric divisions per unit time. Importantly, however, quantifying how the ratio of Dl^+ ISCs/total cells drops over time can inform on relative ISC self-renewal frequencies. Indeed, starting from a one-cell ISC clone, the more ISCs generated *per number of divisions* (i.e., the higher the self-renewal frequency), the more slowly the ratio will drop; conversely, the faster the proliferation rate, the faster the ratio will drop (Figure S2G). Initial values 3 days ACI were similar for wild-type cells in control and competing conditions (Figure 3H; $p = 0.35$ [Mann-Whitney test] between the datasets corresponding to 3 days ACI). However, remarkably, despite the faster proliferation rate, values dropped more slowly over time in competing clones (Figure 3H), indicating that cell competition increases stem cell self-renewal frequency in fitter cells. Thus, in this tissue, normal stem cells respond to the presence of weak cells by increasing both their proliferation rates and their self-renewal capacity.

Increase in Proliferation Balanced by Biased Tissue Loss Faithfully Models the Stem Cell Dynamics of Competing Cell Populations

Having collected detailed quantitative information on the cellular parameters affected during competition, we sought to extrapolate how cell competition affects stem cell dynamics using biophysical modeling. Recent studies of the *Drosophila* posterior midgut (de Navascués et al., 2012) show that, in common with many cycling vertebrate tissues (Simons and Clevers, 2011), intestinal stem cells follow a pattern of population asymmetric self-renewal, in which stem cell loss through differentiation is perfectly compensated by the division of neighboring ISCs. A hallmark of this behavior is that the distribution of clone sizes converges onto a scaling behavior in which the chance of finding a clone larger than a multiple of the average remains constant over time (Klein and Simons, 2011; de Navascués et al., 2012). Moreover, in the epithelial arrangement of the midgut, cumulative clone-size distributions are predicted to be exponential, whereas the average size of the surviving clones grows approximately linearly with time.

We first addressed the clonal dynamics of the control wild-type and control $M^{-/+}$ epithelium (i.e., wild-type clones in a wild-type background and $M^{-/+}$ clones in a $M^{-/+}$ background) as a basis before turning to consider the dynamics of competing cells. Details of the modeling procedure, which mirror the methods introduced in de Navascués et al. (2012), are detailed in Experimental Procedures. Briefly, to model the dynamics of stem cells and their differentiated progeny, we considered a simple lattice model in which ISCs form a single equipotent population distributed uniformly within the epithelium. Alongside ISCs, each lattice site is associated with a fixed number of differentiating cells. To model turnover, we adopted an approach based on stochastic simulation in which a mature differentiated cell is chosen at random and removed. Following its loss, with a given probability either the ISC on the same site undergoes asymmetric cell division, giving rise to a replacement EC, or the ISC commits to EB/EC cell fate and is itself replaced by the symmetrical duplication of an ISC at a neighboring site. Alongside the overall cell-division rate (equivalently, under homeostasis, the loss rate of differentiated cells), the relative probability of symmetric versus asymmetric cell division defines in full the dynamics of the system. Using the datasets of control wild-type clones in Figure 3 and of $M^{-/+}$ control clones in Figure S2, we found that the clonal fate data showed the predicted convergence to a cumulative clone-size distribution of approximately exponential form (Figure 4A). To make a quantitative fit of the model dynamics to the data, we considered the distribution of clone sizes as measured by their Dl^+ ISC content (Figure 4A, insets). We found that the model provides a good fit to the measured cumulative clone-size distributions at all three time points in both the wild-type and the $M^{-/+}$ control (Figure 4A, insets) and predicts with good approximation the total cell-number distribution at all three time points (Figure 4A, main graphs). Thus, both control wild-type (as expected) and neutral $M^{-/+}$ ISCs from our experiments undergo population asymmetric self-renewal. Interestingly, from a fit to the 3- and 7-day time points, we observed that the average ISC division rate for $M^{-/+}$ cells is about a factor of 2 smaller than for wild-type cells, consistent with our mitotic index data (Figures S2A–S2C).

With this platform, we then turned to consider the behavior of competing wild-type ISCs in a $M^{-/+}$ background. Raw clone-size distributions revealed a qualitatively similar pattern to the wild-type controls, indicative of population asymmetry (Figure 4B). Importantly, however, consistent with the analysis from Figures

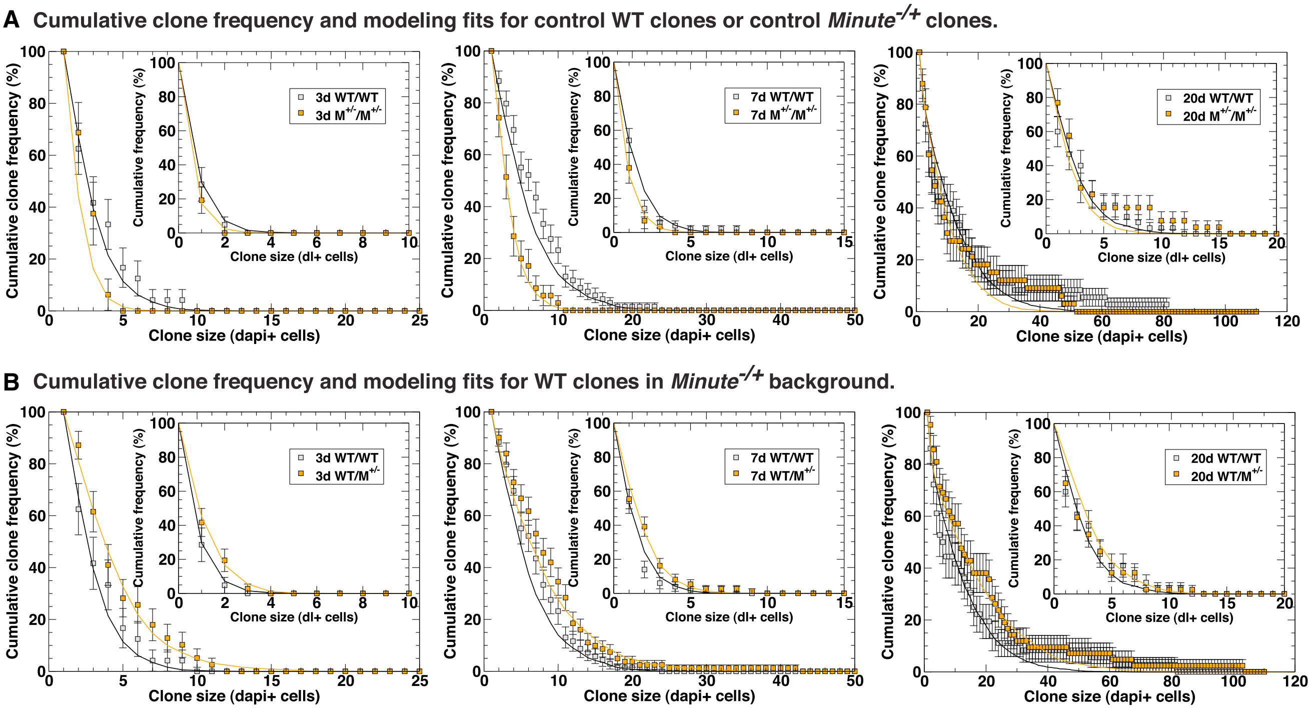

Figure 4. Biophysical Modeling of Stem Cell Dynamics during Cell Competition

(A) Cumulative clone-size distributions and modeling fits for neutral WT (in WT) (*hsflp/+; +/CyO; FRT82B, ubiGFP/FRT82B*) and neutral $M^{-/+}$ (in $M^{-/+}$) clones (*hsflp/+; FRT40, ubiRFP/FRT40; FRT82B, ubiGFP, RpS3/+*). The cumulative clone frequency describes the percentage of clones that have a size larger than the given value. The panels show distributions at 3 (left), 7 (middle), and 20 (right) days ACI. The corresponding cumulative clone-size distribution for Dl^+ cells is shown in the insets.

(B) Cumulative clone-size distributions and modeling fits for competing WT clones in the $M^{-/+}$ (*hsflp/+; FRT82B, ubiGFP, RpS3/FRT82B*) background and for neutral WT clones in the WT background (same as in A) as reference at 3 (left), 7 (middle), and 20 (right) days ACI.

Throughout, points indicate data and continuous lines indicate the corresponding modeling fits. Error bars denote SEM, adjusted appropriately for percentages and normalized against the total clone number at each time point. To eliminate single-EB clones that are generated as part of the mitotic recombination event, single-cell clones were excluded from total clone-size distributions.

3C, 3F, and 3H, the analysis of average clone size showed a small but significant increase over wild-type controls at all three time points (Figure 4B), indicating that wild-type clones display a proliferative advantage when they are in the presence of neighboring $M^{-/+}$ cells, leading to an accelerated clonal expansion of surviving clones.

We next used our model to fit the dynamics of wild-type clones in the $M^{-/+}$ background, looking for a minimal adaptation of the parameters, which would capture the observed behavior. In particular, based on our observation that cell competition increases proliferation (Figure 3F) and symmetric self-renewal (Figure 3H), we introduced a moderate (25%) increase in the number of divisions. Given that our data showed a localized increase in cell loss in surrounding $M^{-/+}$ cells (Figure 1), we introduced a local increase in the loss rate of neighboring $M^{-/+}$ cells to exactly compensate for the increase in divisions in wild-type cells, thus maintaining the system at homeostasis. This corresponds to an ~11-fold localized increase in loss rate compared to control $M^{-/+}$ cells (see Supplemental Experimental Procedures). Importantly, the two combined adjustments allowed us to obtain a good agreement of the model with the experimental data, both when considering Dl^+ cells only (Figure 4B, insets) and when considering the total clone size (Figure 4B, main graphs). Thus, we conclude that competing wild-type ISCs also display population asymmetry and that a modest increase in proliferation, balanced by localized biased tissue loss in surrounding $M^{-/+}$ cells, can account for the clonal expansion of competing wild-type clones. This confirms that proliferation increase and biased tissue loss are the two most prominent changes that cell competition induces on adult tissue dynamics.

Chronic JNK Activation in $M^{-/+}$ Cells Promotes the Clonal Expansion of Healthy Tissue

Our data show that when cells of different fitness coexist in the adult fly gut, weak cells undergo frequent cell death whereas fit cells boost their tissue-colonization ability. Cell death is known to stimulate proliferation in nearby cells in several tissues, including the fly gut, through a phenomenon known as compensatory proliferation (Fan and Bergmann, 2008; Amcheslavsky et al., 2009). In addition, inhibiting cell death has been shown to block the overproliferation of fit cells during cell competition (de la Cova et al., 2004, 2014; Li and Baker, 2007). We therefore wondered whether protecting $M^{-/+}$ cells from cell competition-induced apoptosis could mitigate the overgrowth of wild-type clones. Interestingly, we found that expressing the apoptosis inhibitor Diap1, which can effectively inhibit cell death in this tissue

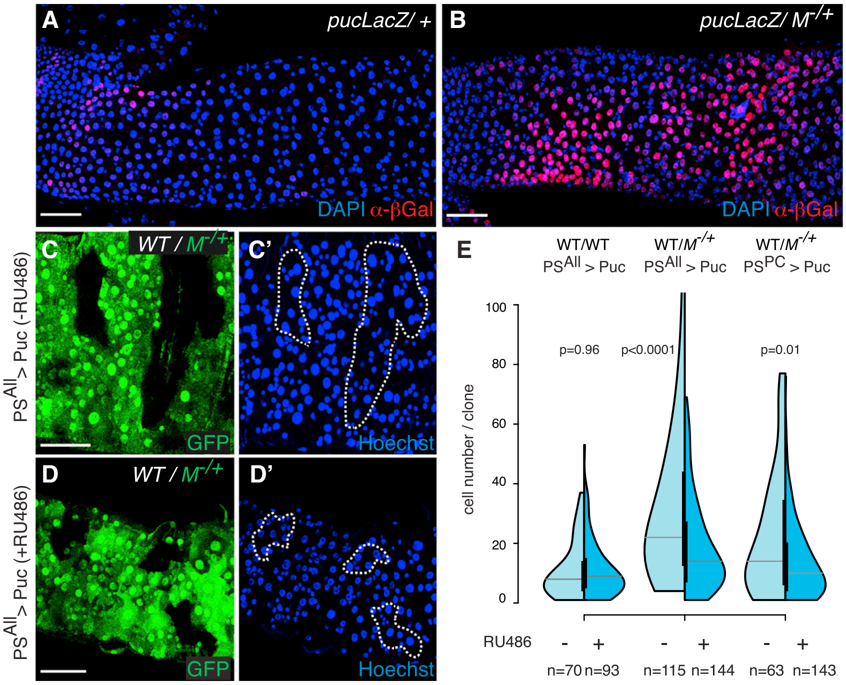

Figure 5. JNK Activation Promotes the Clonal Expansion of Wild-Type Cells

(A and B) *puc*-LacZ expression (α-β-Gal staining) in 3-day-old WT (*FRT82B, puc^{A251}/TM6B*) (A) and $M^{-/+}$ (B) guts (*FRT82B, puc^{A251}/FRT82B, RpS3**).

(C and D) Representative images of WT clones in $M^{-/+}$ guts with (D and D′) or without (C and C′) continued expression of Puc in all progenitor cells and ECs from the time of clone induction (−RU486 and +RU486, C and D, respectively; *hsflp/+; PSwitch^{ALL}/UAS Puc; FRT82B, ubiGFP, RpS3/FRT82B*).

(E) Analysis of clone-size distributions for WT clones either in WT guts (left) or in $M^{-/+}$ guts (middle and right graphs), with (dark blue) or without (light blue) continued expression of Puc (as in C and D). Genotypes: left: *hsflp/+; PSwitch^{ALL}/UAS Puc; FRT82B, ubiGFP/FRT82B*; middle: *hsflp/+; PSwitch^{ALL}/UAS Puc; FRT82B, ubiGFP, RpS3/FRT82B*; right: *hsflp/+; PSwitch^{PC}/UAS Puc; FRT82B, ubiGFP, RpS3/FRT82B*. p values (Mann-Whitney test) are indicated above each experiment. Scale bars represent 50 μm. See also Figure S3.

(Figures S3A–S3D), did not reduce the overgrowth of wild-type cells, whether it was expressed in ECs (Figure S3E) or expressed in both ECs and progenitor cells (Figures S3F and S3G). This suggested that signals other than apoptosis-induced proliferation might be involved. It has been reported that several mutants whose cells are outcompeted by wild-type cells, including $M^{-/+}$, display chronic activation of the JNK signaling pathway in wing imaginal discs (Tamori and Deng, 2011). We therefore asked whether in the midgut, $M^{-/+}$ cells also display increased JNK signaling. Indeed, we observed higher expression of *puc*-LacZ, a transcriptional reporter of JNK activation, in $M^{-/+}$ midguts compared to control guts (Figures 5A and 5B), indicating that the pathway was activated.

When the JNK pathway becomes activated in the adult midgut, such as in response to infection, inflammation, and aging, it leads to autonomous and nonautonomous proliferation (Biteau et al., 2008; Buchon et al., 2009a). We therefore asked whether JNK activation contributes to the clonal expansion of competing wild-type cells, and expressed the JNK inhibitor Puckered (Puc) throughout competing midguts (in both progenitor cells and ECs; Figures 5C and 5D). Interestingly, although JNK inhibition had no effect on the growth of control wild-type clones (Figure 5E, left), it significantly reduced the expansion of competing wild-type clones (Figure 5E, center). This was not an indirect consequence of suppressing loser cell death, as we could still observe dying $M^{-/+}$ ECs neighboring wild-type clones (Figure S3H). A similar result was obtained by overexpressing Puc only in the differentiated ECs (Figure 5E, right), where JNK activation is mostly observed (Figure 5B), suggesting that JNK may fuel stem cell proliferation nonautonomously. Analogous results were obtained by expressing the dominant-negative version of JNK (JNK^{DN}; data not shown). Taken together, these data suggest that in this tissue, increased cell death is not necessary to promote the clonal expansion of fitter cells. Instead, the overgrowth of healthy cells during competition depends on JNK signaling activation.

$M^{-/+}$ Cells Stimulate Wild-Type Tissue Growth via JNK-Dependent Production of Unpaired-3

We next addressed how JNK signaling induces the proliferation of fitter cells. A potential candidate was the secreted JAK-STAT cytokine Unpaired-3 (Upd-3), because it has been shown that JNK signaling can activate Upd-3 expression, which mediates proliferation, tissue repair, and in some cases tumor growth (Pastor-Pareja et al., 2008; Beebe et al., 2010; Buchon et al., 2009b; Wu et al., 2010; Osman et al., 2012). Indeed, we found that $M^{-/+}$ guts display robust *upd-3* activation (detected using *upd3*-Gal4 and UAS-GFP; Agaisse et al., 2003; Figures 6A and 6B). Consistently, JAK-STAT activity (reported by 10×Stat-GFP; Bach et al., 2007) was higher in $M^{-/+}$ guts compared to control (Figures S4A and S4B). In addition, we found that inhibiting JNK signaling in ECs (with JNK^{DN}) was sufficient to restore JAK-STAT activity in $M^{-/+}$ guts back to wild-type levels (Figures S4A–S4C), indicating that JAK-STAT activation is downstream of JNK signaling. To assess whether Upd-3 is the proliferative signal boosting wild-type tissue overgrowth during cell competition, we tested whether reducing JAK-STAT signaling was able to contain the clonal expansion of wild-type cells in $M^{-/+}$ guts. Notably, expression of the dominant-negative Upd-3 receptor Domeless (Dome^{DN}) across the posterior midgut resulted in significant size reduction of competing wild-type clones (Figure 6E, left). In addition, reducing the *dome* gene dosage was able to contain substantially the overgrowth of wild-type clones in competing conditions (Figures 6C and 6D; Figure 6E, right), whereas it had no effect on clone size in control wild-type guts (Figure 6E, center). Altogether, we conclude that Upd-3, produced by $M^{-/+}$ cells downstream of chronic JNK signaling, fuels

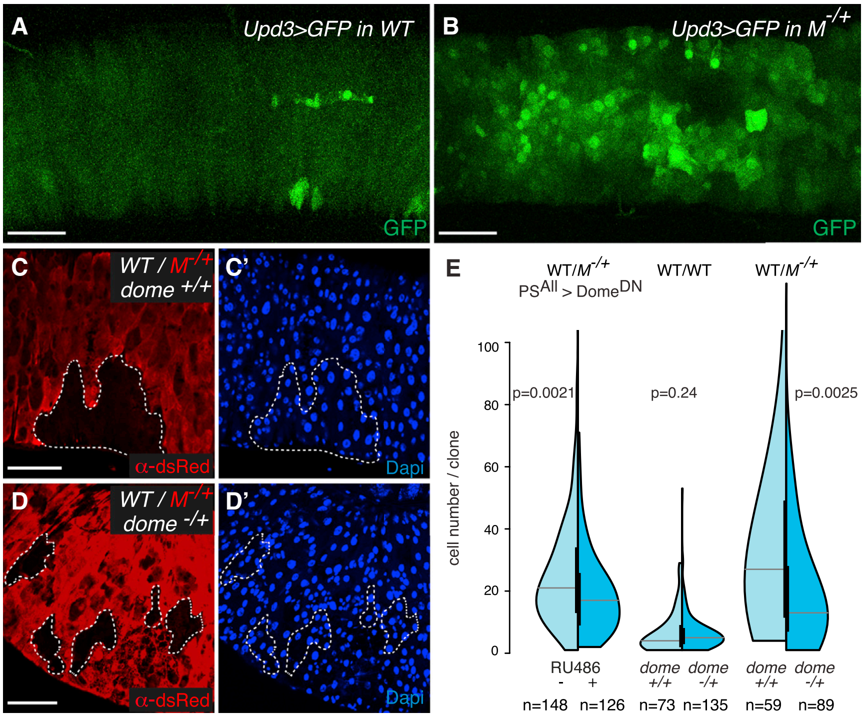

Figure 6. JAK-STAT Activation Fuels the Clonal Expansion of Wild-Type Cells

(A and B) Expression of GFP driven by *upd3*-Gal4 in 5-day-old WT (A) (*Upd3Gal4, UASGFP/+; FRT82, tub-CD2-DsRed/+*) and $M^{-/+}$ (B) guts (*Upd3Gal4, UASGFP/+; FRT82, tub-CD2-DsRed, RpS3/+*).

(C and D) Representative images of WT clones either in $M^{-/+}$ guts (C and C′; *hsflp/+;; FRT82B, tub-CD2-DsRed, RpS3/FRT82B*) or in $M^{-/+}$ guts with reduced JAK-STAT activity by removal of one functional copy of dome (*dome*$^{-/+}$) (D and D′; *hsflp/dome*^{G0218}*;; FRT82B, tub-CD2-DsRed, RpS3/FRT82B*).

(E) Analysis of clone-size distributions for WT clones either in WT guts or in $M^{-/+}$ guts, with (dark blue) or without (light blue) reduction of JAK-STAT pathway activity by expression of Dome^{DN} (PS^{All}, ±RU486) or by removal of one functional copy of dome (*dome*$^{-/+}$). Genotypes: left: *hsflp/+; PSwitch* ^{ALL}*/UAS Dome*^{DN}*; FRT82B, ubiGFP, RpS3/FRT82B*; middle: [WT/WT dome$^{+/+}$] *hsflp/+;; FRT82B, tub-CD2-DsRed/FRT82B;* [WT/WT dome$^{-/+}$] *hsflp/dome*^{G0218}*;; FRT82B, tub-CD2-DsRed/FRT82B*; right: [WT/M$^{-/+}$ dome$^{+/+}$] *hsflp/+;; FRT82B, tub-CD2-DsRed, RpS3/FRT82B*; [WT/M$^{-/+}$ dome$^{-/+}$] *hsflp/dome*^{G0218}*;; FRT82B, tub-CD2-DsRed, RpS3/FRT82B*. p values (Mann-Whitney test) are indicated above each experiment. Scale bars represent 50 μm. See also Figure S4.

the proliferative expansion of wild-type clones during cell competition in this tissue.

DISCUSSION

Recent studies have shown that cell competition can also take place in adult tissues (Oertel et al., 2006; Villa del Campo et al., 2014). Our work has taken this notion forward and delineated quantitatively how adult stem cells and tissue population dynamics are affected by cell competition (Figure 7A). In the subfit population, differentiated cells are killed by apoptosis followed by cell delamination; stem cells are also eliminated, possibly via induction of differentiation, as we have not detected dying stem cells. In parallel, as we show, the healthy tissue expands due to an increase in stem cell proliferation and self-renewal. Indeed, biophysical modeling shows that changes in these parameters of a magnitude comparable to what we observe experimentally is sufficient to recapitulate the stem cell dynamics of wild-type tissue undergoing Minute cell competition. Interestingly, accelerated proliferation of fitter stem cells has been seen in mouse embryonic stem cells using in vitro models of cell competition (Clavería et al., 2013; Sancho et al., 2013). However, in those studies, increased stem cell self-renewal has not been observed, probably because stemness in vitro is artificially maintained by exogenous factors in the culture medium.

Tissue Dynamics and Active Cell Competition

In many adult homeostatic tissues, stem cells stochastically differentiate or self-renew, and this leads to clonal extinction balanced by clonal expansion (Klein et al., 2007; Lopez-Garcia et al., 2010; Snippert et al., 2010; de Navascués et al., 2012). This is known as neutral drift competition, because through this process, stem cell compartments stochastically tend toward monoclonality (Klein et al., 2007; Lopez-Garcia et al., 2010; Snippert et al., 2010; de Navascués et al., 2012). It has also been shown that stem cell competition can be nonneutral (i.e., biased) when stem cells acquire a cell-autonomous advantage (Wang et al., 2009; Snippert et al., 2014). In these cases, the bias derives from *intrinsic* differences (e.g., faster proliferation) and does not rely on cell interactions. Here we show instead that in adult homeostatically maintained tissues, competitive cell interactions can act as *extrinsic* cues that actively modify stem cell behavior, and that this confers on winners an advantage (e.g., as we observe, increased proliferation rate and self-renewal) and on losers a disadvantage (e.g., as we observe, induced cell death), influencing tissue colonization. It is important to note that clones of wild-type cells that have lost proliferative capability because they are devoid of ISCs are equally able to induce death in neighboring $M^{-/+}$ cells. This rules out the possibility that physical displacement due to a faster clonal expansion is the cause of cell competition in this case. This process instead, like the recent reports of cell competition in the mouse heart (Villa del Campo et al., 2014) and fly nervous system (Merino et al., 2015), likely corresponds to the adult equivalent of the cellular competition observed in developing tissues (Morata and Ripoll, 1975; de Beco et al., 2012; Vivarelli et al., 2012; Vincent et al., 2013).

The Inflammatory Response Is Integral to Minute Cell Competition in the Midgut

Our work shows that $M^{-/+}$ midguts suffer from a chronic inflammatory response, which through JNK signaling activation and the ensuing production of the JAK-STAT ligand Upd-3 promotes wild-type tissue overgrowth (Figure 7B). Thus, in this tissue, the

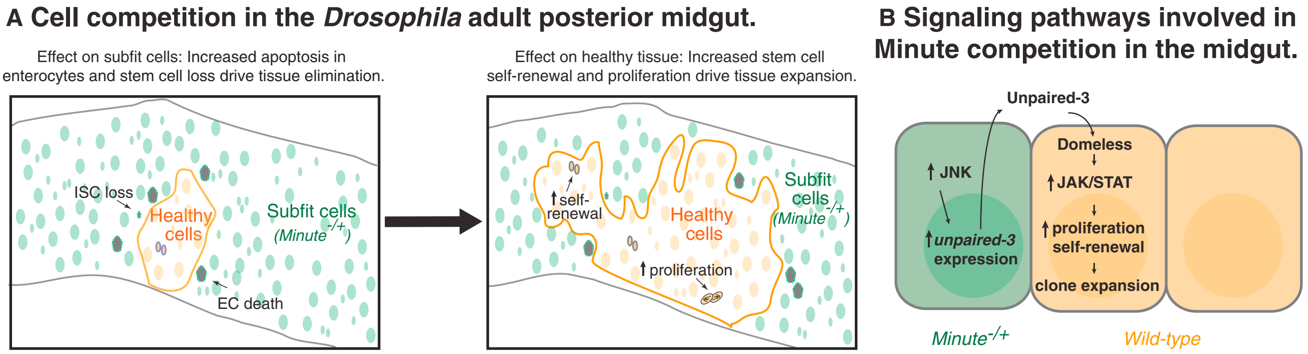

Figure 7. Adult Tissue Dynamics and Signaling Pathways during Minute Competition

(A) Active cell competition between healthy and subfit cells in the *Drosophila* homeostatic midgut causes loss of subfit tissue. $M^{-/+}$ differentiated cells are eliminated via apoptosis and $M^{-/+}$ ISCs are lost, possibly by cell death or induction of differentiation (left). Conversely, the presence of unhealthy tissue promotes expansion of healthy stem cells and their progeny. ISCs increase their proliferation rate and their symmetric self-renewal, which fuels clonal expansion (right).
(B) $M^{-/+}$ cells drive the clonal expansion of fit cells through an inflammatory-like response. Chronic JNK signaling activation in $M^{-/+}$ cells activates constitutive expression of the JAK-STAT ligand Unpaired-3. Secreted Unpaired-3, via binding to its receptor Dome, activates JAK-STAT signaling, stimulating the proliferative expansion of wild-type clones during cell competition.

overproliferation of winner cells stems from the increased availability of proliferative signals in the $M^{-/+}$ environment. Our results suggest that wild-type cells respond more efficiently than $M^{-/+}$ cells to this proliferation stimulus, and that this difference results in their preferential overgrowth, contributing to cell competition. It has long been suggested that cell competition may result from the limiting availability of growth factors, which would compromise the viability of loser cells (Raff, 1992; Moreno et al., 2002). Here we find instead that excess production of a growth factor (Upd-3) can boost cell competition by promoting preferential proliferation of fitter cells. Given that JNK and JAK-STAT are frequently activated in response to stress or deleterious mutations (e.g., Igaki et al., 2006; Ohsawa et al., 2012), it would be interesting to test whether this is a general mechanism used by loser cells to promote the overgrowth of fitter neighbors. Notably, differences in JAK-STAT signaling are sufficient to trigger cell competition (Rodrigues et al., 2012) and, consistent with this, reducing JAK-STAT signaling in wild-type cells compromises their ability to eliminate *scribble*$^{-/-}$ losers (Schroeder et al., 2013). Thus, increased JAK-STAT signaling may in addition provide wild-type cells with a heightened fitness state and help promote the elimination of $M^{-/+}$ losers.

Adult Cell Competition and the Phenomenon of Mosaic Revertants

Ribosomal mutations are linked with many adult disorders, not just in *Drosophila* (Casad et al., 2011) but more importantly in humans, where they are associated with a number of severe pathologies, collectively known as ribosomopathies (Narla and Ebert, 2010). Given that 79 proteins make up the eukaryotic ribosome (and several more are involved in ribosomal production) and that many *Minute* mutations are dominant, the sporadic insurgence of $M^{-/+}$ cells in adult tissues is likely to be one of the most common spontaneous generations of somatic mutant cells in our bodies. The elimination of these cells via cell competition is likely to play an unappreciated role in maintaining healthy adult tissues (Titen and Golic, 2008; McNamee and Brodsky, 2009; Baker, 2011).

A striking feature emerging from our results is that, in response to cell competition, normal cells can efficiently repopulate adult tissues, thus effectively replacing potentially diseased cells. This bears striking resemblance to the phenomenon of mosaic revertants, observed in a number of human skin and blood diseases (Lai-Cheong et al., 2011; Jonkman and Pasmooij, 2012). Spontaneous sporadic reversion of genetically inherited, disease-bearing mutations leads to the generation of revertant cells, which effectively repopulate tissues, at times ameliorating the condition (Lai-Cheong et al., 2011; Jonkman and Pasmooij, 2012). In some instances, the revertants' expansion is so efficient that selective advantage has been proposed (Choate et al., 2010). Intriguingly, ichthyosis with confetti, a skin disease characterized by confetti-like appearance of revertant skin spots, is associated with a mutation in Keratin 10 (Choate et al., 2010), which, due to its nucleolar mislocalization, could affect ribosome production similar to $M^{-/+}$ mutants. Thus, based on our findings, it is tentative to speculate that selective advantage in mosaic revertants could in some cases be driven by cell competition.

EXPERIMENTAL PROCEDURES

Cell Counting

All quantifications were done manually throughout the volume of 3D reconstructions of z stacks. Clone sizes were calculated as the number of 4′,6-diamidino-2-phenylindole (DAPI)-positive cells per clone. To determine the proportion of delaminating cells in the guts (Figures 1A–1C), we analyzed confocal stacks spanning the whole gut. Cells that were detached from the epithelium (i.e., either delaminating or found in the lumen) were identified and assigned to the 2×GFP ($M^{-/+}$ cells) or 1×GFP or 0×GFP (pseudo-WT cells) population according to fluorescence intensity. The proportion of $M^{-/+}$ cells delaminating was determined by establishing the ratio 2×GFP delaminating/total 2×GFP cells (Figure 1C, $M^{-/+}$). The proportion of (pseudo) WT cells delaminating was determined by establishing the ratio of combined 0×GFP and 1×GFP (DAPI-positive) delaminating cells/total 0×GFP and 1×GFP cells (Figure 1C, WT).

To count cells "around" clones (Figure 1E, near), we counted all cells surrounding a clone within two cell diameters in the 3D volume (this, depending on clone size, corresponds to between 10% and 30% of the GFP^{+} population,

both in control and competing guts). To characterize cells not adjacent to clones (Figure 1E, far), we counted all cells minus those within and around clones. Figure 3G shows the ratios: proportion of EdU^+ Dl^+ cells "near" clones/proportion of EdU^+ Dl^+ cells "far" from clones.

Sytox Staining

To detect dying cells, we used Sytox orange (5 mM stock in DMSO; Life Technologies; S11368) diluted 1/10,000 in 5% sucrose/water. Flies were transferred to an empty vial containing a piece of Whatman paper soaked with Sytox solution 4–5 hr prior to dissection. Because Sytox staining is not fixable, we imaged the guts within 1 day of staining.

EdU Stainings and Quantifications

To analyze the proportion of stem cells in a replicative state, mosaic female flies were analyzed 7 days ACI. Guts were dissected out in Schneider's medium (Sigma) and incubated for 30 min in a solution of 10 μM EdU/Schneider's medium. After rinsing, guts were fixed and processed for immunostaining as described in Supplemental Experimental Procedures. Guts were then processed for Click-IT EdU detection according to the manufacturer's instructions (Invitrogen; C10338). Guts were washed and mounted in Vectashield on a glass slide. The average fraction of Dl^+ stem cells labeled with EdU following a 30-min incubation was 0.15 ± 0.06 (SD) for WT/WT guts and 0.19 ± 0.08 (SD) for WT/$M^{-/+}$ guts. We processed the two genotypes in parallel and dissected 80 guts per condition in a single experiment, as stringent parameters were used to retain guts for analysis: only guts containing at least 10% EdU^+ stem cells were retained for further analysis to minimize variability due to EdU incorporation. For the quantification of EdU^+ stem cells *within* clones of WT cells, to avoid statistical artifacts, we further filtered out any gut in which the total number of WT ISCs per gut (summing all clones) was lower than the minimum number of cells required to encounter statistically at least one EdU^+ stem cell (based on the proportion of EdU^+ stem cells in that gut).

Statistical Tests

Statistical analyses were done using Prism (GraphPad; version 5.0). p values were determined using the nonparametric Mann-Whitney test, except for Figure 1H (Fisher's exact test). The split violin graphs (generated in R; http://www.r-project.org) represent two datasets side by side where for each sample the full distribution of values is represented as a smooth histogram. This allows for direct comparison of the range and shape of such distributions. For each dataset, the horizontal gray bar represents the median and the vertical black box indicates the 25th and 75th percentiles. Error bars: in Figure S2C, the averages ± SD are represented; in Figures 2C, 2D, 3F, and 3H, the averages ± SEM are represented (for Figures 2C and 2D, these values are expressed as a fraction of the values at 4 days ACI); in Figure 3D, the error (e) associated with the product (P) of the average clone size*average clone frequency is calculated according to the following formula: $e/P = \sqrt{[(\text{SEM of average clone size/average clone size})^2 + (\text{SD of average clone frequency/average clone frequency})^2]}$.

Biophysical Modeling

The quantitative analysis of the clonal fate data relies upon the development of a biophysical modeling scheme, previously formulated for the study of homeostatic turnover of the *Drosophila* wild-type posterior midgut (de Navascués et al., 2012). Briefly, to model the dynamics of stem cells and their differentiated progeny, we considered a simple lattice model in which ISCs form a single equipotent population that is distributed uniformly within the epithelium. Alongside ISCs, each lattice site was associated with a fixed number of differentiating cells. To model turnover, we adopted an approach based on stochastic simulation in which a mature differentiated cell is chosen at random and removed. Following its loss, with a given probability, either the ISC on the same site undergoes asymmetric cell division, giving rise to a replacement EB, or the ISC commits to EB cell fate and is itself replaced by the symmetrical duplication of an ISC at a neighboring site. As previously shown (de Navascués et al., 2012; Klein and Simons, 2011), in such a two-dimensional model system, the clone-size distribution takes an exponential form. More precisely, the cumulative clone-size distribution, defined as the probability, $P_n(t)$, of finding a clone with a size of more than n cells, takes the form $P_n(t) = \text{Exp}[-n/n(t)]$. Further details on how this modeling scheme was applied to competing tissues is detailed in Supplemental Experimental Procedures.

SUPPLEMENTAL INFORMATION

Supplemental Information includes Supplemental Experimental Procedures and four figures and can be found with this article online at http://dx.doi.org/10.1016/j.devcel.2015.06.010.

ACKNOWLEDGMENTS

This work was supported by a Cancer Research UK Programme grant to E.P. (supporting E.P. and G.K. [A12460]), Royal Society University Research fellowship to E.P. (UF090580), EMBO Long-Term fellowship (ALTF 1476-2012), NWO Rubicon grant (825.12.027), Dutch Cancer Society fellowship to S.J.E.S. (BUIT-2013-5847), Wellcome Trust PhD studentship to I.K., Wellcome Trust grant to B.D.S. (098357/Z/12/Z), and Core grant funding from the Wellcome Trust Core (092096) and CRUK (C6946/A14492). We thank Rafael E. Carazo Salas, Helen Skaer, and Valentina Greco for discussions and/or critical reading of the manuscript. We also thank the Bloomington Stock Center (Indiana University) and scientists listed in Supplemental Experimental Procedures for *Drosophila* strains, as well as the Developmental Studies Hybridoma Bank for antibodies.

Received: March 5, 2014
Revised: May 13, 2015
Accepted: June 11, 2015
Published: July 23, 2015

REFERENCES

Agaisse, H., Petersen, U.M., Boutros, M., Mathey-Prevot, B., and Perrimon, N. (2003). Signaling role of hemocytes in *Drosophila* JAK/STAT-dependent response to septic injury. Dev. Cell *5*, 441–450.

Amcheslavsky, A., Jiang, J., and Ip, Y.T. (2009). Tissue damage-induced intestinal stem cell division in *Drosophila*. Cell Stem Cell *4*, 49–61.

Bach, E.A., Ekas, L.A., Ayala-Camargo, A., Flaherty, M.S., Lee, H., Perrimon, N., and Baeg, G.H. (2007). GFP reporters detect the activation of the *Drosophila* JAK/STAT pathway in vivo. Gene Expr. Patterns *7*, 323–331.

Baker, N.E. (2011). Cell competition. Curr. Biol. *21*, R11–R15.

Beebe, K., Lee, W.C., and Micchelli, C.A. (2010). JAK/STAT signaling coordinates stem cell proliferation and multilineage differentiation in the *Drosophila* intestinal stem cell lineage. Dev. Biol. *338*, 28–37.

Biteau, B., Hochmuth, C.E., and Jasper, H. (2008). JNK activity in somatic stem cells causes loss of tissue homeostasis in the aging *Drosophila* gut. Cell Stem Cell *3*, 442–455.

Bondar, T., and Medzhitov, R. (2010). p53-mediated hematopoietic stem and progenitor cell competition. Cell Stem Cell *6*, 309–322.

Buchon, N., Broderick, N.A., Poidevin, M., Pradervand, S., and Lemaitre, B. (2009a). *Drosophila* intestinal response to bacterial infection: activation of host defense and stem cell proliferation. Cell Host Microbe *5*, 200–211.

Buchon, N., Broderick, N.A., Chakrabarti, S., and Lemaitre, B. (2009b). Invasive and indigenous microbiota impact intestinal stem cell activity through multiple pathways in *Drosophila*. Genes Dev. *23*, 2333–2344.

Casad, M.E., Abraham, D., Kim, I.M., Frangakis, S., Dong, B., Lin, N., Wolf, M.J., and Rockman, H.A. (2011). Cardiomyopathy is associated with ribosomal protein gene haplo-insufficiency in *Drosophila melanogaster*. Genetics *189*, 861–870.

Choate, K.A., Lu, Y., Zhou, J., Choi, M., Elias, P.M., Farhi, A., Nelson-Williams, C., Crumrine, D., Williams, M.L., Nopper, A.J., et al. (2010). Mitotic recombination in patients with ichthyosis causes reversion of dominant mutations in KRT10. Science *330*, 94–97.

Clavería, C., Giovinazzo, G., Sierra, R., and Torres, M. (2013). Myc-driven endogenous cell competition in the early mammalian embryo. Nature *500*, 39–44.

de Beco, S., Ziosi, M., and Johnston, L.A. (2012). New frontiers in cell competition. Dev. Dyn. *241*, 831–841.

de la Cova, C., Abril, M., Bellosta, P., Gallant, P., and Johnston, L.A. (2004). *Drosophila* Myc regulates organ size by inducing cell competition. Cell *117*, 107–116.

de la Cova, C., Senoo-Matsuda, N., Ziosi, M., Wu, D.C., Bellosta, P., Quinzii, C.M., and Johnston, L.A. (2014). Supercompetitor status of *Drosophila* Myc cells requires p53 as a fitness sensor to reprogram metabolism and promote viability. Cell Metab. *19*, 470–483.

de Navascués, J., Perdigoto, C.N., Bian, Y., Schneider, M.H., Bardin, A.J., Martínez-Arias, A., and Simons, B.D. (2012). *Drosophila* midgut homeostasis involves neutral competition between symmetrically dividing intestinal stem cells. EMBO J. *31*, 2473–2485.

Fan, Y., and Bergmann, A. (2008). Distinct mechanisms of apoptosis-induced compensatory proliferation in proliferating and differentiating tissues in the *Drosophila* eye. Dev. Cell *14*, 399–410.

Igaki, T., Pagliarini, R.A., and Xu, T. (2006). Loss of cell polarity drives tumor growth and invasion through JNK activation in *Drosophila*. Curr. Biol. *16*, 1139–1146.

Issigonis, M., Tulina, N., de Cuevas, M., Brawley, C., Sandler, L., and Matunis, E. (2009). JAK-STAT signal inhibition regulates competition in the *Drosophila* testis stem cell niche. Science *326*, 153–156.

Jiang, H., and Edgar, B.A. (2012). Intestinal stem cell function in *Drosophila* and mice. Curr. Opin. Genet. Dev. *22*, 354–360.

Jiang, H., Patel, P.H., Kohlmaier, A., Grenley, M.O., McEwen, D.G., and Edgar, B.A. (2009). Cytokine/Jak/Stat signaling mediates regeneration and homeostasis in the *Drosophila* midgut. Cell *137*, 1343–1355.

Jin, Z., Kirilly, D., Weng, C., Kawase, E., Song, X., Smith, S., Schwartz, J., and Xie, T. (2008). Differentiation-defective stem cells outcompete normal stem cells for niche occupancy in the *Drosophila* ovary. Cell Stem Cell *2*, 39–49.

Jonkman, M.F., and Pasmooij, A.M. (2012). Realm of revertant mosaicism expanding. J. Invest. Dermatol. *132*, 514–516.

Klein, A.M., and Simons, B.D. (2011). Universal patterns of stem cell fate in cycling adult tissues. Development *138*, 3103–3111.

Klein, A.M., Doupé, D.P., Jones, P.H., and Simons, B.D. (2007). Kinetics of cell division in epidermal maintenance. Phys. Rev. E Stat. Nonlin. Soft Matter Phys. *76*, 021910.

Lai-Cheong, J.E., McGrath, J.A., and Uitto, J. (2011). Revertant mosaicism in skin: natural gene therapy. Trends Mol. Med. *17*, 140–148.

Lee, W.C., Beebe, K., Sudmeier, L., and Micchelli, C.A. (2009). Adenomatous polyposis coli regulates *Drosophila* intestinal stem cell proliferation. Development *136*, 2255–2264.

Li, W., and Baker, N.E. (2007). Engulfment is required for cell competition. Cell *129*, 1215–1225.

Lin, G., Xu, N., and Xi, R. (2008). Paracrine Wingless signalling controls self-renewal of *Drosophila* intestinal stem cells. Nature *455*, 1119–1123.

Lopez-Garcia, C., Klein, A.M., Simons, B.D., and Winton, D.J. (2010). Intestinal stem cell replacement follows a pattern of neutral drift. Science *330*, 822–825.

Martín, F.A., Herrera, S.C., and Morata, G. (2009). Cell competition, growth and size control in the *Drosophila* wing imaginal disc. Development *136*, 3747–3756.

Marusyk, A., Porter, C.C., Zaberezhnyy, V., and DeGregori, J. (2010). Irradiation selects for p53-deficient hematopoietic progenitors. PLoS Biol. *8*, e1000324.

Marygold, S.J., Roote, J., Reuter, G., Lambertsson, A., Ashburner, M., Millburn, G.H., Harrison, P.M., Yu, Z., Kenmochi, N., Kaufman, T.C., et al. (2007). The ribosomal protein genes and Minute loci of *Drosophila melanogaster*. Genome Biol. *8*, R216.

Mathur, D., Bost, A., Driver, I., and Ohlstein, B. (2010). A transient niche regulates the specification of *Drosophila* intestinal stem cells. Science *327*, 210–213.

McNamee, L.M., and Brodsky, M.H. (2009). p53-independent apoptosis limits DNA damage-induced aneuploidy. Genetics *182*, 423–435.

Menthena, A., Koehler, C.I., Sandhu, J.S., Yovchev, M.I., Hurston, E., Shafritz, D.A., and Oertel, M. (2011). Activin A, p15INK4b signaling, and cell competition promote stem/progenitor cell repopulation of livers in aging rats. Gastroenterology *140*, 1009–1020.

Merino, M.M., Rhiner, C., Portela, M., and Moreno, E. (2013). "Fitness fingerprints" mediate physiological culling of unwanted neurons in *Drosophila*. Curr. Biol. *23*, 1300–1309.

Merino, M.M., Rhiner, C., Lopez-Gay, J.M., Buechel, D., Hauert, B., and Moreno, E. (2015). Elimination of unfit cells maintains tissue health and prolongs lifespan. Cell *160*, 461–476.

Micchelli, C.A., and Perrimon, N. (2006). Evidence that stem cells reside in the adult *Drosophila* midgut epithelium. Nature *439*, 475–479.

Morata, G., and Ripoll, P. (1975). Minutes: mutants of *Drosophila* autonomously affecting cell division rate. Dev. Biol. *42*, 211–221.

Moreno, E., Basler, K., and Morata, G. (2002). Cells compete for decapentaplegic survival factor to prevent apoptosis in *Drosophila* wing development. Nature *416*, 755–759.

Narla, A., and Ebert, B.L. (2010). Ribosomopathies: human disorders of ribosome dysfunction. Blood *115*, 3196–3205.

Oertel, M., Menthena, A., Dabeva, M.D., and Shafritz, D.A. (2006). Cell competition leads to a high level of normal liver reconstitution by transplanted fetal liver stem/progenitor cells. Gastroenterology *130*, 507–520.

Ohlstein, B., and Spradling, A. (2006). The adult *Drosophila* posterior midgut is maintained by pluripotent stem cells. Nature *439*, 470–474.

Ohlstein, B., and Spradling, A. (2007). Multipotent *Drosophila* intestinal stem cells specify daughter cell fates by differential Notch signaling. Science *315*, 988–992.

Ohsawa, S., Sato, Y., Enomoto, M., Nakamura, M., Betsumiya, A., and Igaki, T. (2012). Mitochondrial defect drives non-autonomous tumour progression through Hippo signalling in *Drosophila*. Nature *490*, 547–551.

Osman, D., Buchon, N., Chakrabarti, S., Huang, Y.T., Su, W.C., Poidevin, M., Tsai, Y.C., and Lemaitre, B. (2012). Autocrine and paracrine unpaired signaling regulate intestinal stem cell maintenance and division. J. Cell Sci. *125*, 5944–5949.

Pastor-Pareja, J.C., Wu, M., and Xu, T. (2008). An innate immune response of blood cells to tumors and tissue damage in *Drosophila*. Dis. Model. Mech. *1*, 144–154.

Raff, M.C. (1992). Social controls on cell survival and cell death. Nature *356*, 397–400.

Rhiner, C., Díaz, B., Portela, M., Poyatos, J.F., Fernández-Ruiz, I., López-Gay, J.M., Gerlitz, O., and Moreno, E. (2009). Persistent competition among stem cells and their daughters in the *Drosophila* ovary germline niche. Development *136*, 995–1006.

Rodrigues, A.B., Zoranovic, T., Ayala-Camargo, A., Grewal, S., Reyes-Robles, T., Krasny, M., Wu, D.C., Johnston, L.A., and Bach, E.A. (2012). Activated STAT regulates growth and induces competitive interactions independently of Myc, Yorkie, Wingless and ribosome biogenesis. Development *139*, 4051–4061.

Sancho, M., Di-Gregorio, A., George, N., Pozzi, S., Sánchez, J.M., Pernaute, B., and Rodríguez, T.A. (2013). Competitive interactions eliminate unfit embryonic stem cells at the onset of differentiation. Dev. Cell *26*, 19–30.

Schroeder, M.C., Chen, C.L., Gajewski, K., and Halder, G. (2013). A non-cell-autonomous tumor suppressor role for Stat in eliminating oncogenic scribble cells. Oncogene *32*, 4471–4479.

Simons, B.D., and Clevers, H. (2011). Strategies for homeostatic stem cell self-renewal in adult tissues. Cell *145*, 851–862.

Snippert, H.J., van der Flier, L.G., Sato, T., van Es, J.H., van den Born, M., Kroon-Veenboer, C., Barker, N., Klein, A.M., van Rheenen, J., Simons, B.D., and Clevers, H. (2010). Intestinal crypt homeostasis results from neutral competition between symmetrically dividing Lgr5 stem cells. Cell *143*, 134–144.

Snippert, H.J., Schepers, A.G., van Es, J.H., Simons, B.D., and Clevers, H. (2014). Biased competition between Lgr5 intestinal stem cells driven by oncogenic mutation induces clonal expansion. EMBO Rep. *15*, 62–69.

Tamori, Y., and Deng, W.M. (2011). Cell competition and its implications for development and cancer. J. Genet. Genomics *38*, 483–495.

Tamori, Y., and Deng, W.M. (2013). Tissue repair through cell competition and compensatory cellular hypertrophy in postmitotic epithelia. Dev. Cell *25*, 350–363.

Titen, S.W., and Golic, K.G. (2008). Telomere loss provokes multiple pathways to apoptosis and produces genomic instability in *Drosophila melanogaster*. Genetics *180*, 1821–1832.

Villa del Campo, C., Clavería, C., Sierra, R., and Torres, M. (2014). Cell competition promotes phenotypically silent cardiomyocyte replacement in the mammalian heart. Cell Rep. *8*, 1741–1751.

Vincent, J.P., Fletcher, A.G., and Baena-Lopez, L.A. (2013). Mechanisms and mechanics of cell competition in epithelia. Nat. Rev. Mol. Cell Biol. *14*, 581–591.

Vivarelli, S., Wagstaff, L., and Piddini, E. (2012). Cell wars: regulation of cell survival and proliferation by cell competition. Essays Biochem. *53*, 69–82.

Wang, Z., Li, G., Tse, W., and Bunting, K.D. (2009). Conditional deletion of STAT5 in adult mouse hematopoietic stem cells causes loss of quiescence and permits efficient nonablative stem cell replacement. Blood *113*, 4856–4865.

Williams, D.W., Kondo, S., Krzyzanowska, A., Hiromi, Y., and Truman, J.W. (2006). Local caspase activity directs engulfment of dendrites during pruning. Nat. Neurosci. *9*, 1234–1236.

Wu, M., Pastor-Pareja, J.C., and Xu, T. (2010). Interaction between Ras(V12) and scribbled clones induces tumour growth and invasion. Nature *463*, 545–548.

Developmental Cell

Supplemental Information

Cell Competition Modifies Adult Stem Cell and Tissue Population Dynamics in a JAK-STAT-Dependent Manner

Golnar Kolahgar, Saskia J.E. Suijkerbuijk, Iwo Kucinski, Enzo Z. Poirier, Sarah Mansour, Benjamin D. Simons, and Eugenia Piddini

Supplemental Figures and Legends

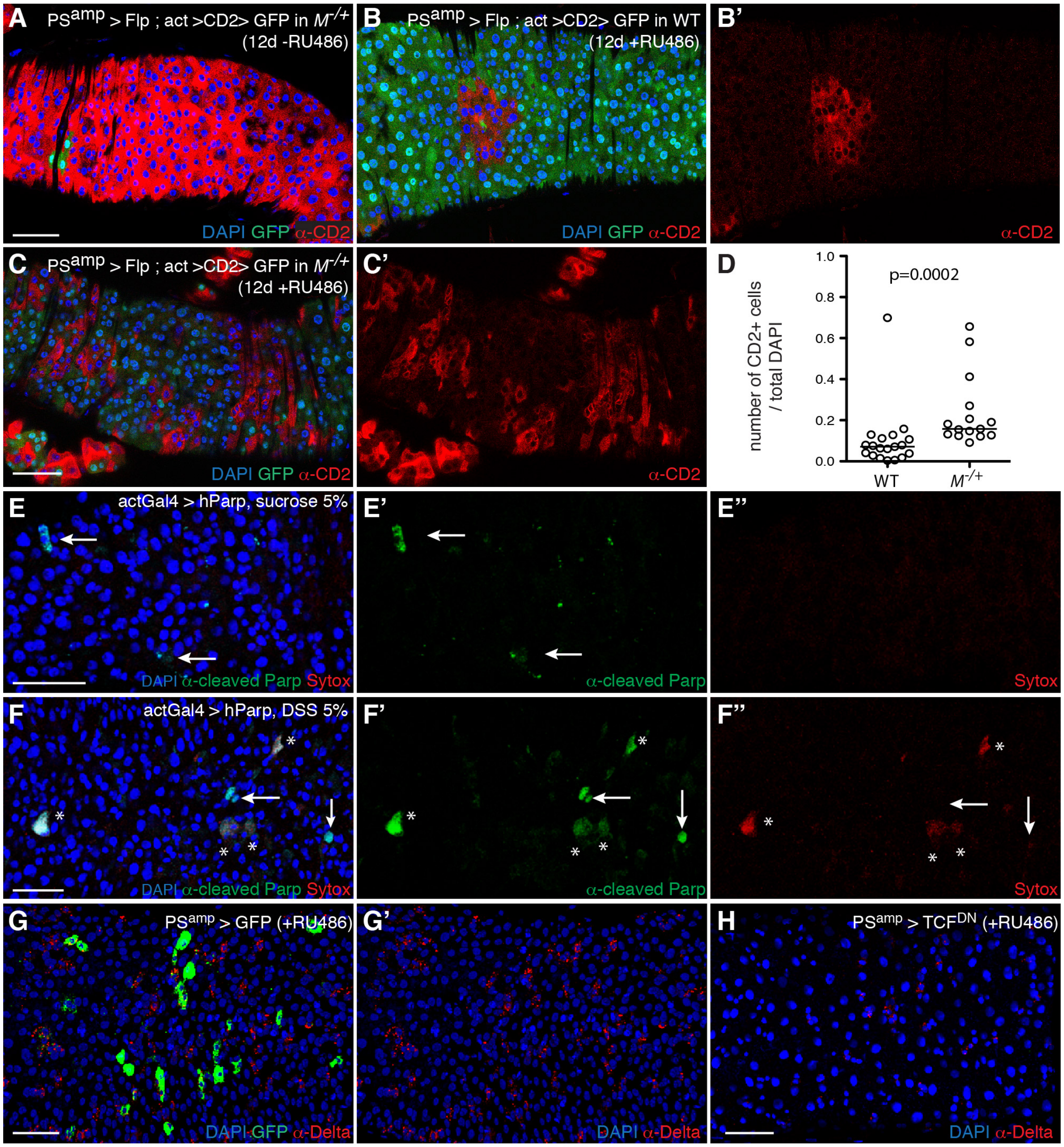

Figure S1 (related to Figure 1). Tissue turnover, cell death detection and induction of stem cell differentiation in the posterior midgut.

(A-D) Pulse-chase experiments show that posterior midgut turnover is slower in $M^{-/+}$ guts (*PS^{AMP}/UASFlp; act>CD2>Gal4, UASGFP/FRT82, RpS3*)* compared to wild-type guts (*PS^{AMP}/UASFlp; act>CD2>Gal4, UASGFP/*+). All posterior midgut cells are born CD2^{+} (red). Flp-induced excision of the CD2 cassette causes both CD2 loss and activation of Gal4, which

drives GFP expression (Jiang et al., 2009). Sustained UAS-Flp expression in progenitor cells by *PSwitch*^{AMP} and continual RU486 feeding turns all ISCs and their progeny GFP^{+} allowing to score how long old CD2^{+} cells perdure. (A) Control gut showing that in the absence of RU486, leakiness is minimal with most cells labelled by CD2 in 12-day old guts. (B, B') Representative WT gut and (C, C') $M^{-/+}$ gut after 12-day RU486 feeding; CD2 staining shows the extent of 12-day old cells still present. (D) Quantification of the proportion of 'old' CD2^{+} cells normalised to the total number of DAPI^{+} cells per gut. (n>15 guts for each genotype, p: Mann Whitney test). (E-F") Sytox incorporation and cleaved Parp staining detect cell death and apoptosis in the posterior midgut (*actGal4/UAS mCD8 Parp Venus*). (E-F") guts expressing *UAS mCD8 Parp Venus* under ubiquitous *actGal4* promoter show sporadic apoptosis by PARP cleavage (green) (E, E', arrows) and rare or no Sytox^{+} cells (red) (E, E") in sucrose fed controls, and frequent cleaved-PARP^{+} and/or Sytox^{+} cells in DSS fed guts (F-F"). Many Sytox^{+} cells are also PARP^{+} (F-F" asterisks). The occurrence of PARP^{+}/Sytox^{-} cells (F-F" arrows) suggests that PARP cleavage is an earlier and/or more sensitive readout than Sytox. (G-H) Expression of TCF^{DN} with *PSwitch*^{AMP} effectively induces ISC differentiation. (G, G') *PSwitch*^{AMP} driving the expression of GFP. GFP is detected in most but not all Dl^{+} cells after 2-day RU486 feeding (*PSwitch*^{AMP}*/UAS CD8 GFP*). (H) Induction of TCF^{DN} expression by 2-day RU486 supplementation drastically reduces the number of Dl^{+} ISCs (*PSwitch*^{AMP}*/UAS TCF*^{DN}), compared to control guts (G, G').

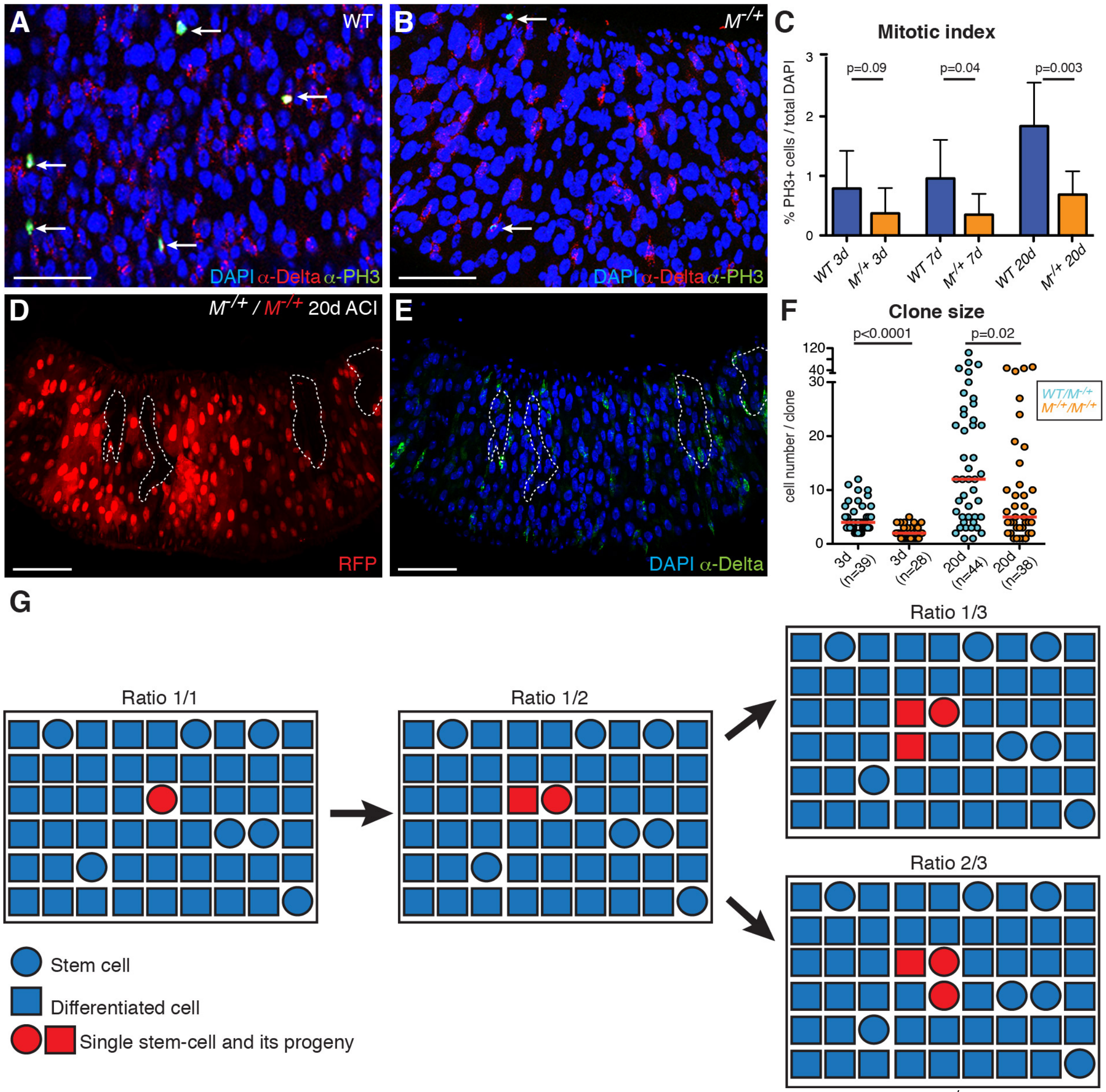

Figure S2 (related to Figure 3). Clonal growth dynamics for control *Minute*$^{-/+}$ tissue and scheme explaining how changes in clonal Delta/DAPI ratios inform on stem cell renewal rates.

(A-C) $M^{-/+}$ midguts have a reduced mitotic index. (A-B) Adult posterior midguts from 20 day old WT (A) (*yw*) or $M^{-/+}$ (B) (*FRT82B, RpS3, ubiGFP*/*TM6B*) flies, stained with the mitotic marker α-Phospho-Histone3 (α-PH3) (green, arrows) and the ISC marker α-Delta (red). (C) Quantification of the mitotic index (total PH3$^+$ cells/total DAPI$^+$ cells) for 3, 7, and 20 day old guts of genotype as in A and B (n>9 guts per condition; shown as average +/- SD). $M^{-/+}$ midguts display a significantly lower proliferative index compared to WT at all time points. p: Mann-Whitney test.

(D-F) Dynamics of clonal growth for $M^{-/+}$ clones in a $M^{-/+}$ background. (D-E) $M^{-/+}$ posterior midgut carrying control $M^{-/+}$ clones (marked by the absence of RFP (D) or by dotted lines (E) (*hsflp/+; FRT40 ubiRFP/FRT40; FRT82B, ubiGFP, RpS3*/+). Delta^{+} ISCs are in green (E). (F) Distribution of clone sizes shows that WT/$M^{-/+}$ (*hsflp/+;; FRT82B, ubiGFP, RpS3/FRT82B)* are significantly bigger that $M^{-/+}$/$M^{-/+}$ clones (genotype as in D-E) both at early and at late time points ACI (The WT/$M^{-/+}$ dataset is the same as in Fig. 3C). p: Mann-Whitney test (G) Schematics exemplifying how the ratio Dl^{+}/DAPI^{+} cells of an evolving single stem cell clone can inform on the frequency of its symmetric divisions.

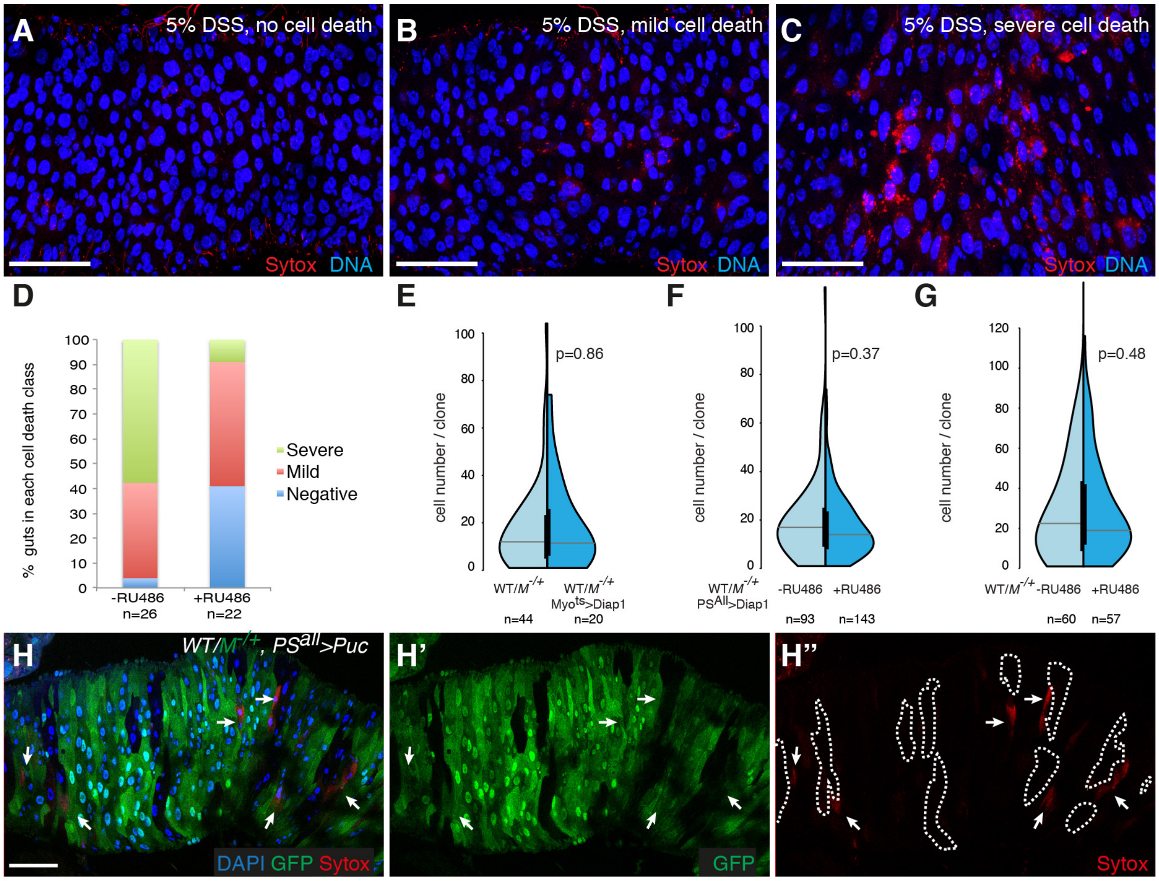

Figure S3 (related to Figure 5). Effect of Diap1 on clonal growth and effect of Puc on competition-induced apoptosis

(A-D) Diap1 expression reduces cell death in the posterior midgut. 10-12 day old flies of the genotype *hsflp/+; PSwitch^{ALL}/UAS Diap1; FRT82, ubiGFP/TM6, DfYFP,* with (+RU486) or without (-RU486) DIAP1 expression were fed 5% DSS for three days prior to Sytox incorporation. Guts were assigned to one of the three groups with negative (A), mild (B) or severe (C) cell death based on the extent of Sytox incorporation (n>22 guts for each condition). (D) shows the proportion of flies in each class for gut samples with (+RU486) or without (- RU486) DIAP1 expression. (E-G) Diap1 expression does not prevent the clonal expansion of WT cells in competing WT/*M*$^{-/+}$ guts. (E) Distribution of clone sizes for 20-day old WT clones in *M*$^{-/+}$ guts of the genotypes *hsflp/+;; FRT82B, ubiGFP, RpS3/FRT82B* (control) or *hsflp/+; MyoIAGal4, tubGal80ts/UAS Diap1; FRT82B, ubiGFP, RpS3/FRT82B* (with sustained Diap 1 expression by incubation at permissive temperature from the moment of clone induction); (F) WT clone size

distributions in guts of the genotype *hsflp/+; PSwitch^{ALL}/UAS Diap1; FRT82B, ubiGFP, RpS3/FRT82B* with or without DIAP1 expression in ECs and progenitor cells by RU486 supplementation. (G) Control experiments showing that clone size distribution is unaffected by RU486 supplementation alone (*hsflp/+;; FRT82B, ubiGFP, RpS3/FRT82B*). p: Mann Whitney test for E-G. (H-H") Puc expression in competing WT/*M*$^{-/+}$ guts does not abolish death of loser cells. Clones of WT cells were induced by *hs*-Flp recombination in *M*$^{-/+}$ guts, and Puc was continually expressed from the time of clone induction (PSwitch^{all}>Puc, +RU486, 13d ACI). Sytox positive cells (red) are still observed in *M*$^{-/+}$ neighboring WT cells (arrows). WT clones are marked by the absence of GFP (green) (*hsflp/+; PSwitch ^{ALL}/UAS Puc; FRT82B, ubiGFP, RpS3/FRT82B*).

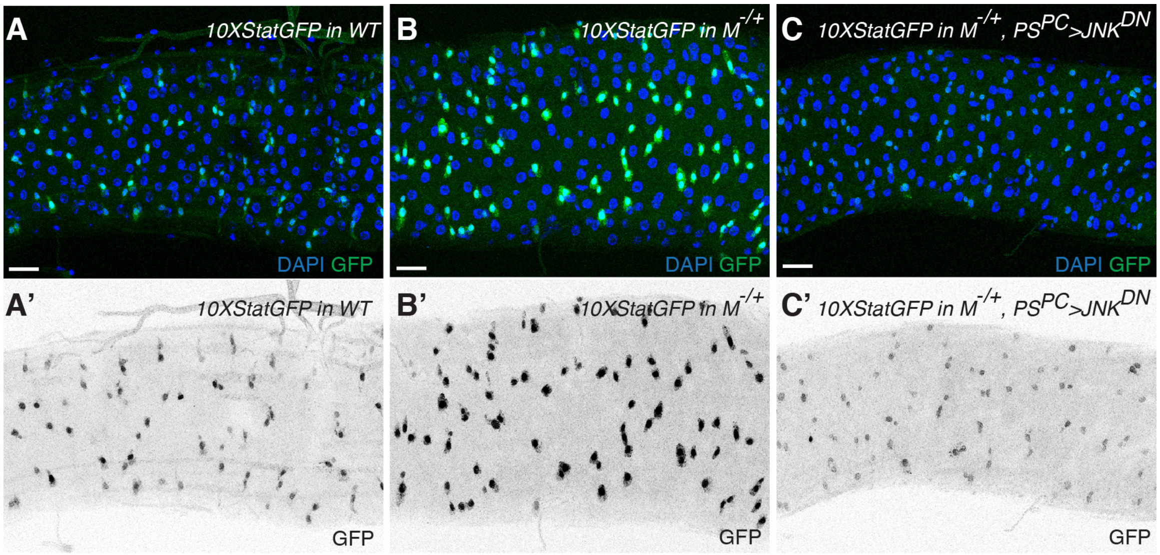

Figure S4 (related to Figure 6) JAK/STAT activation in *M*$^{-/+}$ cells is JNK-dependent.

(A-C) The 10XStat-GFP reporter is expressed at higher levels in *M*$^{-/+}$ guts (*10xStat-GFP/PSwitch*^{PC}*; FRT82, tubDsRed, RpS3*/+) (B-B') than in wild-type (*10xStat-GFP/PSwitch*^{PC}) guts (A-A'). Continued JNK^{DN} expression across ECs (by PSwitch^{PC} and RU486 supplementation) restores 10XStat-GFP expression to wild-type levels (*UAS Bsk*^{DN}/+; *10xStat-GFP/PSwitch*^{PC}*; FRT82, tubDsRed, RpS3*/+) (C-C', compare to A and B). Samples were equally aged (5 day old) and were processed and imaged in parallel. A'-C' display intensities in inverted gray levels.

Supplemental Experimental Procedures

Drosophila stocks:

yw

*Df(1)R194, w/ FM7; P[RpL36+w+]FRT40/ CyO (*N. Baker*;* the *Df(1)R194w* deficiency encompasses the RpL36 locus)

hsflp; FRT40, ubiGFP

hsflp; FRT82B/ TM6B

enGal4UASflp/ CyO; FRT82B, ubiGFP

FRT82B, ubiGFP, RpS3/TM6B (Bloomington)

FRT82B, tubDsRed

FRT82B, tubDsRed, RpS3/TM6B (recombinant generated for this study)

yw, hsflp; FRT40, M(2), arm-lacZ/ CyO

actGal4, UAS mCD8 Parp Venus (recombinant generated for this study from *UAS mCD8 Venus Parp* (D. Williams) and *actGal4/TM6B* (Bloomington)

MyoIAGal4, tubGal80^{ts} (recombinant generated for this study)

PSwitch^{AMP} (line 5961, Gal4 conditionally expressed in progenitor cells) (Mathur et al., 2010))

PSwitch^{PC} (line 5966, Gal4 conditionally expressed in enteroblasts and enterocytes cells) (Mathur et al., 2010)

PSwitch^{ALL} (this study, recombinant *PSwitch^{AMP}, PSwitch^{PC}*, used to express genes conditionally in progenitor cells and enterocytes*)*

UAS Puc^{14C} (E. Martin-Blanco)

UAS Bsk^{DN}(E. Martin-Blanco)

w; UAS Diap1/Cyo KrGal4, UASGFP; TM2/ TM6, Df YFP (P Meier and JP Vincent)

UAS dTCF^{DN} (JP Vincent)

hsflp, actGal4, UAS mCD8GFP; FRT40, tubGal80 (D. St Johnston)

hsflp, actGal4, UAS mCD8GFP/ FM7; P[RpL36+w+]FRT40 tubGal80/ CyO (recombinant and stock generated for this study)

FRT82B, RpS3/ TM6B* (Bloomington)

FRT82B, puc^{A251} *(lacZ)/ TM3* (E. Martin Blanco)

FRT40, ubiRFP (D. St Johnston)

Upd3Gal4, UAS GFP/ CyO (N. Perrimon)

10XStat-GFP (E. Bach)

w; UAS dome$^{\Delta cyt2}$*/ CyO*^{wglacZ} (J. Castelli Gair Hombria) *(aka* Dome^{DN} in the text)

w dome^{G0218} *FRT19A/ FM7c; eyFlp* (DGRC)

Drosophila genetics and stock maintenance

Flies were grown at 25°C and fed on standard fly food containing yeast. For experiments using the PSwitch system (Osterwalder et al., 2001), flies were raised on Nutrifly-GF (Dutscher Scientific UK Ltd 789234) with 200µM of mifepristone (RU486) (Sigma M8046) in EtOH 80% or an equal volume of EtOH 80% as control. For experiments using the TARGET system (McGuire et al., 2003; Figure S3E), flies were raised at 18°C and switched to 25°C after eclosion and heat shock. For Sytox feeding experiments, flies were kept at room temperature (22°C) for the duration of the feeding prior to dissection. Adults aged for guts preparation were transferred to fresh vials every 1-2 days and kept at 25°C.

Generation of mitotic clones

For clone generation in the gut, single stem-cell derived clones were generated by mitotic recombination, using the Flp/FRT system (Xu and Rubin, 1993). One to two days after eclosion, flies were heat-shocked (HS) in a water bath at 37°C and then reared at 25°C or as otherwise specified for the indicated time. Flies were aged up to a maximum of 20 days ACI, to avoid

ageing effects, which disrupt tissue homeostasis. Indeed we confirmed that at 20 day ACI, no increase in the number of ISCs (a hallmark of ageing) could be observed in $M^{-/+}$ or WT guts (data not shown). Heat-shock times were 10 minutes (for FRT82) or 30 minutes (for FRT40), except for Figure 2 A-D, where flies were heat-shocked for one hour.

Immunostaining

Guts were dissected in PBS and fixed for 20 minutes at room temperature on a shaker in a solution of PBS containing 3.7% Formaldehyde and 0.025% Triton X-100. After several washes in 0.25% Triton X-100 / PBS, guts were permeabilised for 30 minutes 1% Triton X-100 / PBS, then blocked for 30 minutes in a solution of 0.1% BSA, 0.1% Triton X-100 / PBS (blocking buffer). They were then incubated in the appropriate primary antibody diluted in blocking buffer, overnight at 4°C on a shaker. After several washes, guts were incubated for 2 hours at room temperature on a shaker with the appropriate secondary antibody. The following primary antibodies were used: mouse anti-Delta (DSHB, C594.9B) 1/1000, chicken anti-GFP (Abcam 13970) 1/1000, rabbit anti-phospho Histone 3 (ser10) (NEB, 9701S) 1/500, rabbit or mouse anti-cleaved human Parp (Abcam 2317, 1/25 and Abcam 110315, 1/500 respectively), rabbit anti-DsRed (Clontech 632496, 1/500), mouse anti-CD2 (Source Bioscience LS-C34521, 1/500), rabbit or chicken anti-β-Galactosidase (Cappel 55976, 1/400 and Abcam 9361, 1/1000, respectively). Secondary antibodies used were coupled to Alexa-488, Alexa 555 or Alexa-Cy5 (Molecular Probes). Nuclei were counterstained with DAPI or Hoechst. Samples were mounted in Vectashield (Vector laboratories) on a glass slide.

Confocal Acquisition and image analysis

Samples were imaged with an Olympus FV1000 Upright Confocal microscope, a Leica SP5 or a Leica SP8 confocal microscopes. All images were taken as z-stacks of 1μm sections in the

posterior midgut region immediately anterior to the hindgut (these corresponds to the regions P4 in (Marianes and Spradling, 2013); or region R5 in (Buchon et al., 2013)). Image processing, analysis and 3D reconstruction were done with Volocity (Perkin Elmer5.4.2) and Adobe Photoshop. For Figure 5A-B, in order to compare β-Galactosidase fluorescence intensity, samples were processed in parallel from eclosion of the flies to fixation, immunostaining and confocal acquisition. Similarly, guts for Figures 6A-B and S4A-C were processed in parallel. Note that the 10XStat-GFP reporter is sometimes highly upregulated in guts (including the visceral muscles) possibly due to sporadic infections. Such guts were not included in the analysis.

DSS feeding

Flies were transferred to an empty vial containing a piece of Whatman paper soaked with a solution of 5% DSS, in 5% sucrose/water, or just 5% sucrose/water. Flies were fed DSS for 2 or 3 days at 25°C, with fresh solution added daily.

Biophysical modeling

The quantitative analysis of the clonal fate data relies upon the development of a biophysical modeling scheme, which was previously formulated for the study of homeostatic turnover of the *Drosophila* wild-type posterior midgut (de Navascues et al., 2012). Briefly, following de Navascues et al., 2012, to model the dynamics of stem cells and their differentiated progeny, we considered a simple lattice model in which ISCs form a single equipotent population that are distributed uniformly within the epithelium. Alongside ISCs, each lattice site was associated with a fixed number of differentiating cells. The precise number of these differentiated cell types is fixed and discussed below. For convenience, we supposed that differentiating cells are organized as a hierarchy in which cells are lost in the order in which they mature. To model turnover we adopted an approach based on stochastic simulation in which a mature differentiated cell is

chosen at random and removed. Following its loss, with a given probability – one of only two parameters of the model, either the ISC on the same site undergoes asymmetric cell division giving rise to a replacement EB, or the ISC commits to EB cell fate and is itself replaced by the symmetrical duplication of an ISC at a neighboring site. As previously shown (de Navascues et al., 2012; Klein and Simons, 2011), in such a two-dimensional model system, the average size of surviving clones, $<n(t)>$, is predicted to rise approximately linearly with time, while the clone size distribution takes an exponential form. More precisely, the cumulative clone size distribution, defined as the probability, $P_n(t)$, of finding a clone with a size of more than n cells, takes the form $P_n(t)=\mathrm{Exp}[-n/n(t)]$.

Modeling control wild-type clones and control $M^{-/+}$ clones.

Following induction, clones were scored at 3, 7, and 20 days post-labeling both by the number of cells that were Dl+ and by total cell number. The process of labeling by mitotic recombination in addition to labeling ISCs generates a large number of single labeled EBs, which have to be removed from the dataset for correct data analysis. Thus, we focused our analysis on the ensemble of clones with two cells or more, which must therefore be derived from the labeling of ISCs. In both wild-type and the $M^{-/+}$ control, the cumulative size distribution showed convergence to exponential form, as predicted by the model (Figure 4). In the uniform background of wild-type clones in wild-type tissue or $M^{+/-}$ clones in $M^{+/-}$ tissue, the model depends on just three adjustable parameters, the ISC division rate, the frequency of ISC loss and replacement, and the relative fraction of ISCs to differentiating cells. To *fit* the model to the data, we first placed emphasis on the number of Dl+ cells per clone as a measure of clone size (Figure 4, insets), because this depends on only the first two of the three parameters, and is more immune to statistical noise. In doing so, we also circumvented the need to consider potential adjustment in the relative abundance of ISCs and differentiated cells, which may occur as a result of ageing. Moreover, to allow for potential adjustments in the cell proliferation rate over time, we made a fit of the model at each of the three separate chase times (3, 7, 20 days) to the expected number of

rounds of cell division, i.e. we did not impose a linear relationship between the chase time and the number of rounds of division, as would be expected if the division rate stayed constant. Using a least-squares fitting procedure, we found that the data for both wild-type and the *M*$^{-/+}$ control was consistent with a loss/replacement rate of around once per five divisions with the remaining divisions leading to asymmetrical fate outcome, figures consistent with that found by (de Navascues et al., 2012). In particular, with a constant ISC division rate fixed at 0.7 per day for the wild-type control and 0.35 per day for the *Minute*$^{-/+}$ control (consistent with the experimentally measured relative difference in proliferation rate between the two genotypes; Figure S2C), we found that the model could reproduce the size distribution at both the 3 and 7 day time points (Figure 4A insets). Note that, in the two-dimensional parameter space of ISC division rate and loss/replacement frequency, a variation of around 10% in either gives a theoretical prediction that would be contained within the experimental error bars, defined as the standard error of the mean. Further, note that the inferred division rates are likely to represent a slight underestimate, as they do not include the initial round of division that generated the first labelled cell upon mitotic recombination.

With the ISC division rate and the frequency of ISC loss/replacement fixed by the Dl clone size assay, we then attempted to predict the total clone size distribution. We adjusted the relative number of ISCs as a fraction of the total population to around one in 9±1, This estimate agrees well with the direct measurements of the fraction of Dl+ cells to DAPI stained nuclei, which shows that each ISC is associated with some 8 ± 0.5 differentiating cells in both the wild-type and *M*$^{-/+}$ control (data not shown). With this adjustment we found that the model could provide a good prediction of the experimental data over all three chase times (Figure 4, main graphs). A more careful scrutiny of the clone size distributions shows that, although there is generally good agreement of the model predictions with the experimental data at the 20 day time point, there is also evidence of departure for the largest clone sizes. These apparent discrepancies likely reflect

clone fusion (which we have observed frequently in the two-clone assay (Figure 2A-C) or acute proliferative response following local damage of the epithelium.

Modeling competing wild-type clones in a $M^{-/+}$ background.

To model competing clones we looked for a minimal adaptation of the model that could capture the observed behavior. Specifically, in-line with the experimental data from Figure 3, we introduced a moderate (25%±5%) increase in the number of divisions in wild-type cells and a corresponding increase in the loss rate of $M^{-/+}$ cells on the boundary of the clone, leaving all other aspects of the cell dynamics and fate behavior unchanged. With these parameters, we found that the model could provide a quantitative prediction of the cumulative clone size distribution, reproducing both the frequencies of Dl+ cell clones and the total clone sizes (Figure 4B).

In the $M^{-/+}$ control, the cell division rate (and, therefore, the loss rate) are approximately half that of the wild-type control. Therefore, in competing conditions, the inferred increase of 25% in the loss rate of boundary $M^{-/+}$ cells translates to a ~2.5 fold increase with respect to the non competing $M^{-/+}$ control. We note that this figure represents the average loss rate increase experienced across each $M^{-/+}$ cell contained in the 9-cell units that contact wild-type cells. Given that cell competition is a short-range interaction and that wild-type clones actually contact only a small fraction of cells within each unit, it is highly likely that the burden of cell loss increase is not uniform within the unit but rather born by a small number of cells. If we suppose that, in the "lattice" of units, only an average of two cells/unit is affected by contact induced loss, these cells will experience an increase in loss rate that corresponds to a figure some 2.5*9/2=11-fold higher than control *Minute*$^{-/+}$ cells.

Supplemental References

Buchon, N., Osman, D., David, F.P., Fang, H.Y., Boquete, J.P., Deplancke, B., and Lemaitre, B. (2013). Morphological and molecular characterization of adult midgut compartmentalization in Drosophila. Cell Rep *3*, 1725-1738.

Marianes, A., and Spradling, A.C. (2013). Physiological and stem cell compartmentalization within the Drosophila midgut. Elife *2*, e00886.

McGuire, S.E., Le, P.T., Osborn, A.J., Matsumoto, K., and Davis, R.L. (2003). Spatiotemporal rescue of memory dysfunction in Drosophila. Science *302*, 1765-1768.

Osterwalder, T., Yoon, K.S., White, B.H., and Keshishian, H. (2001). A conditional tissue-specific transgene expression system using inducible GAL4. Proc Natl Acad Sci U S A *98*, 12596-12601.

Xu, T., and Rubin, G.M. (1993). Analysis of genetic mosaics in developing and adult Drosophila tissues. Development *117*, 1223-1237.
